# Mapping Use of High Dose or Long‐Term Oral Glucocorticoids and Steroid‐Sparing Strategies in Adults With Chronic Conditions: A Rapid Scoping Review of Reviews

**DOI:** 10.1002/pds.70233

**Published:** 2025-10-15

**Authors:** Elizabeth Moore, Mohammad Al Sallakh, Olufemi Olajide, Daira Trusinska, Tracy Jackson, Ting Shi, Sara J. Brown, Joanna C. Robson, Mwidimi Ndosi, Meghna Jani, Sir Aziz Sheikh, Constantinos Kallis, Stephanie J. Lax, Jennifer K. Quint

**Affiliations:** ^1^ School of Public Health Imperial College London London UK; ^2^ Population Data Science Swansea University Swansea UK; ^3^ Alder Hey Children's Hospital Trust Liverpool UK; ^4^ The University of Edinburgh Edinburgh UK; ^5^ School of Health and Social Wellbeing, Centre for Health and Clinical Research, University of the West of England (UWE) Bristol Bristol UK; ^6^ Rheumatology Department, University Hospitals Bristol and Weston NHS Foundation Trust Bristol UK; ^7^ Centre for Epidemiology Versus Arthritis, Centre for Musculoskeletal Research The University of Manchester Manchester UK; ^8^ Nuffield Department of Primary Care Health Sciences University of Oxford Oxford UK; ^9^ Department of Lifespan and Population Health School of Medicine, University of Nottingham Nottingham UK

**Keywords:** adverse events, chronic, glucocorticoids, steroid sparing, tapering

## Abstract

**Purpose:**

Oral glucocorticoids (OGCs) have a broad range of uses and are effective in treating numerous conditions. However, it is commonly acknowledged that OGCs at high doses or over long periods have a burden of toxicity. Despite the use of steroid‐sparing therapies, OGCs continue to be prescribed to treat a wide range of immune and inflammatory conditions. We aimed to address the following research questions: (1) what are the contemporary indications for high dose and/or long‐term OGCs? (2) what patterns of use are described in the literature for high dose and/or long‐term OGCs? (3) which evidence do we have for chronic conditions related to well‐established steroid‐sparing strategies and tapering regimes for OGCs? (4) what adverse effects have been reported with high dose and/or long‐term OGCs?

**Methods:**

A rapid scoping review was conducted using the Joanna Briggs Institute guidelines. The Protocol has been published on the Open Science Framework. A systematic search of MEDLINE (Sep 2014 to Sep 2024) identified systematic reviews and scoping reviews involving adults (≥ 18 years) treated with high doses and/or long‐term OGCs for chronic inflammatory conditions. Studies involving pregnant women were excluded.

**Results:**

In total, 137 reviews were included. OGCs were indicated in 47 different conditions in dermatology, respiratory, gastrointestinal, hematology, immunology, rheumatology, and other miscellaneous categories. Across all specialties, OGCs were used either at high doses (at least 20 mg prednisone equivalent per day) or for long durations (for at least 3 months). For types of adverse effects reported in the included reviews, 20 were labeled as endocrine, 13 as immunological, 21 as musculoskeletal, 30 as gastrointestinal, and 16 as cardiovascular. Sixty‐four reviews looked for/reported unspecified adverse events. One hundred and fifteen reviews had evidence of steroid‐sparing/tapering regimes, indicating the wide use of these strategies to mitigate the harmful effects of OGCs.

**Conclusion:**

OGCs are used for a broad range of inflammatory conditions across multiple specialties. There is evidence related to a broad range of potential adverse effects across multiple body systems regardless of the indication of use. Further research is needed using a combined cross‐condition approach to their measurement and reduction, alongside gaining more insight into the impact of OGCs on patients' quality of life.

**Summary**

- This review aimed to map the evidence for OCG patterns of use in chronic conditions and where steroid‐sparing strategies are used to mitigate any harmful effects of OGCs.
- Adults (≥ 18 years) treated for chronic inflammatory conditions with high dose OGCs (at least 20 mg prednisone equivalent per day) or long‐term OGCs (at least 3 months) were included.
- 137 reviews met the inclusion criteria and OGCs were indicated in 47 different conditions across numerous specialities.
- A broad range of potential adverse effects were found across multiple body systems regardless of indication of use and 115 reviews had evidence of steroid‐sparing strategies.

## 1 Introduction

Oral glucocorticoids (OGCs) are widely used in the management of a wide range of immune and inflammatory conditions due to their potent and versatile pharmacological effects, flexible administration, wide availability, and affordability. Common chronic conditions for which they are used include asthma, giant cell arteritis, inflammatory bowel disease, multiple sclerosis, rheumatoid arthritis, and ulcerative colitis. OGCs are highly beneficial as they work swiftly in potentially life and organ‐threatening disease and can be used to treat flares of chronic conditions. However, patients have mixed views on the use of OGCs in that they recognize the fast‐acting and effective benefits but have concerns about their adverse effects (AEs) and the uncertainty of the dose‐reduction process [1]. Furthermore, patients have concerns regarding the impacts of systemic glucocorticoids on their weight and appearance [2].

Due to their non‐specific and systemic pharmacological effects, therapeutic doses of OGCs are often associated with an increased risk of a wide range of AEs including, but not limited to, metabolic disturbances, musculoskeletal, psychiatric, cognitive, gastrointestinal, and cardiovascular complications as well as adrenal insufficiency and unwanted immune suppression [3, 4, 5]. Furthermore, accumulating evidence suggests concerning patterns of over‐use [6, 7], misuse [8, 9], and resistance to glucocorticoids [10]. The reliance on OGCs therefore imposes a substantial clinical, societal, and financial burden and research has been carried out to consider the perspectives of patients who use them. In a modified Delphi study, the Outcome Measures in Rheumatology (OMERACT) Glucocorticoid Impact working group identified domains that are of greatest importance to both patients and healthcare professionals, and these include: bone fragility, diabetes, eye problems and/or changes in vision, high blood pressure, infection, osteonecrosis, mood disturbance, fatigue, sleep disturbance, and weight [11]. In addition, the OMERACT group also reviewed perspectives of patients with a broad range of inflammatory diseases and found that physical symptoms, psychological symptoms, effect on participation, and contextual factors are key themes that are of importance to them [12].

A brief overview of the literature by Gruszka et al. [13] recently stated that OGCs are among the most commonly prescribed medicines across numerous medical specialties. However, they can lead to a greater number of side effects due to their systemic effects. With rapidly evolving clinical practice and increasing numbers of potentially steroid‐sparing treatments available for specific conditions, there is a need for up‐to‐date evidence about the utilization of OGCs and the extent of their harm. A scoping review provides a broad overview of a research topic to map the existing evidence and provide insight into research gaps to help provide directions for future research [14]. Done rapidly, they can provide timely overviews of important clinical topics to help guide clinicians and researchers. Initial search development demonstrated a vast and heterogeneous literature including a number of systematic reviews in scope for the research question. Therefore, to ensure feasibility, we restricted the scope of the review to systematic reviews only. An initial search of MEDLINE returned many systematic reviews about efficacy, effectiveness, safety, and long‐term consequences of OGCs use [4, 5, 15, 16, 17, 18]. However, to our knowledge, there were no scoping reviews on contemporaneous indications of OGCs currently underway. The objective of this scoping review was to identify evidence for major trends in the use of OGCs and efforts to reduce associated harm through steroid‐sparing strategies. We aimed to address the following research questions:

- What are the indications for high dose and/or long‐term OGCs according to evidence from systematic and scoping reviews?
- What patterns of use are for long‐term and/or high‐dose OGCs?
- Which chronic health conditions have well‐established steroid‐sparing strategies and tapering regimes?
- What AEs have been looked for/reported in systematic and scoping reviews of high dose and/or long‐term OGCs?

## 2 Materials and Methods

This scoping review was conducted in accordance with the Joanna Briggs Institute methodology for scoping reviews [19] and is reported using the PRISMA extension for scoping reviews [20]. The protocol has been published on the Open Science Framework [21].

### 2.1 Eligibility Criteria

The eligibility criteria were structured using the Population–Concept–Context (PCC) framework.

#### 2.1.1 Population

Adults (≥ 18 years) who use OGCs to treat chronic inflammatory conditions were included. Studies involving pregnant women were excluded in the review as a pragmatic decision to make the rapid review manageable as these findings would need to be analysed separately.

#### 2.1.2 Concept

The main concept was “high use of oral glucocorticoids”. For this review, we defined high use of OGCs as long‐term and/or high dose use. We defined long‐term use as regular (e.g., daily) use of OGCs for at least 3 months. This definition is based upon prevalence and prescription patterns of OGCs in adults [22]. In addition, we defined a high dose of OGCs as at least 20 mg prednisone equivalent per day [23]. These definitions have been adopted by several studies [24, 25, 26]. The other concepts include long‐term complications of OGC use [27] and steroid‐sparing strategies [28].

#### 2.1.3 Context

Studies conducted in any healthcare setting or country were eligible for inclusion to maximize global relevance and capture variations in clinical practice.

### 2.2 Information Sources

The search for eligible reviews was conducted MEDLINE (PubMed) and limited to reviews published from September 2014 to September 2024. This 10‐year period was a pragmatic decision, selected to capture evidence about current and most recent patterns of OGC use. The search was restricted to reviews published in the English language due to resource limitations for translation.

### 2.3 Search

The search strategy was developed iteratively and followed the three‐step process recommended by JBI for scoping reviews. An initial limited MEDLINE (PubMed) search was performed using broad keywords related to “oral glucocorticoids”, “chronic inflammatory conditions” and “high use” (Data S1). This informed development of the full search strategy in MEDLINE. The MeSH terms and keywords from the titles and abstracts of the retrieved articles were examined to iteratively improve the precision and recall of the search query. Finally, backward citation searching was carried out to search for additional eligible studies. The final search strategy is given in Data S2.

### 2.4 Study Selection

Following the full search, records were collated and uploaded into Covidence, a web‐based systematic review platform (Veritas Health Innovation, Melbourne, Australia; available at www.covidence.org), and duplicates were removed. Titles and abstracts were screened by six independent reviewers and were assessed against the inclusion/exclusion criteria for our review (Data S3). The full texts of selected citations were assessed in detail against the selection criteria by at least two independent reviewers. Reasons for exclusion were recorded and are reported in the PRISMA flow chart, with individual judgments reported in Data S5. Any disagreements that arose between the reviewers were resolved through discussion and were adjudicated by an additional reviewer with clinical expertise where necessary.

### 2.5 Data Extraction

Data were extracted from papers included in the scoping review by a single reviewer (either EM, MAS, OO, DT, or JKQ) using a bespoke data extraction form that had been piloted by the team (Data S4). Data extracted included the following details, where available:

- Objective of the systematic/scoping review
- Study design (of studies included in the systematic reviews)
- The number of studies included (randomized controlled trials (RCTs), observational studies, and/or other study designs).
- Geographical locations and healthcare settings
- Whether ethnicity was reported
- Indication for OGCs
- Evidence of high dose OGC use
- Evidence of long‐term OGC use
- Evidence of steroid‐sparing and tapering regimes
- AEs/toxicity profile.

Each included review had an individual data extraction form within Covidence systematic review software (Veritas Health Innovation, Melbourne, Australia) into which reviewers entered data items. Each extraction was then checked by a second reviewer for accuracy (SJL), and disagreements were discussed with the clinical lead where necessary (JKQ). Data items were exported from Covidence to Microsoft Excel (Microsoft Corporation, Redmond, Washington, USA) for analysis. Differences between protocol and review are described and justified in Table S1.

During the writing phase, reporting of adverse events was simplified, with pseudonyms and alternative spellings relabeled to one consistent term. Categories were also assigned according to body system (e.g., infection, immunological, musculoskeletal). To achieve this, a semi‐automated process was used to generate a lookup table by separating the lists of adverse events extracted by common delimiters. A reviewer then spot‐checked the matching and iterated the list until all adverse events were included. The adverse event categorization is summarized in Table S4. Data management and visualizations were conducted using R version 4.4.2 [29].

## 3 Results

**FIGURE 1:**
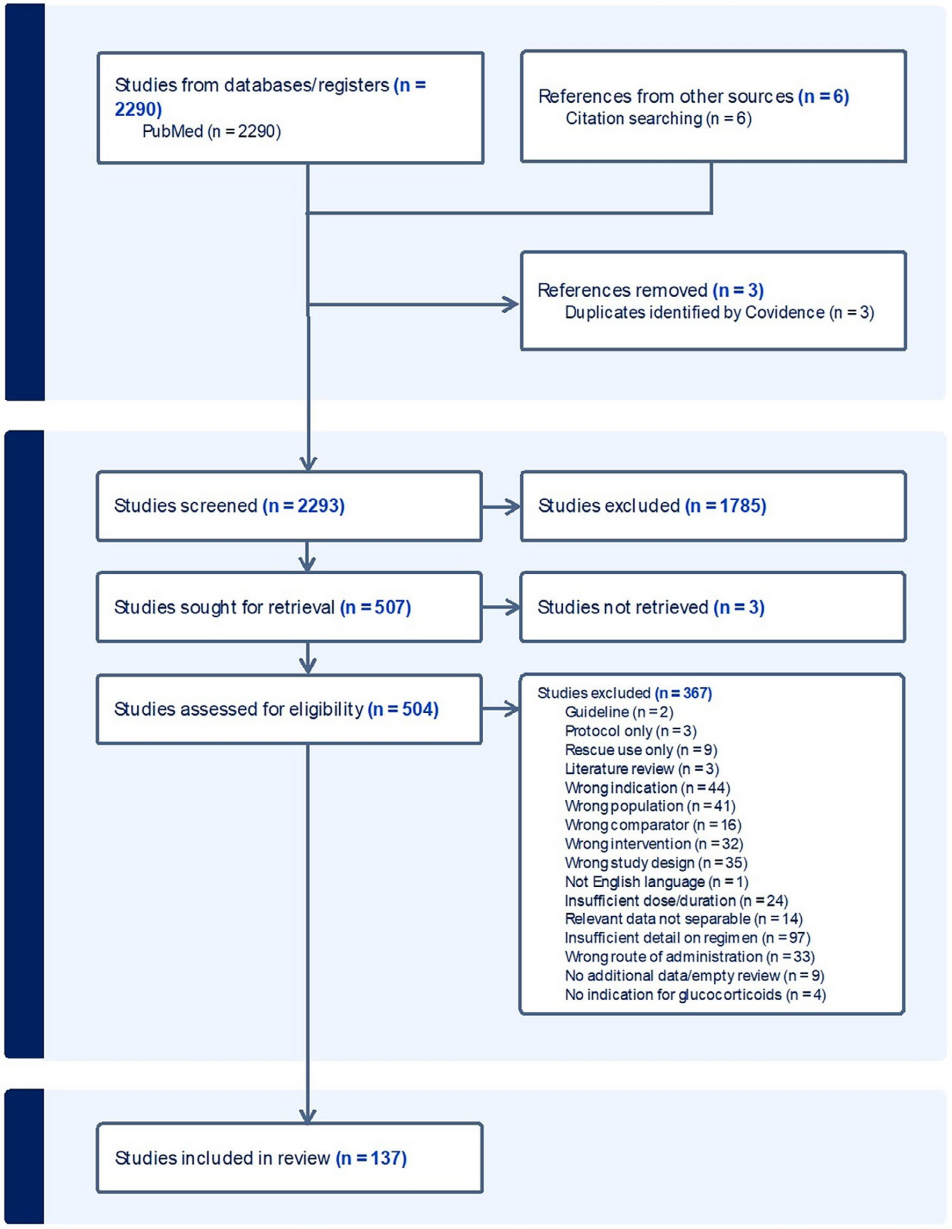
Study flowchart.

Of 2293 records screened, 137 reviews met the criteria for full data extraction (Figure 1). Reasons for exclusion at full text screening are shown in Figure 1, with full references given in Data S5. Full references for included reviews are also given in Data S6. Table S2 lists characteristics of the included reviews. Where specified, 87 reviews included data from high‐income countries, 44 reported data from middle‐income countries, and none from low‐income countries. In terms of healthcare settings, 32 reviews included data from secondary care, 10 from primary care, and 9 collected data from “other” settings. Ethnicity was only recorded in 11 out of 137 studies, highlighting the need for more reporting of ethnicity in such studies. In terms of the types of reviews included in our scope, there were 135 systematic reviews, 1 scoping review, and 1 overview of systematic reviews. Fifty‐seven reviews included data in scope for this review from only RCTs, 37 reviews included data from observational studies, and 29 reviews included both RCTs and observational studies. The remaining reviews included other types of research including non‐randomized interventional studies, open‐label studies, systematic reviews, or unclear study designs.

### 3.1 Indications for High Dose and/or Long‐Term OGCs According to Evidence From Systematic and Scoping Reviews

**FIGURE 2:**
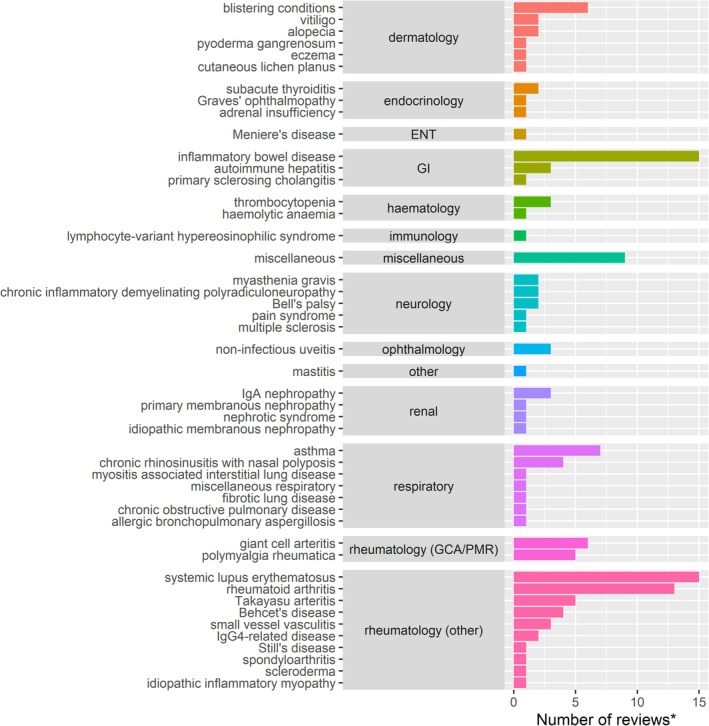
Number of systematic or scoping reviews found reviewing OGC use by indication. Abbreviations: ENT: Ear, Nose, and Throat; GCA: Giant cell arteritis; GI: Gastrointestinal; PMR: Polymyalgia rheumatica; Ig: Immunoglobulin.

Figure 2 and Table S3 show the summary of indications for OGC use and the number of reviews addressing each indication. The use of OGCs was indicated in 47 different conditions across a variety of specialties, including rheumatology, dermatology, respiratory, gastrointestinal, hematology, immunology, and other miscellaneous categories. A large proportion of reviews in which OGC use was indicated were rheumatological: 15 for systemic lupus erythematosus (SLE), 13 for rheumatoid arthritis (RA), 5 for Takayasu arteritis, 6 for giant cell arteritis (GCA), 5 for polymyalgia rheumatica (PMR), and 13 for other rheumatological conditions (Behcet's disease, small vessel vasculitis, spondyloarthritis, IgG4‐related disease, Still's disease, scleroderma, and idiopathic inflammatory myopathy). Inflammatory bowel disease (IBD) was another prominent category with 15 reviews of IBD indicating the use of OGCs. There were 7 reviews for asthma, 6 reviews for blistering conditions, and 9 reviews for “miscellaneous” indications. The results found in our extensive literature search highlighted the broad use of OGCs across many different specialties.

### 3.2 Patterns of Use Apparent in the Literature for High Dose and/or Long‐Term OGCs

**FIGURE 3:**
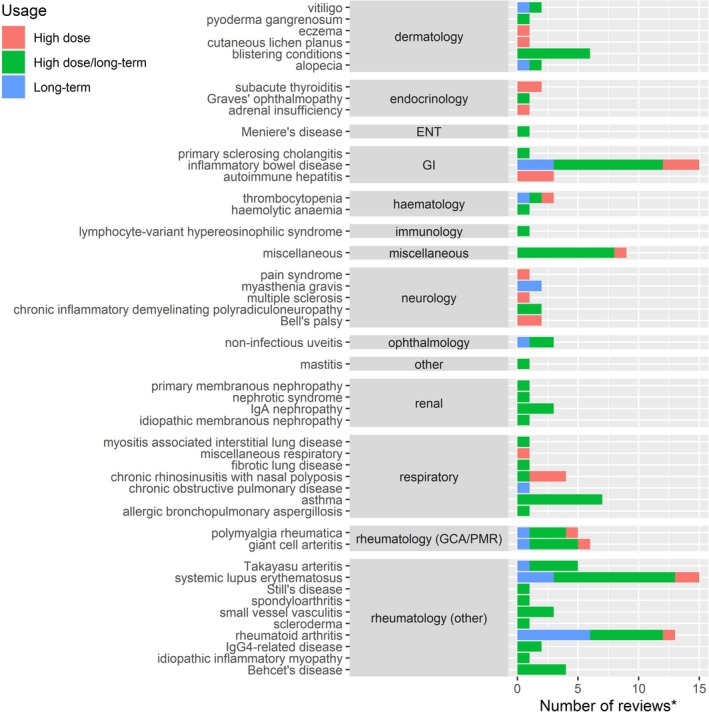
Number of systematic or scoping reviews found in our literature search looking for high dose and/or long‐term OGC use by indication. Abbreviations: ENT: Ear, Nose, and Throat; GCA: Giant cell arteritis; GI: Gastrointestinal; PMR: Polymyalgia rheumatica; Ig: Immunoglobulin.

The balance between use of high dose and long‐term OGC use by indication is shown in Figure 3. Ninety‐two reviews had evidence of both high dose and long‐term OGC use (one with duration > 1 month), 24 reviews examined the use of high dose OGCs only, and 21 reviews examined long‐term OGC use only. Across all specialty domains, OGCs were used either at high doses or for long durations (indicated in green). In systematic reviews of renal conditions, we found high dose and long‐term use of OGCs in all conditions. However, in other specialties (e.g., neurology), the use of OGCs varied depending on the condition.

### 3.3 Chronic Health Conditions Apparent in the Research Literature That Have Well Established Steroid‐Sparing Strategies and Tapering Regimes

One hundred and fifteen reviews had evidence of steroid‐sparing strategies, which included tapering regimes. Eighteen reviews had evidence of steroid‐sparing strategies not including tapering regimes. Twenty‐two reviews out of 137 had no evidence of any steroid‐sparing strategies.

**FIGURE 4:**
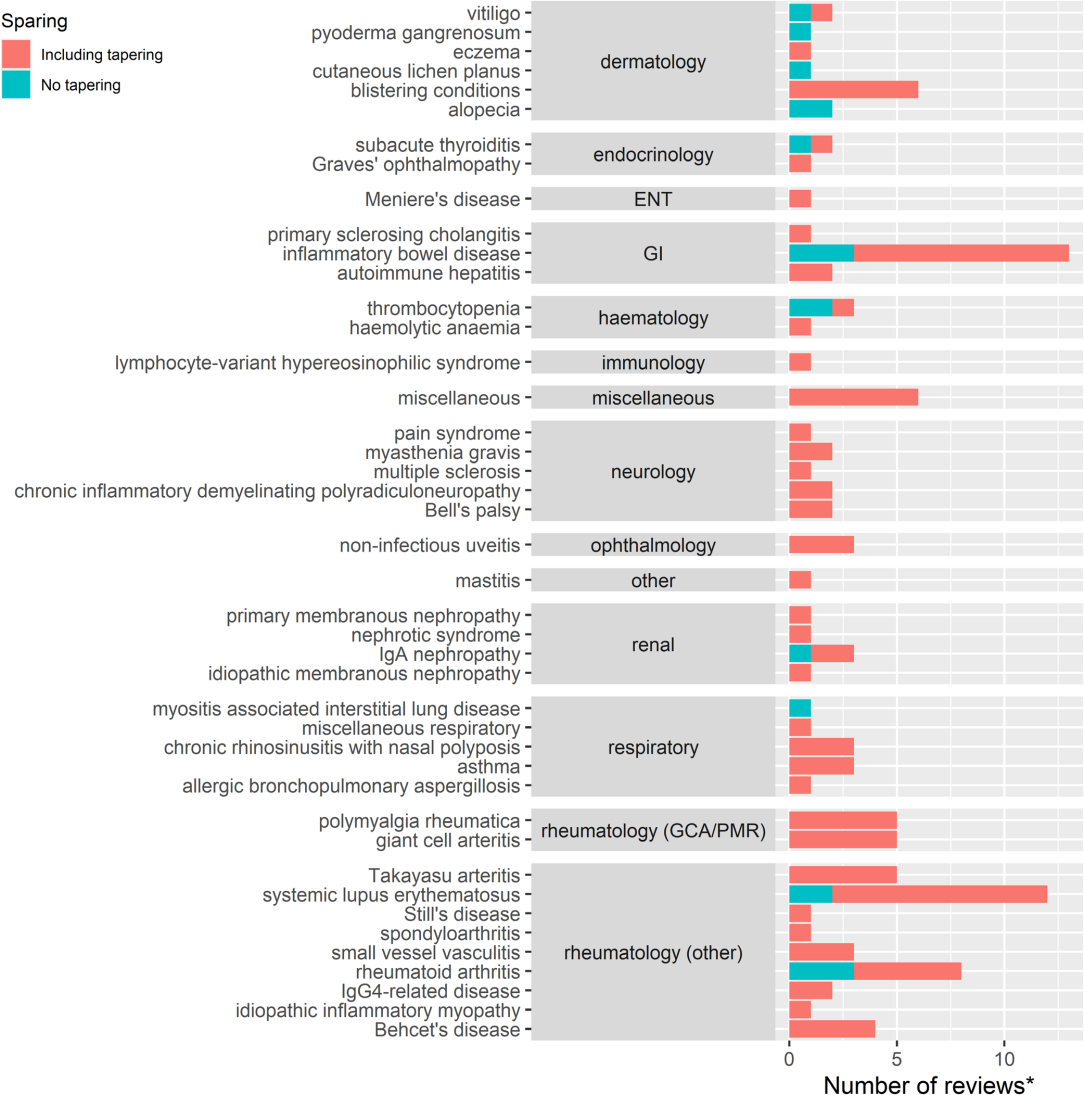
Number of systematic or scoping reviews found that examined steroid‐sparing (with or without tapering) by indication. Abbreviations: ENT: Ear, Nose, and Throat; GCA: Giant cell arteritis; GI: Gastrointestinal; PMR: Polymyalgia rheumatica; Ig: Immunoglobulin.

Figure 4 illustrates the use of steroid‐sparing strategies by indication and whether tapering was included or not. Our scoping review found that steroid‐sparing strategies including tapering are widely used across different specialties using a variety of agents. In neurology, ophthalmology, and ear, nose, and throat (ENT) specialties, OGC tapering was included in all the reviews that studied steroid‐sparing. However, in systematic reviews examining dermatology treatments, there were some conditions (pyoderma gangrenosum, cutaneous lichen planus, and alopecia) in which we did not find evidence of tapering as a steroid‐sparing strategy. Myositis‐associated interstitial lung disease was the only other condition in our scoping review for which we did not find evidence of tapering as a steroid‐sparing strategy.

### 3.4 Adverse Effects Looked for/Reported in Systematic and Scoping Reviews of High Dose and/or Long‐Term OGCs

**FIGURE 5:**
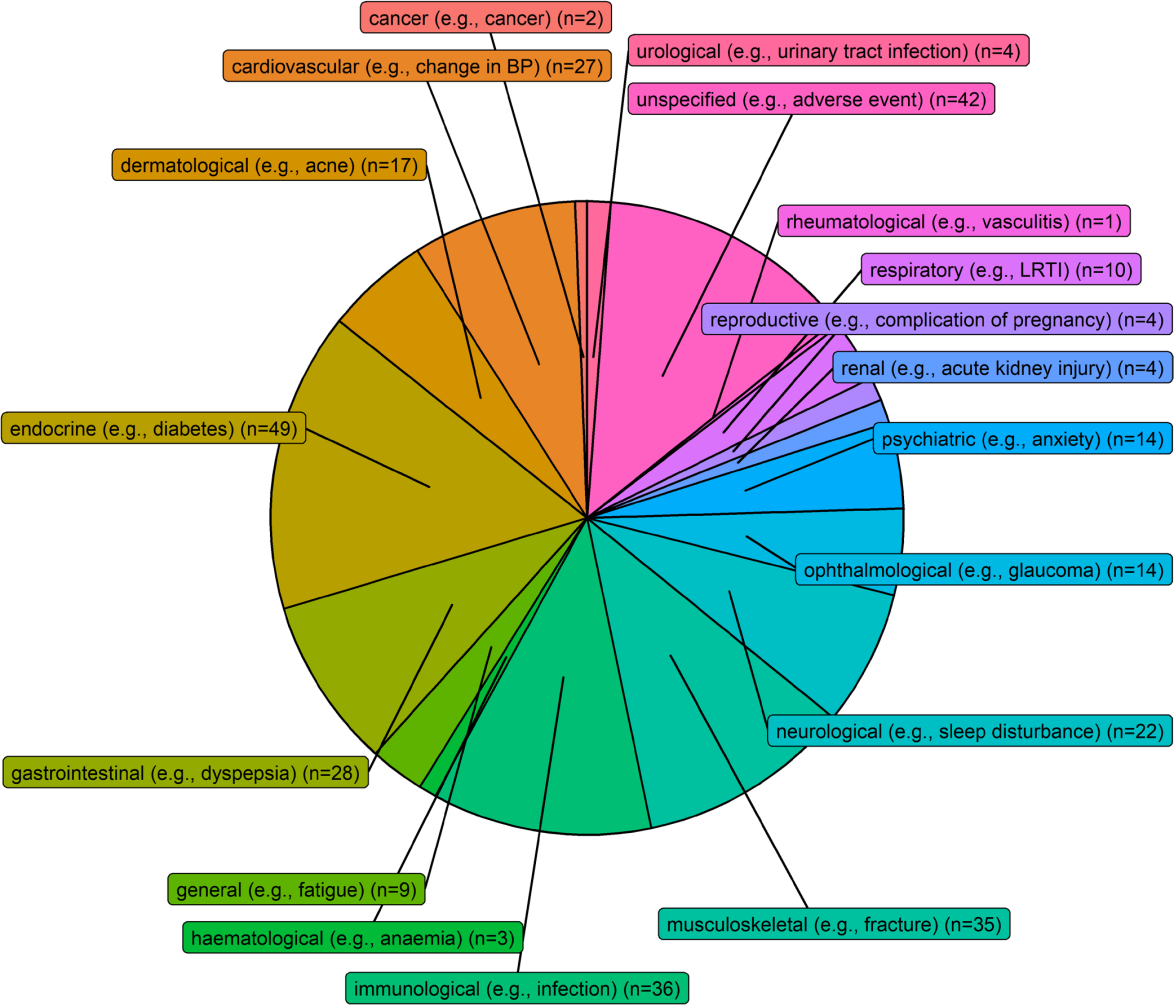
Number of systematic or scoping reviews found that reported/looked for adverse effects by type or body system.

A large number of AEs were looked for with OGC use, with a total of 169 AEs with labels such as “anxiety”, “headache”, “glaucoma”, “fracture” etc., and non‐specific labels such as “adverse event” and “serious adverse event” (complete list in Table S4). When scoping for AEs, 27 reviews mentioned “unspecified”, “steroid‐related”, or “glucocorticoid‐related” AEs, 12 reviews mentioned “serious unspecified” AEs, 12 reviews mentioned mortality as an AE, and 13 reviews mentioned “withdrawal from steroids due to unspecified AEs”. Figure 5 shows the number of AEs categorized by type/body system. The largest number of side effects was labeled endocrine (20 different types of AEs reported) and considerable numbers were labeled immunological (13 types of AEs), musculoskeletal (21 types of AEs), gastrointestinal (30 types of AEs), or cardiovascular (16 types of AEs). Sixty‐four reviews looked for/reported unspecified adverse events.

**FIGURE 6:**
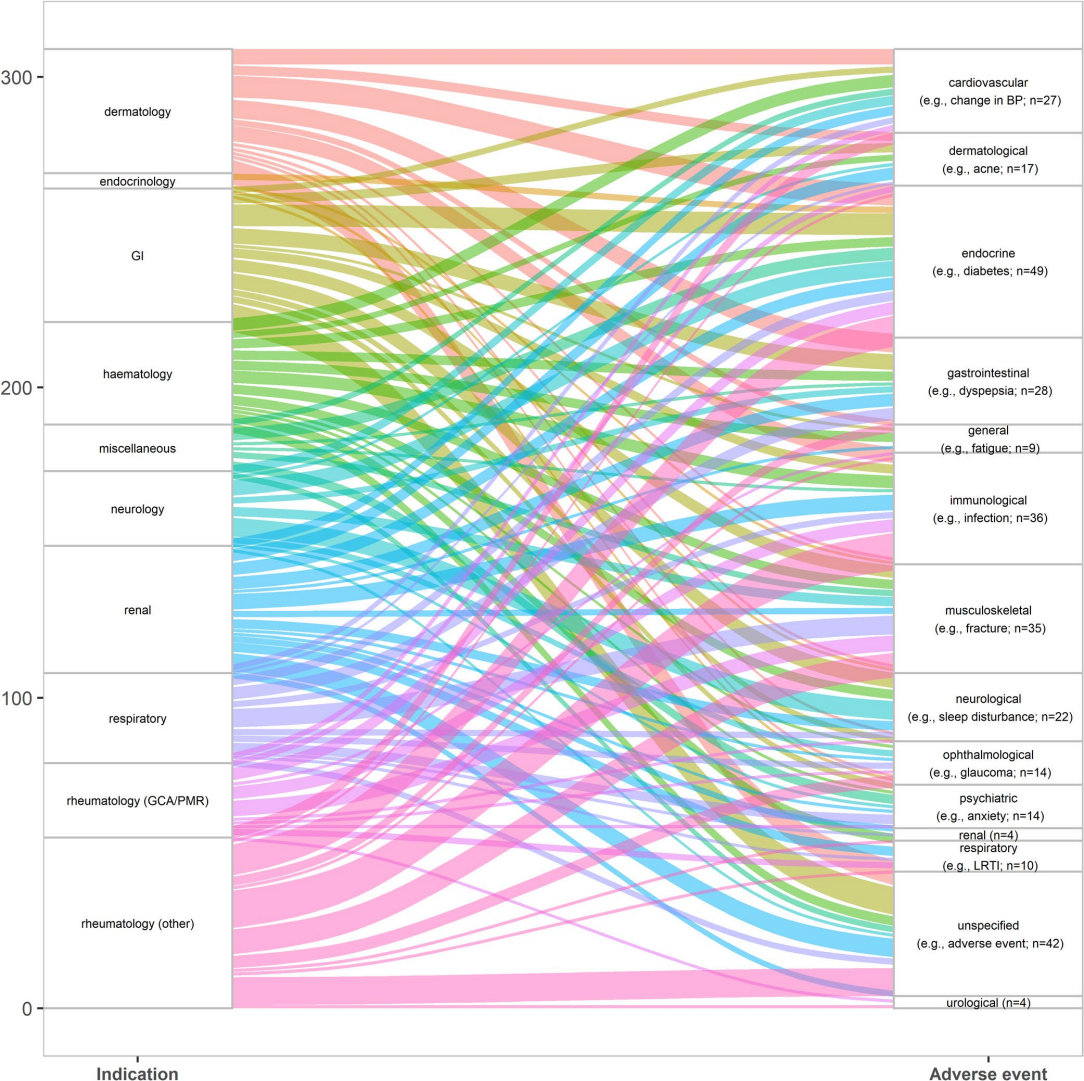
Associations between indications and adverse effects looked for/reported. Abbreviations: GCA: Giant cell arteritis; GI: Gastrointestinal; PMR: Polymyalgia rheumatica.

Figure 6 is a Sankey diagram with indications for OGCs on the left and categories of adverse events looked for/reported on the right. The width of the flows (i.e., ribbons of color) connecting these two variables represents the number of reviews addressing a given combination. It demonstrates that for conditions in which OGCs are prescribed, a variety of different adverse events have been looked for/reported, and there is considerable cross‐talk between indications and adverse events by type/body system.

## 4 Discussion

In this rapid scoping review, we have mapped evidence for contemporary OGC use and safety across a broad spectrum of inflammatory diseases. Our review revealed 47 different indications for the use of OGCs and found that although OGCs continue to be widely used, over 169 different potential AEs were mentioned in systematic reviews in relation to high dose and/or long‐term OGC use. These AEs of varying nature and severity were found across different domains and the highest number of adverse events were categorized as endocrine, immunological, musculoskeletal, gastrointestinal, and cardiovascular symptoms. In our scope of the literature, we found some of the well‐known safety risks of OGCs had been looked for/reported, including an increased risk of osteoporotic fractures, serious infections, diabetes, weight gain, musculoskeletal symptoms, gastrointestinal effects, headache, anxiety, high blood pressure, and mortality. Psychological side effects such as depression, anxiety, and mania were also mentioned [30], particularly in prednisolone doses > 40 mg [31]. Our review also found that there are no clear relationships between OGC indication and the mention of AEs, with no clear pattern between OGC indication and the side effects that were mentioned in reviews. Furthermore, many of the AEs that were looked for and found in our review were listed as “unspecified” or “serious unspecified,” and this could be related to the methods of collection in the primary research. However, it also highlights a gap in research to identify what these unspecified AEs might be and to determine the true safety profile of OGCs for each indication.

What remains unclear is the threshold for which the risks of long‐term and/or high doses of OGCs outweigh any therapeutic benefits. Determining the nature of the association between these potential adverse events and OGCs, i.e., the risk of developing them for a given individual, is beyond the remit of a scoping review such as this. In one included review, safety risks were found to increase with increasing dose and/or duration in the treatment of RA and long‐term OGC use was associated with vertebral fractures, even in low doses (< 10–15 mg/day) [32]. This illustrates the case for specific follow‐on of more focused systematic reviews with meta‐analyses in each area. The systematic reviews included in our scoping report primarily obtained data from high‐ and middle‐income countries, and only 11 studies provided data on ethnicity, highlighting a paucity of data on OGC use from lower‐income countries and the need for more information relating to ethnicity. The impact of OGCs on patients' quality of life is an important consideration and patient‐reported outcome questionnaires such as the Steroid PRO can be used to assess the treatment‐specific impact of OGC use [33].

In our review, nearly two‐thirds of the studies that examined the use of high doses and/or long‐term OGC use for chronic conditions mentioned steroid‐sparing strategies and/or tapering regimes. Steroid‐sparing therapies and tapering regimes have been explored to reduce the risk of steroid‐related side effects for a wide variety of conditions across numerous specialties, and some modalities have been shown to successfully reduce OGC doses. For example, the biological agent Tocilizumab used in combination with prednisone tapering was found to be superior to prednisone tapering in combination with a placebo for sustaining glucocorticoid‐free remission in patients with GCA [34]. In another example, Avacopan has been found to be superior to prednisone taper with respect to sustained remission at week 52 in the treatment of antineutrophil cytoplasmic antibody (ANCA)–associated vasculitis. However, longer trials are needed to determine its durability and safety [35].

Advances in potential or lack of targeted therapies for patients with inflammatory diseases exposed to OGCs should also be considered for future research. For example, the importance of predictive biomarkers in the management of inflammatory lung diseases such as chronic obstructive pulmonary disease (COPD) has been the focus of several researchers over the last decade. The effects of the stability of such a biomarker have been evaluated to help guide precision management of COPD patients [36, 37]. Furthermore, some guidelines for managing long‐term inflammatory diseases such as COPD, including the European Respiratory Society guidelines on withdrawal of inhaled corticosteroids, advocate withdrawal based on predefined biomarker (blood eosinophil) cut‐offs [38]. Future research could consider how predictive biomarkers could be used to guide precision management of other chronic inflammatory conditions, including how they might be used within tapering strategies.

Some limitations to our rapid scoping review apply to any scoping review, notably time constraints and missing out on some of the relevant primary literature [39]. For example, a comparison of OGC withdrawal strategies in RA patients with low disease activity [40]. Scoping reviews may also have inconsistencies in methodology, and guidelines are lacking to help authors conduct scoping reviews and standardize reporting [41]. In addition, individual systematic reviews also have limitations of their own, such as methodological challenges and limited scope [42]. There may be more detail in the primary literature than was reported in the systematic and/or scoping reviews. However, there are some limitations that are specific to this scoping review. Firstly, the exclusion of case series means that rare diseases (e.g., Takayasu arteritis and eosinophilic granulomatosis with polyangiitis) may not be represented sufficiently in this review. Future research could be undertaken using electronic health records to identify patterns of use of OGCs. Secondly, only observational and interventional reviews in adults were included. However, we found that age was sometimes poorly reported in the systematic reviews included, or it was reported as mean age without range or clear inclusion and exclusion criteria. It is therefore possible that some of the included evidence was contributed by pediatric patients. Thirdly, it was difficult to determine whether induction of remission was for the management of disease exacerbations or for general disease control, and this may vary by indication. For example, asthma and COPD studies were excluded unless they were specifically about disease control. Fourthly, some of the AEs listed may have been related to other agents being compared. For example, in a review examining the safety profile of Tocilizumab [43], some of the side effects listed may have been related to this agent rather than treatment with OGCs. Finally, while we excluded duplicate reviews and previous Cochrane reviews, there will be some duplication of the representation of the primary literature in a body of evidence of this magnitude.

## 5 Conclusions

Long‐term and/or high dose regimes of OGCs represent an important intervention in clinical medicine for the management of a broad range of conditions. However, this rapid scoping review of reviews has mapped evidence related to well‐known, and some less well appreciated AEs. Much of this evidence was found in reviews of OGC use in rheumatological conditions, and many of the AEs looked for or reported in the reviews were labeled as “unspecified”. There was considerable cross talk between OGC indication and the types of AEs looked for, indicating no clear patterns between conditions in which OGCs are indicated and the AEs that have been mentioned in reviews. Steroid‐sparing strategies and tapering regimes are widely used to mitigate the side effects of OGCs. OGCs are used for a broad range of inflammatory conditions across multiple specialties; their impact and AEs are broad‐ranging and affect people regardless of the indication of use. This rapid scoping review found that further research would be beneficial using a combined cross‐condition approach to their measurement and reduction, alongside gaining more insight into the impact on patients' quality of life.

## Ethics Statement

The authors have nothing to report.

## Consent

This is a scoping review of previously published literature and did not require any patient consent.

## Conflicts of Interest

Aziz Sheikh: Funding from HDR UK. Sara J. Brown holds a Wellcome Trust Senior Research Fellowship (ref 220 875/Z/20/Z). Meghna Jani is funded by a National Institute for Health and Care Research (NIHR) Advanced Fellowship [NIHR301413]. FAP and Stephanie J. Lax are funded by an NIHR Advanced Fellowship [NIHR300863] awarded to FAP. The views expressed in this publication are those of the authors and not necessarily those of the NIHR, NHS, or the UK Department of Health and Social Care. Joanna C. Robson holds a Sanofi Ltd. research grant for “Cross condition validation of the Steroid Pro” and a National Institute of Health and Care Research grant for the project titled “RAISE: self‐management in rare autoimmune rheumatic diseases.” She has royalties/licenses for Steroid PRO, AAVPRO, and GCAPRO and is a co‐inventor. IP is held on behalf of inventors by Oxford Innovation. Free for clinical and academic use. Mwidimi Ndosi: Member of the Academic Working Group on a Delphi study to explore the evolving role of Janus kinase inhibitors in rheumatic and musculoskeletal diseases. Mwidimi Ndosi holds a Vifor Pharmaceuticals independent academic grant to his institution for a study to develop and validate patient reported outcome measure of impact of steroids in patients with rheumatic autoimmune conditions: the Steroid PRO (role: co‐applicant). In addition, Mwidimi Ndosi holds an independent academic grant to his institution for a study to assess face validity and feasibility of the Steroid PRO in patients with inflammatory gastroenterology, respiratory and dermatology condition (Co‐applicant). Jennifer K. Quint has been supported by institutional research grants from UKRI, NIHR, Health Data Research UK, BI, AZ, Insmed, and has received personal fees for advisory board participation, consultancy, or speaking fees from GlaxoSmithKline and BI. The other authors declare no conflicts of interest.

## Supporting information


**Data S1:** Initial PubMed queries.
**Data S2:** Final search strategy.
**Data S3:** Article screening tools.
Title and abstract screening tool.
Full‐text eligibility criteria.
**Data S4:** Data extraction tool.
**Data S5:** Excluded studies and reasons for exclusion.
**Data S6:** References to included studies.
**Table S1:** Differences between protocol and review.
**Table S2:** Details of included studies.
**Table S3:** Studies by indication.
**Table S4:** Summary of adverse events.

**File S1:** Preferred reporting items for systematic reviews and meta‐analyses extension for scoping reviews (PRISMA‐ScR) checklist.

## Data Availability

The protocol for this scoping review has been published on the Open Science Framework and is available at https://osf.io/yzx79. The final data used for this study were extracted in June 2025 and are available upon request from the corresponding author. The manuscript is aligned with guidance from the PRISMA extension for scoping reviews (PRISMA‐ScR). Findings will be disseminated at relevant scientific conferences via presentation.
