# Supplementary material for: Mapping Use of High Dose or Long‐Term Oral Glucocorticoids and Steroid‐Sparing Strategies in Adults With Chronic Conditions: A Rapid Scoping Review of Reviews
Source: Pharmacoepidemiol Drug Saf. 2025 Oct 15;34(10):e70233. doi: 10.1002/pds.70233 (PMC12521885; doi:10.1002/pds.70233)
Supplement: Supplementary file 1 — Data S1: Initial PubMed queries. Data S2: Final search strategy. Data S3: Article screening tools. Title and abstract screening tool. Full‐text eligibility criteria. Data S4: Data extraction tool. Data S5: Excluded studies and reasons for exclusion. Data S6: References to included studies. Table S1: Differences between protocol and review. Table S2: Details of included studies. Table S3: Studies by indication. Table S4: Summary of adverse events. [file PDS-34-e70233-s001.docx]

**Supplementary materials**

Contents

[Data S1: Initial PubMed queries 2](#_Toc202861909)

[Data S2: Final search strategy 3](#_Toc202861910)

[Data S3: Article screening tools 6](#_Toc202861911)

[Title and abstract screening tool 6](#_Toc202861912)

[Full-text eligibility criteria 6](#_Toc202861913)

[Data S4: Data extraction tool 7](#_Toc202861914)

[Data S5: Excluded studies and reasons for exclusion 9](#_Toc202861915)

[Data S6: References to included studies 43](#_Toc202861916)

[Table S1: Differences between protocol and review 53](#_Toc202861917)

[Table S2: Details of included studies 54](#_Toc202861918)

[Table S3: Studies by indication 74](#_Toc202861919)

[Table S4: Summary of adverse events 77](#_Toc202861920)

# Data S1: Initial PubMed queries

Search date: 2024-08-27

**For adverse effects:**

    ("Glucocorticoids"[Mesh] OR Glucocorticoids[tiab] OR corticosteroids[tiab] OR steroids[tiab])

AND ("Glucocorticoids/adverse effects"[MeSH] OR "Drug-Related Side Effects and Adverse Reactions"[Mesh] OR adverse effects OR side effects OR toxicity)

AND "Systematic Review"[pt]

results: 2165

**For effectiveness/efficacy:**

    (steroids OR corticosteroids OR glucocorticoids)

AND (effectiveness or efficacy)

AND "Systematic Review"[pt]

results: 10199

# Data S2: Final search strategy

Database: PubMed/MEDLINE

Date of search: yyyy-mm-dd

Query:

(

"Glucocorticoids"[Mesh]

OR "Glucocorticoid*"[tiab]

OR steroid*[tiab]

OR corticosteroid*[tiab]

OR "Adrenal Cortex Hormones"[Mesh] OR "Adrenal Cortex Hormones"

OR "alclometasone"

OR "amcinonide"

OR "betamethasone" OR "betamethasone"[MeSH]

OR "beclomethasone" OR "beclomethasone"[MeSH]

OR "betamethasone" OR "betamethasone"[MeSH]

OR "budesonide" OR "budesonide"[MeSH]

OR "ciclesonide"

OR "clobetasol" OR "clobetasol"[MeSH]

OR "clocortolone"

OR "cortivazol"

OR "cortisone" OR "cortisone"[MeSH]

OR "deflazacort"

OR "desonide" OR "desonide"[MeSH]

OR "desoximetasone" OR "desoximetasone"[MeSH]

OR "dexamethasone" OR "dexamethasone"[MeSH]

OR "dichlorisone"

OR "diflorasone"

OR "diflucortolone" OR "diflucortolone"[MeSH]

OR "difluprednate"

OR "fluclorolone"

OR "flumethasone" OR "flumethasone"[MeSH]

OR "flunisolide"

OR "fluocinolone"

OR "fluocinonide" OR "fluocinonide"[MeSH]

OR "fluocortolone" OR "fluocortolone"[MeSH]

OR "fluorometholone" OR "fluorometholone"[MeSH]

OR "fluprednidene"

OR "fluprednisolone" OR "fluprednisolone"[MeSH]

OR "flurandrenolide"

OR "fluticasone" OR "fluticasone"[MeSH]

OR "halcinonide" OR "halcinonide"[MeSH]

OR "halobetasol"

OR "halometasone"

OR "hydrocortamate"

OR "hydrocortisone" OR "hydrocortisone"[MeSH]

OR "loteprednol"

OR "meprednisone"

OR "methylprednisolone" OR "methylprednisolone"[MeSH]

OR "mometasone"

OR "paramethasone" OR "paramethasone"[MeSH]

OR "prednicarbate"

OR "prednisolone" OR "prednisolone"[MeSH]

OR "prednisone" OR "prednisone"[MeSH]

OR "rimexolone"

OR "tixocortol"

OR "triamcinolone" OR "triamcinolone"[MeSH]

) AND (

"humans"[mh]

AND English[lang]

AND ("2014/09/07"[PDat] : "2024/12/31"[PDat])

NOT "Comment" [pt]

NOT "Retracted Publication"[pt]

AND

(

"systematic review"[pt]

OR "systematic review"[ti]

OR "scoping review"[ti]

)

) AND (

"Glucocorticoids/adverse effects"[MeSH]

OR "Drug-Related Side Effects and Adverse Reactions"[Mesh]

OR "side effect*"[tiab]

OR "adverse effect*"[tiab]

OR "complication*"[tiab]

OR "toxicit*"[tiab]

OR "safety"[tiab]

OR "patient safety"[mh]

)

Data S3: Article screening tools

### Title and abstract screening tool

| The methods section indicates the study is a **systematic/scoping review** of quantitative study designs (experimental or observational, excluding case series or case reports) |
| --- |
| The results section of the abstract includes **evidence of oral glucocorticoid use** |
| Title and abstract **do not mention** that the participants in the systematic review are **children only** |
| Title and abstract **do not** **mention** that the participants in the systematic review are **pregnant women only** |

### Full-text eligibility criteria

| The results section of the full text includes evidence of use of **oral glucocorticoids** in the treatment of **at least 1 named chronic condition** |
| --- |
| The glucocorticoid use involves a **high dose**, a **prolonged duration,** and/or a **steroid-sparing strategy** |
| The results section of the full text includes safety outcomes of glucocorticoid(s). |

# Data S4: Data extraction tool

**Study ID**

**Title**

**Objective(s)**

Characteristics of included reviews

**Review type**

1. Systematic
2. Scoping
3. Other

**Table preview**

|  | **Number of included studies** |
| --- | --- |
| **RCT** |  |
| **Observational** |  |
| **Other** |  |

**Geographical location:** High income/ Middle income/ Low income/ Unspecified

**Care setting**

1. Primary
2. Secondary
3. Other

**Was ethnicity reported?** Yes / no

**Indication for glucocorticoids**

**Type(s) of glucocorticoids and pattern(s) of use:**

- Was there evidence of high doses?
- Was there evidence of long term use?
- Was there evidence of steroid sparing approaches?
- Was there evidence of tapering regimes?

**Notes**

Findings

**Adverse effects/toxicity profile (narrative summary)**

**Notes**

# Data S5: Excluded studies and reasons for exclusion

**Exclusion reason: Guideline (n=2)**

Palmowski Y; Buttgereit T; Dejaco C; Bijlsma JW; Matteson EL; Voshaar M; Boers M; Buttgereit F. 2017. "Official view" on glucocorticoids in rheumatoid arthritis: A systematic review of international guidelines and consensus statements. Arthritis Care Res (Hoboken), 69, 1134-1141.

De Souza AWS; Sato EI; Brance ML; Fernández-Ávila DG; Scolnik M; Magri SJ; Ugarte-Gil MF; Flores-Suárez LF; Saldarriaga-Rivera LM; Babini A; Zamora NV; Acosta Felquer ML; Vergara F; Carlevaris L; Scarafia S; Soriano Guppy ER; Unizony S. 2023. Pan American League of Associations for Rheumatology guidelines for the treatment of Takayasu arteritis. J Clin Rheumatol, 29, 316-325.

**Exclusion reason: Insufficient detail on glucocorticoid regimen (n=97)**

Liu B; Deng T; Zhang J. 2016. Risk Factors for Central Serous Chorioretinopathy: a systematic review and meta-analysis. Retina, 36, 43709.

Tan JP; Barazanchi AW; Singh PP; Hill AG; Maccormick AD. 2016. Predictors of acute diverticulitis severity: a systematic review. Int J Surg, 26, 43-52.

Casselman P; Cassiman C; Casteels I; Schauwvlieghe PP. 2021. Insights into multiple sclerosis-associated uveitis: A Scoping Review. Acta Ophthalmol, 99, 592-603.

Ang T; Chaggar V; Tong JY; Selva D. 2024. Medication-associated orbital inflammation: a systematic review. Surv Ophthalmol, 69, 622-631.

Muzzana M; Pedrazzoli P; Lasagna A. 2021. G-CSF and G-CSF-related vasculitis: a systematic review of the literature and intriguing future research perspectives. Future Oncol, 17, 4619-4634.

Lanzillotta M; Della-Torre E; Wallace ZS; Stone JH; Karadag O; Fernández-Codina A; Arcidiacono PG; Falconi M; Dagna L; Capurso G. 2021. Efficacy and safety of rituximab for IgG4-related pancreato-biliary disease: a systematic review and meta-analysis. Pancreatology, 21, 1395-1401.

Taylor J; Mcmillan R; Shephard M; Setterfield J; Ahmed R; Carrozzo M; Grando S; Mignogna M; Kuten-Shorrer M; Musbah T; Elia A; Mcgowan R; Kerr AR; Greenberg MS; Hodgson T; Sirois D. 2015. World Workshop on Oral Medicine VI: a systematic review of the treatment of mucous membrane pemphigoid. Oral Surg Oral Med Oral Pathol Oral Radiol, 120, 161-71.e20.

Tang Q; Chen Q; Li Y; Wang Z. 2022. Association between glucocorticoids and mortality in patients with severe pneumonia: a systematic review and meta-analysis based on randomized controlled trials. Comput Math Methods Med, 2022, 1191205.

Ma T; Wang Y; Ma J; Cui H; Feng X; Ma X. 2024. Research progress in the pathogenesis of hormone-induced femoral head necrosis based on microvessels: a systematic review. J Orthop Surg Res, 19, 265.

Pietris J; Lam A; Bacchi S; Gupta AK; Kovoor JG; Simon S; Slee M; Chan W. 2024. The Efficacy, Adverse Effects and Economic Implications of Oral Versus Intravenous Methylprednisolone for the Treatment of Optic Neuritis: A Systematic Review. Semin Ophthalmol, 39, 42522.

Stine JG; Chalasani N. 2015. Chronic liver injury induced by drugs: a systematic review. Liver Int, 35, 2343-53.

Santiago P; Schwartz I; Tamariz L; Levy C. 2019. Systematic review with meta-analysis: mycophenolate mofetil as a second-line therapy for autoimmune hepatitis. Aliment Pharmacol Ther, 49, 830-839.

Akiyama S; Hamdeh S; Micic D; Sakuraba A. 2021. Prevalence and clinical outcomes of COVID-19 in patients with autoimmune diseases: a systematic review and meta-analysis. Ann Rheum Dis, 80, 384-391.

Li H; Yang L; Song Y; Zhao X; Sun C; Zhang L; Zhao H; Pan Y. 2022. Comparative effectiveness of different treatment modalities for active, moderate-to-severe Graves' orbitopathy: a systematic review and network meta-analysis. Acta Ophthalmol, 100, e1189-e1198.

Ma C; Hussein IM; Al-Abbar YJ; Panaccione R; Fedorak RN; Parker CE; Nguyen TM; Khanna R; Siegel CA; Peyrin-Biroulet L; Pai RK; Vande Casteele N; D'Haens GR; Sandborn WJ; Feagan BG; Jairath V. 2018. Heterogeneity in Definitions of Efficacy and Safety Endpoints for Clinical Trials of Crohn's Disease: A Systematic Review. Clin Gastroenterol Hepatol, 16, 1407-1419.e22.

Lu W; Gong S; Li J; Luo H; Wang Y. 2020. Efficacy and safety of rituximab in the treatment of membranous nephropathy: A systematic review and meta-analysis. Medicine (Baltimore), 99, e19804.

Kolomeyer AM; Brucker AJ. 2018. PERSISTENT PLACOID MACULOPATHY: A Systematic Review. Retina, 38, 1881-1895.

Ruscitti P; McGonagle D; Garcia VC; Rabijns H; Toennessen K; Chappell M; Edwards M; Miller P; Hansell N; Moss J; Graziadio S; Feist E. 2024. Systematic Review and Metaanalysis of Pharmacological Interventions in Adult-Onset Still Disease and the Role of Biologic Disease-Modifying Antirheumatic Drugs. J Rheumatol, 51, 442-451.

Hernández-Rodríguez J; Carbonell C; Mirón-Canelo JA; Diez-Ruiz S; Marcos M; Chamorro AJ. 2020. Rituximab treatment for IgA vasculitis: A systematic review. Autoimmun Rev, 19, 102490.

Hoseinpour Jajarm H; Asadi R; Bardideh E; Shafaee H; Khazaei Y; Emadzadeh M. 2018. The effects of photodynamic and low-level laser therapy for treatment of oral lichen planus-a systematic review and meta-analysis. Photodiagnosis Photodyn Ther, 23, 254-260.

Houschyar KS; Tapking C; Borrelli MR; Puladi B; Ooms M; Wallner C; Duscher D; Pförringer D; Rein S; Reumuth G; Schulz T; Nietzschmann I; Maan ZN; Grieb G; Philipp-Dormston WG; Branski LK; Siemers F; Lehnhardt M; Schmitt L; Yazdi AS. 2021. Stevens-Johnson syndrome and toxic epidermal necrolysis: a systematic review and meta-analysis. J Wound Care, 30, 1012-1019.

Law CCY; Koh D; Bao Y; Jairath V; Narula N. 2020. Risk of Postoperative Infectious Complications From Medical Therapies in Inflammatory Bowel Disease: a systematic review and meta-analysis. Inflamm Bowel Dis, 26, 1796-1807.

D'Aguanno K; Gabrielli S; Ouchene L; Muntyanu A; Ben-Shoshan M; Zhang X; Iannattone L; Netchiporouk E. 2022. Omalizumab for the Treatment of Bullous Pemphigoid: A Systematic Review of Efficacy and Safety. J Cutan Med Surg, 26, 404-413.

Ala M; Ghasemi M; Mohammad Jafari R; Dehpour AR. 2021. Beyond its anti-migraine properties, sumatriptan is an anti-inflammatory agent: A systematic review. Drug Dev Res, 82, 896-906.

A Subahi E; Ata F; Choudry H; Iqbal P; A AlHiyari M; T Soliman A; De Sanctis V; A Yassin M. 2022. Extramedullary haematopoiesis in patients with transfusion dependent ß-thalassaemia (TDT): a systematic review. Ann Med, 54, 764-774.

Guo C; Wu K; Liang X; Liang Y; Li R. 2019. Infliximab clinically treating ulcerative colitis: a systematic review and meta-analysis. Pharmacol Res, 148, 104455.

Taneja V; El-Dallal M; Haq Z; Tripathi K; Systrom HK; Wang LF; Said H; Bain PA; Zhou Y; Feuerstein JD. 2022. Effectiveness and Safety of Tofacitinib for Ulcerative Colitis: Systematic Review and Meta-analysis. J Clin Gastroenterol, 56, e323-e333.

Bolkenstein HE; van de Wall BJM; Consten ECJ; Broeders IAMJ; Draaisma WA. 2017. Risk factors for complicated diverticulitis: systematic review and meta-analysis. Int J Colorectal Dis, 32, 1375-1383.

Kouverianos I; Angelopoulos A; Daoussis D. 2023. The role of anti-eosinophilic therapies in eosinophilic granulomatosis with polyangiitis: a systematic review. Rheumatol Int, 43, 1245-1252.

Borrirukwisitsak S; Tantayakom P; Katchamart W. 2021. Efficacy and safety of rituximab on lung and skin involvement in systemic sclerosis: a systematic review and metaanalysis. Clin Rheumatol, 40, 2779-2789.

Delpachitra MR; Heal C; Banks J; Divakaran P; Pawar M. 2019. Risk Factors for Surgical Site Infection in Minor Dermatological Surgery: A Systematic Review. Adv Skin Wound Care, 32, 217-226.

Godfrey KJ; Kazim M. 2018. Radiotherapy for Active Thyroid Eye Disease. Ophthalmic Plast Reconstr Surg, 34, S98-S104.

Chu AWL; Wong MM; Rayner DG; Guyatt GH; Díaz Martinez JP; Ceccacci R; Zhao IX; McMullen E; Srivastava A; Wang J; Wen A; Wang FC; Brignardello-Petersen R; Izcovich A; Oykhman P; Wheeler KE; Spergel JM; Singh JA; Silverberg JI; Ong PY; O'Brien M; Martin SA; Lio PA; Lind ML; LeBovidge J; Kim E; Huynh J; Greenhawt M; Gardner DD; Frazier WT; Ellison K; Chen L; Capozza K; De Benedetto A; Boguniewicz M; Smith Begolka W; Asiniwasis RN; Schneider LC; Chu DK. 2023. Systemic treatments for atopic dermatitis (eczema): Systematic review and network meta-analysis of randomized trials. J Allergy Clin Immunol, 152, 1470-1492.

Ge Y; Peng Q; Zhang S; Zhou H; Lu X; Wang G. 2015. Cyclophosphamide treatment for idiopathic inflammatory myopathies and related interstitial lung disease: a systematic review. Clin Rheumatol, 34, 99-105.

Liu X; Gao C; Gao T. 2019. Efficacy and safety of tripterygium glycosides for Graves ophthalmopathy: a systematic review and meta-analysis. Medicine (Baltimore), 98, e18242.

Taxonera C; Olivares D; Alba C. 2022. Real-World Effectiveness and Safety of Tofacitinib in Patients With Ulcerative Colitis: Systematic Review With Meta-Analysis. Inflamm Bowel Dis, 28, 32-40.

Struja T; Guebelin L; Kutz A; Fehlberg H; Mueller B; Schuetz P. 2016. Does Immunosuppressive Therapy Improve Outcomes in Graves' Disease? A systematic review and meta-analysis. Thyroid, 26, 634-40.

Kyriakopoulos C; Gogali A; Markozannes G; Kostikas K. 2024. Biologic agents licensed for severe asthma: a systematic review and meta-analysis of randomised controlled trials. Eur Respir Rev, 33, .

Ma C; Panaccione R; Fedorak RN; Parker CE; Nguyen TM; Khanna R; Siegel CA; Peyrin-Biroulet L; D'Haens G; Sandborn WJ; Feagan BG; Jairath V. 2018. Heterogeneity in Definitions of Endpoints for Clinical Trials of Ulcerative Colitis: A Systematic Review for Development of a Core Outcome Set. Clin Gastroenterol Hepatol, 16, 637-647.e13.

Liu B; Meng X; Ma Y; Li H; Liu Y; Shi N; Chen Y; Wang Y; Lu C. 2021. Clinical safety of total glucosides of paeony adjuvant therapy for rheumatoid arthritis treatment: a systematic review and meta-analysis. BMC Complement Med Ther, 21, 102.

Albuquerque JV; Andriolo BN; Vasconcellos MR; Civile VT; Lyddiatt A; Trevisani VF. 2019. Interventions for morphea. Cochrane Database Syst Rev, 7, CD005027.

Huang Y; Wang H; Wan L; Lu X; Tam WWS. 2016. Is Systemic Lupus Erythematosus Associated With a Declined Immunogenicity and Poor Safety of Influenza Vaccination?: A systematic review and meta-analysis. Medicine (Baltimore), 95, e3637.

Petit C; Culshaw S; Weiger R; Huck O; Sahrmann P. 2024. Impact of treatment of rheumatoid arthritis on periodontal disease: A review. Mol Oral Microbiol, 39, 199-224.

Cota-Arce JM; Cota J; De León-Nava MA; Hernández-Cáceres A; Moncayo-Salazar LI; Valle-Alvarado F; Cordero-Moreno VL; Bonfil-Solis KL; Bichara-Figueroa JE; Hernández-Hernández J; Villela L. 2021. Efficacy and safety of canakinumab in the treatment of adult-onset Still's disease: A systematic review. Semin Arthritis Rheum, 51, 1282-1290.

Dahiya DS; Chandan S; Bapaye J; Mohan BP; Ramai D; Kassab LL; Chandan OC; Dulai PS; Kochhar GS. 2024. Safety and Effectiveness of Vedolizumab in Elderly Patients with Inflammatory Bowel Disease: A Systematic Review & Meta-Analysis. J Clin Gastroenterol, 58, 378-388.

Wong ZY; Teo CY; Fiona Wong YQ; Ng KT; Lim SK. 2024. Immunosuppression for adult steroid-dependent or frequently relapsing nephrotic syndrome: a systematic review and meta-analysis. PLoS One, 19, e0307981.

Qiu B; Liang JX; Li C. 2022. Efficacy and safety of vedolizumab for inflammatory bowel diseases: a systematic review and meta-analysis of randomized controlled trials. Medicine (Baltimore), 101, e30590.

Sarmadian R; Safi F; Sarmadian H; Shokrpour M; Almasi-Hashiani A. 2024. Treatment modalities for granulomatous mastitis, seeking the most appropriate treatment with the least recurrence rate: a systematic review and meta-analysis. Eur J Med Res, 29, 164.

Wang J; Tan L; Wang HF; Tan CC; Meng XF; Wang C; Tang SW; Yu JT. 2015. Anti-inflammatory drugs and risk of Alzheimer's disease: an updated systematic review and meta-analysis. J Alzheimers Dis, 44, 385-96.

Dalal DS; Duran J; Brar T; Alqadi R; Halladay C; Lakhani A; Rudolph JL. 2019. Efficacy and safety of biological agents in the older rheumatoid arthritis patients compared to Young: a systematic review and meta-analysis. Semin Arthritis Rheum, 48, 799-807.

Alrashed F; Battat R; Abdullah I; Charabaty A; Shehab M. 2021. Impact of medical therapies for inflammatory bowel disease on the severity of COVID-19: a systematic review and meta-analysis. BMJ Open Gastroenterol, 8

Sheehan JL; Jacob J; Berinstein EM; Greene-Higgs L; Steiner CA; Berry SK; Shannon C; Cohen-Mekelburg SA; Higgins PDR; Berinstein JA. 2022. The Relationship Between Opioid Use and Healthcare Utilization in Patients With Inflammatory Bowel Disease: a systematic review and meta-analysis. Inflamm Bowel Dis, 28, 1904-1914.

Luan ZJ; Li Y; Zhao XY; Wang L; Sun YH; Wang SY; Qian JM. 2016. Treatment efficacy and safety of low-dose azathioprine in chronic active ulcerative colitis patients: A meta-analysis and systemic review. J Dig Dis, 17, 652-659.

Wu Q; Yuan L; Qiu H; Wang X; Huang X; Zheng R; Yang Q. 2021. Efficacy and safety of omalizumab in chronic rhinosinusitis with nasal polyps: a systematic review and meta-analysis of randomised controlled trials. BMJ Open, 11, e047344.

Rozich JJ; Dulai PS; Fumery M; Sandborn WJ; Singh S. 2020. Progression of Elderly Onset Inflammatory Bowel Diseases: a systematic review and meta-analysis of Population-Based Cohort Studies. Clin Gastroenterol Hepatol, 18, 2437-2447.e6.

El Hage Chehade N; Ghoneim S; Shah S; Pardi DS; Farraye FA; Francis FF; Hashash JG. 2024. Efficacy and Safety of Vedolizumab and Tumor Necrosis Factor Inhibitors in the Treatment of Steroid-refractory Microscopic Colitis: a systematic review and meta-analysis. J Clin Gastroenterol, 58, 789-799.

Wang HF; Wang YY; Li ZY; He PJ; Liu S; Li QS. 2024. The prevalence and risk factors of rheumatoid arthritis-associated interstitial lung disease: a systematic review and meta-analysis. Ann Med, 56, 2332406.

Ling RR; Ramanathan K; Sim JJL; Wong SN; Chen Y; Amin F; Fernando SM; Rochwerg B; Fan E; Barbaro RP; MacLaren G; Shekar K; Brodie D. 2022. Evolving outcomes of extracorporeal membrane oxygenation during the first 2 years of the COVID-19 pandemic: a systematic review and meta-analysis. Crit Care, 26, 147.

Tsai L; Nguyen NH; Ma C; Prokop LJ; Sandborn WJ; Singh S. 2022. Systematic Review and Meta-Analysis: Risk of Hospitalization in Patients with Ulcerative Colitis and Crohn's Disease in Population-Based Cohort Studies. Dig Dis Sci, 67, 2451-2461.

Yan R; Jin YB; Li XR; Luo L; Liu XM; He J. 2022. Clinical characteristics of rheumatic disease-associated hypophysitis: A case series and review of literature. Medicine (Baltimore), 101, e31338.

Delplanque M; Pouchot J; Ducharme-Bénard S; Fautrel BJ; Benyamine A; Daniel L; Gendre T; Grateau G; Georgin-Lavialle S. 2020. AA amyloidosis secondary to adult onset Still's disease: About 19 cases. Semin Arthritis Rheum, 50, 156-165.

Sarlos P; Szemes K; Hegyi P; Garami A; Szabo I; Illes A; Solymar M; Petervari E; Vincze A; Par G; Bajor J; Czimmer J; Huszar O; Varju P; Farkas N. 2018. Steroid but not Biological Therapy Elevates the risk of Venous Thromboembolic Events in Inflammatory Bowel Disease: A Meta-Analysis. J Crohns Colitis, 12, 489-498.

Macaluso FS; Maida M; Ventimiglia M; Orlando A. 2022. Effectiveness and safety of tofacitinib for the treatment of ulcerative colitis: A single-arm meta-analysis of observational studies. Dig Liver Dis, 54, 183-191.

Farne HA; Wilson A; Milan S; Banchoff E; Yang F; Powell CV. 2022. Anti-IL-5 therapies for asthma. Cochrane Database Syst Rev, 7, CD010834.

Garegnani L; Hyland M; Roson Rodriguez P; Escobar Liquitay CM; Franco JV. 2021. Antioxidants to prevent respiratory decline in people with Duchenne muscular dystrophy and progressive respiratory decline. Cochrane Database Syst Rev, 12, CD013720.

Li Z; Xu D; Wang Z; Wang Y; Zhang S; Li M; Zeng X. 2017. Gastrointestinal system involvement in systemic lupus erythematosus. Lupus, 26, 1127-1138.

Benstead TJ; Chalk CH; Parks NE. 2014. Treatment for cryoglobulinemic and non-cryoglobulinemic peripheral neuropathy associated with hepatitis C virus infection. Cochrane Database Syst Rev, 2014, CD010404.

Stork AC; Lunn MP; Nobile-Orazio E; Notermans NC. 2015. Treatment for IgG and IgA paraproteinaemic neuropathy. Cochrane Database Syst Rev, 2015, CD005376.

Rungjirajittranon T; Suwanawiboon B; Nakkinkun Y; Leelakanok N; Kaokunakorn T; Chinthammitr Y; Owattanapanich W; Ruchutrakool T. 2024. First-line immunosuppressive therapies for acquired hemophilia A: A 25-year cohort experience and network meta-analysis. Thromb Res, 241, 109067.

Kerschbaumer A; Smolen JS; Ferreira RJO; Bertheussen H; Baraliakos X; Aletaha D; McGonagle DG; van der Heijde D; McInnes IB; Esbensen BA; Winthrop KL; Boehncke WH; Schoones JW; Gossec L. 2024. Efficacy and safety of pharmacological treatment of psoriatic arthritis: a systematic literature research informing the 2023 update of the EULAR recommendations for the management of psoriatic arthritis. Ann Rheum Dis, 83, 760-774.

Barry RJ; Tallouzi MO; Bucknall N; Mathers JM; Murray PI; Calvert MJ; Moore DJ; Denniston AK. 2018. Anti-tumour necrosis factor biological therapies for the treatment of uveitic macular oedema (UMO) for non-infectious uveitis. Cochrane Database Syst Rev, 12, CD012577.

Wei D; Chen Y; Shen Y; Xie B; Song X. 2023. Efficacy and safety of different JAK inhibitors in the treatment of alopecia areata: a network meta-analysis. Front Immunol, 14, 1152513.

Sukhanova AM; Gilavian MA; Melnik EV; Shikh EV; Petukhov AE; Gegechkori VI; Dementev SP; Vlasov AM; Ramenskaya GV. 2024. An Overview of Adalimumab Therapy for Ankylosing Spondylitis. Curr Rheumatol Rev, 20, 501-513.

Kaur L; Gordon M; Baines PA; Iheozor-Ejiofor Z; Sinopoulou V; Akobeng AK. 2020. Probiotics for induction of remission in ulcerative colitis. Cochrane Database Syst Rev, 3, CD005573.

Xiong Y; Cuevas S; Xu G; Zou H. 2024. The efficacy of rituximab in the treatment of IgA vasculitis nephritis. Clin Exp Med, 24, 213.

Xavier NF; Lucena DT; Cruz AAV. 2023. Monoclonal Antibodies for the Treatment of Graves Orbitopathy: Precision Medicine? Ophthalmic Plast Reconstr Surg, 39, 307-315.

Rai A; Shrivastava PK; Kumar A; Prasad K; Shakeel S; Ul Haque Z. 2023. Comparative effectiveness of medicinal interventions for oral submucous fibrosis: A network meta-analysis. J Stomatol Oral Maxillofac Surg, 124, 101423.

Bixio R; Bertelle D; Pistillo F; Pedrollo E; Carletto A; Rossini M; Viapiana O. 2022. Rheumatoid arthritis and myasthenia gravis: a case-based review of the therapeutic options. Clin Rheumatol, 41, 1247-1254.

Ridley B; Minozzi S; Gonzalez-Lorenzo M; Del Giovane C; Piggott T; Filippini G; Peryer G; Foschi M; Tramacere I; Baldin E; Nonino F. 2024. Immunomodulators and immunosuppressants for progressive multiple sclerosis: a network meta-analysis. Cochrane Database Syst Rev, 9, CD015443.

Rice JB; White AG; Scarpati LM; Wan G; Nelson WW. 2017. Long-term Systemic Corticosteroid Exposure: A Systematic Literature Review. Clin Ther, 39, 2216-2229.

Kilian A; Chock YP; Huang IJ; Graef ER; Upton LA; Khilnani A; Krupnikova SDS; Almaghlouth I; Cappelli LC; Fernandez-Ruiz R; Frankel BA; Frankovich J; Harrison C; Kumar B; Monga K; Vega JAR; Singh N; Sparks JA; Sullo E; Young KJ; Duarte-Garcia A; Putman M; Johnson S; Grainger R; Wallace ZS; Liew JW; Jayatilleke A. 2020. Acute respiratory viral adverse events during use of antirheumatic disease therapies: A scoping review. Semin Arthritis Rheum, 50, 1191-1201.

Sener H; Evereklioglu C; Horozoglu F; Gunay Sener AB. 2024. Efficacy and Safety of Adalimumab in Patients with Behçet Uveitis: a systematic review and meta-analysis. Ocul Immunol Inflamm, 32, 89-97.

Mullol J; Maldonado M; Castillo JA; Miguel-Blanco C; Dávila I; Domínguez-Ortega J; Blanco-Aparicio M. 2022. Management of United Airway Disease Focused on Patients With Asthma and Chronic Rhinosinusitis With Nasal Polyps: A Systematic Review. J Allergy Clin Immunol Pract, 10, 2438-2447.e9.

Fukuda Y; Horita N; Aga M; Kashizaki F; Hara Y; Obase Y; Niimi A; Kaneko T; Mukae H; Sagara H. 2024. Efficacy and safety of macrolide therapy for adult asthma: a systematic review and meta-analysis. Respir Investig, 62, 206-215.

Pacheco RL; Latorraca COC; de Souza AWS; Pachito DV; Riera R. 2017. Clinical interventions for Takayasu arteritis: a systematic review. Int J Clin Pract, 71, .

Agache I; Beltran J; Akdis C; Akdis M; Canelo-Aybar C; Canonica GW; Casale T; Chivato T; Corren J; Del Giacco S; Eiwegger T; Firinu D; Gern JE; Hamelmann E; Hanania N; Mäkelä M; Hernández-Martín I; Nair P; O'Mahony L; Papadopoulos NG; Papi A; Park HS; Pérez de Llano L; Posso M; Rocha C; Quirce S; Sastre J; Shamji M; Song Y; Steiner C; Schwarze J; Alonso-Coello P; Palomares O; Jutel M. 2020. Efficacy and safety of treatment with biologicals (benralizumab, dupilumab, mepolizumab, omalizumab and reslizumab) for severe eosinophilic asthma. A systematic review for the EAACI Guidelines - recommendations on the use of biologicals in severe asthma. Allergy, 75, 1023-1042.

Chen T; Ashman PE; Bojrab DI 2nd; Johnson AP; Hong RS; Benson B; Svider PF. 2021. Diagnosis and management of eosinophilic otitis media: a systematic review. Acta Otolaryngol, 141, 579-587.

Chen S; Zhou A; Emmanuel B; Thomas K; Guiang H. 2020. Systematic literature review of the epidemiology and clinical burden of chronic rhinosinusitis with nasal polyposis. Curr Med Res Opin, 36, 1897-1911.

Hussein S; Suitner M; Béland-Bonenfant S; Baril-Dionne A; Vandermeer B; Santesso N; Keeling S; Pope JE; Fifi-Mah A; Bourré-Tessier J. 2018. Monitoring of Osteonecrosis in Systemic Lupus Erythematosus: A Systematic Review and Metaanalysis. J Rheumatol, 45, 1462-1476.

Law CC; Bell C; Koh D; Bao Y; Jairath V; Narula N. 2020. Risk of postoperative infectious complications from medical therapies in inflammatory bowel disease. Cochrane Database Syst Rev, 10, CD013256.

Jiang N; Jin S; Yu C; Zhao J; Wang Q; Tian X; Li M; Zeng X. 2023. Efficacy and safety of immunosuppressive agents for adults with lupus nephritis: a systematic review and network meta-analysis. Front Immunol, 14, 1232244.

Safy M; de Hair MJH; Jacobs JWG; Buttgereit F; Kraan MC; van Laar JM. 2017. Efficacy and safety of selective glucocorticoid receptor modulators in comparison to glucocorticoids in arthritis, a systematic review. PLoS One, 12, e0188810.

Salman S; Awad M; Sarsik S; Ibrahim AM; Fathi M; Agha NY; Anis R; El Ashal G; Salem ML. 2020. Treatment options for autoimmune bullous dermatoses other than systemic steroids: A systematic review and network meta-analysis. Dermatol Ther, 33, e13861.

Tsai TY; Huang IH; Chao YC; Li H; Hsieh TS; Wang HH; Huang YT; Chen CY; Cheng YC; Kuo PH; Huang YC; Tu YK. 2021. Treating toxic epidermal necrolysis with systemic immunomodulating therapies: A systematic review and network meta-analysis. J Am Acad Dermatol, 84, 390-397.

Torres-Navarro I; Briz-Redón Á; Botella-Estrada R. 2021. Systemic therapies for Stevens-Johnson Syndrome and Toxic Epidermal Necrolysis: a SCORTEN-based systematic review and meta-analysis. J Eur Acad Dermatol Venereol, 35, 159-171.

Nagasaka K; Harigai M; Hagino N; Hara A; Horita T; Hayashi T; Itabashi M; Ito S; Katsumata Y; Kawashima S; Naniwa T; Sada KE; Nango E; Nakayama T; Tsutsumino M; Yamagata K; Homma S; Arimura Y. 2019. Systematic review and meta-analysis for 2017 clinical practice guidelines of the Japan research committee of the ministry of health, labour, and welfare for intractable vasculitis for the management of ANCA-associated vasculitis. Mod Rheumatol, 29, 119-129.

Doukaki S; Platamone A; Alaimo R; Bongiorno MR. 2015. Mycophenolate mofetil and enteric-coated mycophenolate sodium in the treatment of pemphigus vulgaris and pemphigus foliaceus. J Dermatolog Treat, 26, 67-72.

**Exclusion reason: Insufficient glucocorticoid dose/duration (n=24)**

Nielsen AW; Hemmig AK; de Thurah A; Schmidt WA; Sattui SE; Mackie SL; Brouwer E; Dejaco C; Keller KK; Mukhtyar CB. 2023. Early referral of patients with suspected polymyalgia rheumatica - A systematic review. Semin Arthritis Rheum, 63, 152260.

Canellas JVDS; Ritto FG; Tiwana P. 2022. Comparative efficacy and safety of different corticosteroids to reduce inflammatory complications after mandibular third molar surgery: a systematic review and network meta-analysis. Br J Oral Maxillofac Surg, 60, 1035-1043.

Gérard AL; Simon-Tillaux N; Yordanov Y; Cacoub P; Tubach F; Saadoun D; Dechartres A. 2021. Efficacy and safety of steroid-sparing treatments in giant cell arteritis according to the glucocorticoids tapering regimen: a systematic review and meta-analysis. Eur J Intern Med, 88, 96-103.

Tatalovic M; Lehmann R; Cheetham M; Nowak A; Battegay E; Rampini SK. 2019. Management of hyperglycaemia in persons with non-insulin-dependent type 2 diabetes mellitus who are started on systemic glucocorticoid therapy: a systematic review. BMJ Open, 9, e028914.

Shung DL; Abraham B; Sellin J; Hou JK. 2015. Medical and surgical complications of inflammatory bowel disease in the elderly: a systematic review. Dig Dis Sci, 60, 1132-40.

Larivée N; Chin CJ. 2020. Aspirin desensitization therapy in aspirin-exacerbated respiratory disease: a systematic review. Int Forum Allergy Rhinol, 10, 450-464.

Mortensen SJ; Mohamadi A; Wright CL; Chan JJ; Weaver MJ; von Keudell A; Nazarian A. 2020. Medications as a Risk Factor for Fragility Hip Fractures: a systematic review and meta-analysis. Calcif Tissue Int, 107, 45901.

Sales GMPG; Barbosa ICP; Canejo Neta LMS; Melo PL; Leitão RA; Melo HMA. 2018. Treatment of chikungunya chronic arthritis: A systematic review. Rev Assoc Med Bras (1992), 64, 63-70.

Wang X; Wang P; Faramand A; Zha X; Zhang Y; Chong W; Hai Y; Fang F. 2022. Efficacy and safety of corticosteroid in the treatment of hand osteoarthritis: a systematic review and meta-analysis of randomized controlled trials. Clin Rheumatol, 41, 1825-1832.

Székely H; Tóth LM; Rancz A; Walter A; Farkas N; Sárközi MD; Váncsa S; Eross B; Hegyi P; Miheller P. 2024. Anti-tumor Necrosis Factor Alpha Versus Corticosteroids: A 3-fold Difference in the Occurrence of Venous Thromboembolism in Inflammatory Bowel Disease-a systematic review and meta-analysis. J Crohns Colitis, 18, 773-783.

Fautrel B; Patterson J; Bowe C; Arber M; Glanville J; Mealing S; Canon-Garcia V; Fagerhed L; Rabijns H; Giacomelli R. 2023. Systematic review on the use of biologics in adult-onset still's disease. Semin Arthritis Rheum, 58, 152139.

Both T; Dalm VASH; Richardson SA; van Schie N; van den Broek LM; de Vries AC; van Hagen PM; Rombach SM. 2021. Inflammatory bowel disease in primary immunodeficiency disorders is a heterogeneous clinical entity requiring an individualized treatment strategy: A systematic review. Autoimmun Rev, 20, 102872.

Migliorini F; Maffulli N; Shukla T; D'Ambrosi R; Singla M; Vaish A; Vaishya R. 2024. The pandemic is gone but its consequences are here to stay: avascular necrosis following corticosteroids administration for severe COVID-19. J Orthop Surg Res, 19, 135.

Köhler-Forsberg O; N Lydholm C; Hjorthøj C; Nordentoft M; Mors O; Benros ME. 2019. Efficacy of anti-inflammatory treatment on major depressive disorder or depressive symptoms: meta-analysis of clinical trials. Acta Psychiatr Scand, 139, 404-419.

Liu L; Jing FY; Wang XW; Li LJ; Zhou RQ; Zhang C; Wu QC. 2021. Effects of corticosteroids on new-onset atrial fibrillation after cardiac surgery: A meta-analysis of randomized controlled trials. Medicine (Baltimore), 100, e25130.

Xie H; Guo J; Tan B; Wu H. 2019. Efficacy of Salvia miltiorrhiza injection combined with steroids in the treatment of oral submucous fibrosis: A meta-analysis of randomized controlled trials. Medicine (Baltimore), 98, e16339.

Nankervis H; Thomas KS; Delamere FM; Barbarot S; Smith S; Rogers NK; Williams HC. 2017. What is the evidence base for atopic eczema treatments? A summary of published randomized controlled trials. Br J Dermatol, 176, 910-927.

Joudeh AI; Lutf AQ; Mahdi S; Tran G. 2023. Efficacy and safety of mRNA and AstraZeneca COVID-19 vaccines in patients with autoimmune rheumatic diseases: A systematic review. Vaccine, 41, 3801-3812.

Jin XX; Wang X; Shan Y; Li SZ; Xu Q; Jin HZ; Zuo YG. 2022. Efficacy and safety of tetracyclines for pemphigoid: a systematic review and meta-analysis. Arch Dermatol Res, 314, 191-201.

Chu X; Wang J; Ologundudu L; Brignardello-Petersen R; Guyatt GH; Oykhman P; Bernstein JA; Saini SS; Beck LA; Waserman S; Moellman J; Khan DA; Ben-Shoshan M; Baker DR; Oliver ET; Sheikh J; Lang D; Mathur SK; Winders T; Eftekhari S; Gardner DD; Runyon L; Asiniwasis RN; Cole EF; Chan J; Wheeler KE; Trayes KP; Tran P; Chu DK. 2024. Efficacy and Safety of Systemic Corticosteroids for Urticaria: a systematic review and meta-analysis of Randomized Clinical Trials. J Allergy Clin Immunol Pract, 12, 1879-1889.e8.

Bonovas S; Nikolopoulos GK; Piovani D; González-Lorenzo M; Pantavou K; Lytras T; Peyrin-Biroulet L; Danese S. 2019. Comparative assessment of budesonide-MMX and mesalamine in active, mild-to-moderate ulcerative colitis: A systematic review and network meta-analysis. Br J Clin Pharmacol, 85, 2244-2254.

Chande N; Al Yatama N; Bhanji T; Nguyen TM; McDonald JW; MacDonald JK. 2017. Interventions for treating lymphocytic colitis. Cochrane Database Syst Rev, 7, CD006096.

Tomizawa Y; Melek J; Komaki Y; Kavitt RT; Sakuraba A. 2018. Efficacy of Pharmacologic Therapy for Eosinophilic Esophagitis: A Systematic Review and Network Meta-Analysis. J Clin Gastroenterol, 52, 596-606.

Yi J; He Z; Xu S; Feng S. 2019. Efficacy and safety of leflunomide in IgA nephropathy: a systematic review and meta-analysis. Int Urol Nephrol, 51, 1987-1998.

**Exclusion reason: Literature review (n=3)**

Howell AN; Ghamrawi RI; Strowd LC; Feldman SR. 2020. Pharmacological management of atopic dermatitis in the elderly. Expert Opin Pharmacother, 21, 761-771.

Mitrovic S; Fautrel B. 2018. Complications of adult-onset Still's disease and their management. Expert Rev Clin Immunol, 14, 351-365.

Stine JG; Lewis JH. 2016. Current and future directions in the treatment and prevention of drug-induced liver injury: a systematic review. Expert Rev Gastroenterol Hepatol, 10, 517-36.

**Exclusion reason: No additional data/empty review (n=9)**

Hembree AE; Scherl E. 2022. Diagnosis and Management of Cuffitis: A Systematic Review. Dis Colon Rectum, 65, S85-S91.

Jat KR; Walia DK; Khairwa A. 2021. Anti-IgE therapy for allergic bronchopulmonary aspergillosis in people with cystic fibrosis. Cochrane Database Syst Rev, 9, CD010288.

Remmington T; Smith S. 2021. Rituximab for eradicating inhibitors in people with acquired haemophilia A. Cochrane Database Syst Rev, 8, CD011907.

Yu SH; Drucker AM; Lebwohl M; Silverberg JI. 2018. A systematic review of the safety and efficacy of systemic corticosteroids in atopic dermatitis. J Am Acad Dermatol, 78, 733-740.e11.

Hughes RA; Mehndiratta MM. 2015. Corticosteroids for chronic inflammatory demyelinating polyradiculoneuropathy. Cochrane Database Syst Rev, 1, CD002062.

Kew KM; Undela K; Kotortsi I; Ferrara G. 2015. Macrolides for chronic asthma. Cochrane Database Syst Rev, CD002997.

Chen Y; Schieppati A; Chen X; Cai G; Zamora J; Giuliano GA; Braun N; Perna A. 2014. Immunosuppressive treatment for idiopathic membranous nephropathy in adults with nephrotic syndrome. Cochrane Database Syst Rev, 2014, CD004293.

Xiao Y; Guyatt G; Zeng L; Rw Jayne D; A Merkel P; Ac Siemieniuk R; Dookie JE; A Buchan T; Ahmed MM; J Couban R; Mahr A; Walsh M. 2022. Comparative efficacy and safety of alternative glucocorticoids regimens in patients with ANCA-associated vasculitis: a systematic review. BMJ Open, 12, e050507.

Antonio AA; Santos RN; Abariga SA. 2021. Tocilizumab for giant cell arteritis. Cochrane Database Syst Rev, 8, CD013484.

**Exclusion reason: No indication for glucocorticoids (n=4)**

Mortimer KJ; Cruz AA; Sepúlveda-Pachón IT; Jorga A; Vroling H; Williams C. 2024. Global herpes zoster burden in adults with asthma: a systematic review and meta-analysis. Eur Respir J, 64.

Ramiro S; Smolen JS; Landewé R; van der Heijde D; Dougados M; Emery P; de Wit M; Cutolo M; Oliver S; Gossec L. 2016. Pharmacological treatment of psoriatic arthritis: a systematic literature review for the 2015 update of the EULAR recommendations for the management of psoriatic arthritis. Ann Rheum Dis, 75, 490-8.

You L; Ye P; Xiao G; Liang J; Kong Y. 2021. Rituximab for the treatment of idiopathic membranous nephropathy with nephrotic syndrome: a systematic review and meta-analysis. Turk J Med Sci, 51, 2870-2880.

Broderick C; Kobayashi S; Suto M; Ito S; Kobayashi T. 2023. Intravenous immunoglobulin for the treatment of Kawasaki disease. Cochrane Database Syst Rev, 1, CD014884.

**Exclusion reason: Not English language (n=1)**

Yingyan Z; Huasheng L; Jingyao Y; Xiaohong HE; Lili P; Xue LI; Xianghong C; Xiumin C; Aicheng Y; Qingchun H. 2022. Effectiveness and safety of tripterygium glycosides tablet for lupus nephritis: a systematic review and Meta-analysis. J Tradit Chin Med, 42, 671-680.

**Exclusion reason: Protocol only (n=3)**

Ding GR; Ni JM; Zhang SJ; Xie YZ; Feng JF. 2019. Efficacy of methylprednisolone for treatment of persistent vertigo. Medicine (Baltimore), 98, e17194.

Li J; Zhang J; Jiang L; Li Z; Li F; Chen H; Feng L. 2019. Efficacy and safety of ultrasound-guided intrathyroidal injection of glucocorticoids versus routine oral administration of glucocorticoids for subacute thyroiditis: Protocol of systematic review and meta-analysis. Medicine (Baltimore), 98, e18564.

Wu D; Yang Z; Zhao C; Yao L. 2018. Infliximab versus cyclosporine for severe ulcerative colitis refractory to steroids: A protocol for systematic review and meta-analysis. Medicine (Baltimore), 97, e12657.

**Exclusion reason: Rescue use only (n=9)**

Walters JA; Tan DJ; White CJ; Wood-Baker R. 2018. Different durations of corticosteroid therapy for exacerbations of chronic obstructive pulmonary disease. Cochrane Database Syst Rev, 3, CD006897.

Awad A; Goh MS; Trubiano JA. 2023. Drug Reaction With Eosinophilia and Systemic Symptoms: A Systematic Review. J Allergy Clin Immunol Pract, 11, 1856-1868.

Evans DJ; Kew KM; Anderson DE; Boyter AC. 2015. Long-acting muscarinic antagonists (LAMA) added to inhaled corticosteroids (ICS) versus higher dose ICS for adults with asthma. Cochrane Database Syst Rev, 2015, CD011437.

Zhao Z; Lou O; Wang Y; Yin R; Gong C; Deng F; Wu EC; Xie JY; Wu J; Ma A; Guo Y; Xiong WT. 2023. Long- versus short-duration systemic corticosteroid regimens for acute exacerbations of COPD: a systematic review and meta-analysis of randomized trials and cohort studies. PLoS One, 18, e0296470.

Yang D; Guo X; Liu T; Li Y; Du Z; Liu C. 2021. Efficacy and Safety of Prostaglandin D2 Receptor 2 Antagonism with Fevipiprant for Patients with Asthma: a systematic review and meta-analysis of Randomized Controlled Trials. Curr Allergy Asthma Rep, 21, 39.

Kew KM; Dahri K. 2016. Long-acting muscarinic antagonists (LAMA) added to combination long-acting beta2-agonists and inhaled corticosteroids (LABA/ICS) versus LABA/ICS for adults with asthma. Cochrane Database Syst Rev, 2016, CD011721.

Kew KM; Evans DJ; Allison DE; Boyter AC. 2015. Long-acting muscarinic antagonists (LAMA) added to inhaled corticosteroids (ICS) versus addition of long-acting beta2-agonists (LABA) for adults with asthma. Cochrane Database Syst Rev, 2015, CD011438.

Williamson A; Martineau AR; Sheikh A; Jolliffe D; Griffiths CJ. 2023. Vitamin D for the management of asthma. Cochrane Database Syst Rev, 2, CD011511.

Normansell R; Kew KM; Mathioudakis AG. 2017. Interventions to improve inhaler technique for people with asthma. Cochrane Database Syst Rev, 3, CD012286.

**Exclusion reason: Unable to access (n=3)**

Nguyen VQ; Ulrik CS. 2016. Measures to reduce maintenance therapy with oral corticosteroid in adults with severe asthma. Allergy Asthma Proc, 37, 125-139.

Fleischmann R; Furst DE. 2020. Safety of repository corticotropin injection as an adjunctive therapy for the treatment of rheumatoid arthritis. Expert Opin Drug Saf, 19, 935-944.

Avondo S; Andreis A; Casula M; Biondi-Zoccai G; Imazio M. 2021. Pharmacologic treatment of acute and recurrent pericarditis: a systematic review and meta-analysis of controlled clinical trials. Panminerva Med, 63, 314-323.

**Exclusion reason: Wrong comparator (n=16)**

Xiang Q; Yang M; Luo W; Cao Y; Shuai S; Wei X; Xiong A. 2024. Combined glucocorticoids and cyclophosphamide in the treatment of Graves' ophthalmopathy: a systematic review and meta-analysis. BMC Endocr Disord, 24, 12.

de Cerqueira DPA; Pedreira ALS; de Cerqueira MG; Santiago MB. 2021. Biological therapy in rheumatoid vasculitis: a systematic review. Clin Rheumatol, 40, 1717-1724.

Wang L; Zhang S; Xi J; Li W; Zhou L; Lu J; Zhang T; Zhao C. 2017. Efficacy and safety of tacrolimus for myasthenia gravis: a systematic review and meta-analysis. J Neurol, 264, 2191-2200.

Kerschbaumer A; Sepriano A; Bergstra SA; Smolen JS; van der Heijde D; Caporali R; Edwards CJ; Verschueren P; de Souza S; Pope JE; Takeuchi T; Hyrich KL; Winthrop KL; Aletaha D; Stamm TA; Schoones JW; Landewé RBM. 2023. Efficacy of synthetic and biological DMARDs: a systematic literature review informing the 2022 update of the EULAR recommendations for the management of rheumatoid arthritis. Ann Rheum Dis, 82, 95-106.

Nielsen DL; Juhl CB; Chen IM; Kellermann L; Nielsen OH. 2022. Immune checkpoint Inhibitor-Induced diarrhea and Colitis: Incidence and Management. a systematic review and meta-analysis. Cancer Treat Rev, 109, 102440.

Agache I; Song Y; Alonso-Coello P; Vogel Y; Rocha C; Solà I; Santero M; Akdis CA; Akdis M; Canonica GW; Chivato T; Del Giacco S; Eiwegger T; Fokkens W; Georgalas C; Gevaert P; Hopkins C; Klimek L; Lund V; Naclerio R; O'Mahony L; Palkonen S; Pfaar O; Schwarze J; Soyka MB; Wang Y; Zhang L; Canelo-Aybar C; Palomares O; Jutel M. 2021. Efficacy and safety of treatment with biologicals for severe chronic rhinosinusitis with nasal polyps: a systematic review for the EAACI guidelines. Allergy, 76, 2337-2353.

Pradhan RR; Nepal G; Mandal S. 2019. Safety and Efficacy of Mepolizumab in Patients with Eosinophilic Granulomatosis with Polyangiitis. Pulm Med, 2019, 4376380.

Steiger S; Ehreiser L; Anders J; Anders HJ. 2022. Biological drugs for systemic lupus erythematosus or active lupus nephritis and rates of infectious complications. Evidence from large clinical trials. Front Immunol, 13, 999704.

Lunn MP; Nobile-Orazio E. 2016. Immunotherapy for IgM anti-myelin-associated glycoprotein paraprotein-associated peripheral neuropathies. Cochrane Database Syst Rev, 10, CD002827.

Gatheral TL; Rushton A; Evans DJ; Mulvaney CA; Halcovitch NR; Whiteley G; Eccles FJ; Spencer S. 2017. Personalised asthma action plans for adults with asthma. Cochrane Database Syst Rev, 4, CD011859.

McClatchy J; Yap T; Koo K; Kern JS; Scardamaglia L. 2021. De-escalation of anti-CD20 monoclonal antibody (Rituximab) protocols in Pemphigus Vulgaris - a systematic review. Expert Opin Biol Ther, 21, 1591-1601.

Pereda CA; Nishishinya-Aquino MB; Brito-García N; Díaz Del Campo Fontecha P; Rua-Figueroa I. 2021. Is cotrimoxazole prophylaxis against Pneumocystis jirovecii pneumonia needed in patients with systemic autoimmune rheumatic diseases requiring immunosuppressive therapies? Rheumatol Int, 41, 1419-1427.

Lv KJ; Chen TC; Wang GH; Yao YN; Yao H. 2019. Clinical safety and efficacy of curcumin use for oral lichen planus: a systematic review. J Dermatolog Treat, 30, 605-611.

Peng W; Tang Y; Jiang Z; Li Z; Mi X; Qin W. 2016. The effect of calcineurin inhibitors in the treatment of IgA nephropathy: a systematic review and meta-analysis (PRISMA). Medicine (Baltimore), 95, e4731.

Jat KR; Walia DK; Khairwa A. 2018. Anti-IgE therapy for allergic bronchopulmonary aspergillosis in people with cystic fibrosis. Cochrane Database Syst Rev, 3, CD010288.

Whitton ME; Pinart M; Batchelor J; Leonardi-Bee J; González U; Jiyad Z; Eleftheriadou V; Ezzedine K. 2015. Interventions for vitiligo. Cochrane Database Syst Rev, 2015, CD003263.

**Exclusion reason: Wrong indication (n=44)**

Jin M; Douglass JA; Elborn JS; Agarwal R; Calhoun WJ; Lazarewicz S; Jaumont X; Yan M. 2023. Omalizumab in Allergic Bronchopulmonary Aspergillosis: a systematic review and meta-analysis. J Allergy Clin Immunol Pract, 11, 896-905.

Leong KJ; Lau T; Stewart V; Canetti EFD. 2021. Systematic Review and Meta-analysis: Effectiveness of Corticosteroids in Treating Adults With Acute Vestibular Neuritis. Otolaryngol Head Neck Surg, 165, 255-266.

Hur K; Zhou S; Kysh L. 2018. Adjunct steroids in the treatment of peritonsillar abscess: A systematic review. Laryngoscope, 128, 72-77.

Weijs TJ; Dieleman JM; Ruurda JP; Kroese AC; Knape HJ; van Hillegersberg R. 2014. The effect of perioperative administration of glucocorticoids on pulmonary complications after transthoracic oesophagectomy: a systematic review and meta-analysis. Eur J Anaesthesiol, 31, 685-94.

Yue C; Wei R; Liu Y. 2017. Perioperative systemic steroid for rapid recovery in total knee and hip arthroplasty: a systematic review and meta-analysis of randomized trials. J Orthop Surg Res, 12, 100.

Fabregat C; Almendros S; Navarro-Martin A; Gonzalez J. 2020. Pain Flare-Effect Prophylaxis With Corticosteroids on Bone Radiotherapy Treatment: A Systematic Review. Pain Pract, 20, 101-109.

Song TR; Jiang YM; Liu JP; Wang ZL; Zeng J; Huang ZL; Fan Y; Wang XD; Lin T. 2019. Steroid withdrawal or avoidance is safe in high-risk kidney transplants: a systematic review and meta-analysis. Kaohsiung J Med Sci, 35, 350-357.

Walters JA; Tan DJ; White CJ; Wood-Baker R. 2014. Different durations of corticosteroid therapy for exacerbations of chronic obstructive pulmonary disease. Cochrane Database Syst Rev, CD006897.

Quinto LR; Sukkar L; Gallagher M. 2019. Effectiveness of corticosteroid compared with non-corticosteroid therapy for the treatment of drug-induced acute interstitial nephritis: a systematic review. Intern Med J, 49, 562-569.

Koarai A; Yamada M; Ichikawa T; Fujino N; Sugiura H. 2024. Treatment with systemic corticosteroid versus placebo for exacerbations of COPD: a systematic review and meta-analysis. Respir Investig, 62, 503-511.

Papadopoulou E; Bin Safar S; Khalil A; Hansel J; Wang R; Corlateanu A; Kostikas K; Tryfon S; Vestbo J; Mathioudakis AG. 2024. Inhaled versus systemic corticosteroids for acute exacerbations of COPD: a systematic review and meta-analysis. Eur Respir Rev, 33.

Billy CA; Lim RT; Ruospo M; Palmer SC; Strippoli GFM. 2018. Corticosteroid or Nonsteroidal Antiinflammatory Drugs for the Treatment of Acute Gout: A Systematic Review of Randomized Controlled Trials. J Rheumatol, 45, 128-136.

Lopinto J; Gendreau S; Berti E; Bartolucci P; Habibi A; Mekontso Dessap A. 2022. Effects of corticosteroids in patients with sickle cell disease and acute complications: a systematic review and meta-analysis. Haematologica, 107, 1914-1921.

Mahalingam S; Luke L; Pundir J; Pundir V. 2021. The role of adjuvant systemic steroids in the management of periorbital cellulitis secondary to sinusitis: a systematic review and meta-analysis. Eur Arch Otorhinolaryngol, 278, 2193-2201.

Rowe BH; Kirkland SW; Vandermeer B; Campbell S; Newton A; Ducharme FM; Villa-Roel C. 2017. Prioritizing Systemic Corticosteroid Treatments to Mitigate Relapse in Adults With Acute Asthma: A Systematic Review and Network Meta-analysis. Acad Emerg Med, 24, 371-381.

Fairfield C; Penninga L; Powell J; Harrison EM; Wigmore SJ. 2015. Glucocorticosteroid-free versus glucocorticosteroid-containing immunosuppression for liver transplanted patients. Cochrane Database Syst Rev, CD007606.

Lee KE; Faye AS; Vermeire S; Shen B. 2022. Perioperative Management of Ulcerative Colitis: A Systematic Review. Dis Colon Rectum, 65, S5-S19.

Montero N; Pérez-Sáez MJ; Pascual J; Abramowicz D; Budde K; Dudley C; Hazzan M; Klinger M; Maggiore U; Oberbauer R; Sorensen SS; Viklicky O. 2016. Immunosuppression in the elderly renal allograft recipient: a systematic review. Transplant Rev (Orlando), 30, 144-53.

Oie Y; Komoto S; Kawasaki R. 2021. Systematic review of clinical research on regenerative medicine for the cornea. Jpn J Ophthalmol, 65, 169-183.

Turkoglu NM; Shang J. 2024. Fall risk factors in hospitalized bone marrow transplant patients: A systematic review. Int J Nurs Knowl, 35, 45995.

Kee AR; Gonzalez-Lopez JJ; Al-Hity A; Gupta B; Lee CS; Gunasekeran DV; Jayabalan N; Grant R; Kon OM; Gupta V; Westcott M; Pavesio C; Agrawal R. 2016. Anti-tubercular therapy for intraocular tuberculosis: a systematic review and meta-analysis. Surv Ophthalmol, 61, 628-53.

Shahzad M; Chaudhary SG; Basit A; Thellman C; Rodriguez L; Abhyankar SH; McGuirk JP; Mushtaq MU. 2022. Chronic graft-versus-host disease presenting as acute polymyositis: A case series and systematic review. Transpl Immunol, 70, 101520.

Ahmadzai N; Kilty S; Cheng W; Esmaeilisaraji L; Wolfe D; Bonaparte JP; Schramm D; Fitzpatrick E; Lin V; Skidmore B; Moher D; Hutton B. 2019. A systematic review and network meta-analysis of existing pharmacologic therapies in patients with idiopathic sudden sensorineural hearing loss. PLoS One, 14, e0221713.

Rashidi A; DiPersio JF; Sandmaier BM; Colditz GA; Weisdorf DJ. 2016. Steroids Versus Steroids Plus Additional Agent in Frontline Treatment of Acute Graft-versus-Host Disease: a systematic review and meta-analysis of Randomized Trials. Biol Blood Marrow Transplant, 22, 1133-1137.

Zhang Y; Jin W; Cai X. 2017. Anti-interleukin-2 receptor antibodies for the prevention of rejection in liver transplant recipients: a systematic review and meta-analysis. Ann Med, 49, 365-376.

Jiang X; Li Y; Chen N; Zhou M; He L. 2023. Corticosteroids for preventing postherpetic neuralgia. Cochrane Database Syst Rev, 12, CD005582.

Masson P; Henderson L; Chapman JR; Craig JC; Webster AC. 2014. Belatacept for kidney transplant recipients. Cochrane Database Syst Rev, 2014, CD010699.

Klein A; Cremer P; Kontzias A; Furqan M; Forsythe A; Crotty C; Lim-Watson M; Magestro M. 2022. Clinical Burden and Unmet Need in Recurrent Pericarditis: A Systematic Literature Review. Cardiol Rev, 30, 59-69.

Qiao X; Bilinski J; Wang L; Yang T; Luo R; Fu Y; Yang G. 2023. Safety and efficacy of fecal microbiota transplantation in the treatment of graft-versus-host disease. Bone Marrow Transplant, 58, 43739.

Tomlinson D; Robinson PD; Oberoi S; Cataudella D; Culos-Reed N; Davis H; Duong N; Gibson F; Götte M; Hinds P; Nijhof SL; van der Torre P; Cabral S; Dupuis LL; Sung L. 2018. Pharmacologic interventions for fatigue in cancer and transplantation: a meta-analysis. Curr Oncol, 25, e152-e167.

Montero N; Webster AC; Royuela A; Zamora J; Crespo Barrio M; Pascual J. 2014. Steroid avoidance or withdrawal for pancreas and pancreas with kidney transplant recipients. Cochrane Database Syst Rev, 2014, CD007669.

Ang SP; Chia JE; Jaiswal V; Hanif M; Vadhera A; Gautam S; Raut A; Rafaqat S; Borra VR; Khandait H; Shrestha AB; Iglesias J. 2024. Vascular complications and outcomes following transcatheter aortic valve replacement in patients on chronic steroid therapy: a meta-analysis. Int J Surg, 110, 2421-2429.

Suarez O; Pardo M; Gonzalez S; Escobar-Serna DP; Castaneda DA; Rodriguez D; Osorio JC; Lozano E. 2014. Diabetes mellitus and renal transplantation in adults: is there enough evidence for diagnosis, treatment, and prevention of new-onset diabetes after renal transplantation? Transplant Proc, 46, 3015-20.

van Durme CM; Wechalekar MD; Buchbinder R; Schlesinger N; van der Heijde D; Landewé RB. 2014. Non-steroidal anti-inflammatory drugs for acute gout. Cochrane Database Syst Rev, CD010120.

Tseng VL; Coleman AL; Chang MY; Caprioli J. 2017. Aqueous shunts for glaucoma. Cochrane Database Syst Rev, 7, CD004918.

Patel TK; Patel PB; Thakkar S. 2021. Comparison of effectiveness of interventions in reducing mortality in patients of toxic epidermal necrolysis: A network meta-analysis. Indian J Dermatol Venereol Leprol, 87, 628-644.

Caiazzo E; Rezig AOM; Bruzzese D; Ialenti A; Cicala C; Cleland JGF; Guzik TJ; Maffia P; Pellicori P. 2022. Systemic administration of glucocorticoids, cardiovascular complications and mortality in patients hospitalised with COVID-19, SARS, MERS or influenza: a systematic review and meta-analysis of randomised trials. Pharmacol Res, 176, 106053.

Woldeamanuel YW; Rapoport AM; Cowan RP. 2015. The place of corticosteroids in migraine attack management: A 65-year systematic review with pooled analysis and critical appraisal. Cephalalgia, 35, 996-1024.

Horita N; Otsuka T; Haranaga S; Namkoong H; Miki M; Miyashita N; Higa F; Takahashi H; Yoshida M; Kohno S; Kaneko T. 2015. Adjunctive Systemic Corticosteroids for Hospitalized Community-Acquired Pneumonia: Systematic Review and Meta-Analysis 2015 Update. Sci Rep, 5, 14061.

Sun Z; Xun R; Liu M; Wu X; Qu H. 2021. The Association Between Glucocorticoid Administration and the Risk of Impaired Efficacy of Axicabtagene Ciloleucel Treatment: A Systematic Review. Front Immunol, 12, 646450.

Junhai Z; Bangchuan H; Shijin G; Jing Y; Li L. 2021. Glucocorticoids for acute respiratory distress syndrome: a systematic review with meta-analysis and trial sequential analysis. Eur J Clin Invest, 51, e13496.

Loecher AM; West K; Quinn TD; Defayette AA. 2021. Management of diffuse alveolar hemorrhage in the hematopoietic stem cell transplantation population: A systematic review. Pharmacotherapy, 41, 943-952.

Zimmermann S; Sekula P; Venhoff M; Motschall E; Knaus J; Schumacher M; Mockenhaupt M. 2017. Systemic Immunomodulating Therapies for Stevens-Johnson Syndrome and Toxic Epidermal Necrolysis: a systematic review and meta-analysis. JAMA Dermatol, 153, 514-522.

Agache I; Song Y; Rocha C; Beltran J; Posso M; Steiner C; Alonso-Coello P; Akdis C; Akdis M; Canonica GW; Casale T; Chivato T; Corren J; Del Giacco S; Eiwegger T; Firinu D; Gern JE; Hamelmann E; Hanania N; Mäkelä M; Martín IH; Nair P; O'Mahony L; Papadopoulos NG; Papi A; Park HS; Pérez de Llano L; Quirce S; Sastre J; Shamji M; Schwarze J; Canelo-Aybar C; Palomares O; Jutel M. 2020. Efficacy and safety of treatment with dupilumab for severe asthma: A systematic review of the EAACI guidelines-Recommendations on the use of biologicals in severe asthma. Allergy, 75, 1058-1068.

**Exclusion reason: Wrong intervention (n=32)**

Bus SR; de Haan RJ; Vermeulen M; van Schaik IN; Eftimov F. 2024. Intravenous immunoglobulin for chronic inflammatory demyelinating polyradiculoneuropathy. Cochrane Database Syst Rev, 2, CD001797.

Ranawat A; Guo K; Phillips M; Guo A; Niazi F; Bhandari M; Waterman B. 2024. Health Economic Assessments of Hyaluronic Acid Treatments for Knee Osteoarthritis: a systematic review. Adv Ther, 41, 65-81.

Narváez J; Estrada P; LLop D; Vidal-Montal P; Brugarolas E; Maymó-Paituvi P; Palacios-Olid J; Nolla JM. 2023. Efficacy and safety of leflunomide in the management of large vessel vasculitis: a systematic review and metaanalysis of cohort studies. Semin Arthritis Rheum, 59, 152166.

Fujiwara T; Uraguchi K. 2024. Antiviral treatment for Ramsay Hunt syndrome: a systematic review and meta-analysis. Auris Nasus Larynx, 51, 488-491.

Bramuzzo M; Ventura A; Martelossi S; Lazzerini M. 2016. Thalidomide for inflammatory bowel disease: systematic review. Medicine (Baltimore), 95, e4239.

Oba Y; Anwer S; Maduke T; Patel T; Dias S. 2022. Effectiveness and tolerability of dual and triple combination inhaler therapies compared with each other and varying doses of inhaled corticosteroids in adolescents and adults with asthma: a systematic review and network meta-analysis. Cochrane Database Syst Rev, 12, CD013799.

Adler BL; Wang CJ; Bui TL; Schilperoort HM; Armstrong AW. 2019. Anti-tumor necrosis factor agents in sarcoidosis: a systematic review of efficacy and safety. Semin Arthritis Rheum, 48, 1093-1104.

Chandan S; Mohan BP; Kumar A; Khan SR; Chandan OC; Kassab LL; Ponnada S; Kochhar GS. 2021. Safety and efficacy of biological therapy in chronic antibiotic refractory pouchitis: a systematic review with meta-analysis. J Clin Gastroenterol, 55, 481-491.

Huang Y; Ren S; Yang Q. 2024. Efficacy and Safety of Excision Combination Therapies for Earlobe Keloids: a systematic review and meta-analysis. Aesthetic Plast Surg, 48, 2757-2770.

Zhen C; Hou Y; Zhao B; Ma X; Dai T; Yan C. 2022. Efficacy and safety of rituximab treatment in patients with idiopathic inflammatory myopathies: a systematic review and meta-analysis. Front Immunol, 13, 1051609.

Sconza C; Respizzi S; Virelli L; Vandenbulcke F; Iacono F; Kon E; Di Matteo B. 2020. Oxygen-ozone therapy for the treatment of knee osteoarthritis: a systematic review of randomized controlled trials. Arthroscopy, 36, 277-286.

Zhang J; Yu WQ; Chen W; Wei T; Wang CW; Zhang JY; Zhang Y; Liang TB. 2021. Systematic review and meta-analysis of the efficacy of appropriate empiric anti-enterococcal therapy for intra-abdominal infection. Surg Infect (Larchmt), 22, 131-143.

Melo FJ; Magina S. 2018. Clinical management of Anti-TNF-alpha-induced psoriasis or psoriasiform lesions in inflammatory bowel disease patients: a systematic review. Int J Dermatol, 57, 1521-1532.

Qiu X; Feng JR; Chen LP; Liu S; Zhang M; Zhou Z; Liu J; Zhao Q. 2017. Efficacy and safety of autologous hematopoietic stem cell therapy for refractory Crohn's disease: a systematic review and meta-analysis. Medicine (Baltimore), 96, e7381.

Eraso I; Sangiovanni S; Morales EI; Fernández-Trujillo L. 2021. Aspirin desensitization in NSAID-exacerbated respiratory disease and its outcomes in the clinical course of asthma: A systematic review of the literature and meta-analysis. PLoS One, 16, e0247871.

Mahmud O; Fatimi AS; Mahar MU; Jahangir A; Kashif A; Abbas M; Waljee AK; Berinstein JA. 2024. Efficacy and Safety of Sphingosine 1-Phosphate Receptor Modulators for Ulcerative Colitis: a systematic review and meta-analysis of Randomized Controlled Trials. J Clin Gastroenterol, 58, 753-763.

He C; Li W; Xie Q; Yin G. 2021. Rituximab in the Treatment of Interstitial Lung Diseases Related to Anti-Melanoma Differentiation-Associated Gene 5 Dermatomyositis: a systematic review. Front Immunol, 12, 820163.

Akram A; Ahmed M; Farhan K; Omer A; Kaleem S; Khan AT; Aslam U; Tahir MA; Memon S; Karam A; Furqan H; Anjum MU; Bhattarai P. 2024. Safety and efficacy of S1P receptor modulators for the induction and maintenance phases in inflammatory bowel disease: a systematic review and meta-analysis of randomized controlled trials. Medicine (Baltimore), 103, e39372.

Garegnani L; Hyland M; Roson Rodriguez P; Escobar Liquitay CME; Franco JV. 2021. Antioxidants to prevent respiratory decline in people with Duchenne muscular dystrophy and progressive respiratory decline. Cochrane Database Syst Rev, 11, CD013720.

Kew KM; Cates CJ. 2016. Remote versus face-to-face check-ups for asthma. Cochrane Database Syst Rev, 4, CD011715.

Normansell R; Kew KM; Stovold E. 2017. Interventions to improve adherence to inhaled steroids for asthma. Cochrane Database Syst Rev, 4, CD012226.

Maruani A; Samimi M; Stembridge N; Abdel Hay R; Tavernier E; Hughes C; Le Cleach L. 2019. Non-antistreptococcal interventions for acute guttate psoriasis or an acute guttate flare of chronic psoriasis. Cochrane Database Syst Rev, 4, CD011541.

Bourke JP; Bueser T; Quinlivan R. 2018. Interventions for preventing and treating cardiac complications in Duchenne and Becker muscular dystrophy and X-linked dilated cardiomyopathy. Cochrane Database Syst Rev, 10, CD009068.

Gonzalez-Lorenzo M; Ridley B; Minozzi S; Del Giovane C; Peryer G; Piggott T; Foschi M; Filippini G; Tramacere I; Baldin E; Nonino F. 2024. Immunomodulators and immunosuppressants for relapsing-remitting multiple sclerosis: a network meta-analysis. Cochrane Database Syst Rev, 1, CD011381.

Gong L; Xu M; Xu W; Tang W; Lu J; Jiang W; Xie F; Ding L; Qian X. 2021. Efficacy and safety of tacrolimus monotherapy versus cyclophosphamide-corticosteroid combination therapy for idiopathic membranous nephropathy: a meta-analysis. Medicine (Baltimore), 100, e26628.

Ni H; Yu H; Lin Q; Zhong J; Sun W; Nie H. 2023. Analysis of risk factors of fungal superinfections in viral pneumonia patients: a systematic review and meta-analysis. Immun Inflamm Dis, 11, e760.

Crossingham I; Turner S; Ramakrishnan S; Fries A; Gowell M; Yasmin F; Richardson R; Webb P; O'Boyle E; Hinks TS. 2021. Combination fixed-dose beta agonist and steroid inhaler as required for adults or children with mild asthma. Cochrane Database Syst Rev, 5, CD013518.

Maxwell LJ; Zochling J; Boonen A; Singh JA; Veras MM; Tanjong Ghogomu E; Benkhalti Jandu M; Tugwell P; Wells GA. 2015. TNF-alpha inhibitors for ankylosing spondylitis. Cochrane Database Syst Rev, 2015, CD005468.

Ni H; Soe Z; Moe S. 2014. Aclidinium bromide for stable chronic obstructive pulmonary disease. Cochrane Database Syst Rev, 2014, CD010509.

Arnaud L; Gavand PE; Voll R; Schwarting A; Maurier F; Blaison G; Magy-Bertrand N; Pennaforte JL; Peter HH; Kieffer P; Bonnotte B; Poindron V; Fiehn C; Lorenz H; Amoura Z; Sibilia J; Martin T. 2019. Predictors of fatigue and severe fatigue in a large international cohort of patients with systemic lupus erythematosus and a systematic review of the literature. Rheumatology (Oxford), 58, 987-996.

Tian Z; Li Y; Xie Y; Yang Y; Xu J. 2022. Efficacy and safety of tacrolimus combined with corticosteroids in patients with idiopathic membranous nephropathy: a systematic review and meta-analysis of randomized controlled trials. Int Urol Nephrol, 54, 2555-2566.

Kokkinidis DG; Bosdelekidou EE; Iliopoulou SM; Tassos AG; Texakalidis PT; Economopoulos KP; Kousoulis AA. 2017. Emerging treatments for ulcerative colitis: a systematic review. Scand J Gastroenterol, 52, 923-931.

**Exclusion reason: Wrong population (n=41)**

Hahn D; Hodson EM; Willis NS; Craig JC. 2015. Interventions for preventing and treating kidney disease in Henoch-Schönlein Purpura (HSP). Cochrane Database Syst Rev, 2015, CD005128.

Bell JM; Shields MD; Watters J; Hamilton A; Beringer T; Elliott M; Quinlivan R; Tirupathi S; Blackwood B. 2017. Interventions to prevent and treat corticosteroid-induced osteoporosis and prevent osteoporotic fractures in Duchenne muscular dystrophy. Cochrane Database Syst Rev, 1, CD010899.

Zheng Q; He Q; Huang H; Lu M. 2023. Venous sinus thrombosis in a case of immunoglobulin A vasculitis and a systemic review of literature. Int J Rheum Dis, 26, 539-543.

Polderman JA; Farhang-Razi V; Van Dieren S; Kranke P; DeVries JH; Hollmann MW; Preckel B; Hermanides J. 2018. Adverse side effects of dexamethasone in surgical patients. Cochrane Database Syst Rev, 11, CD011940.

Caradu C; Ammollo RP; Dari L; Wanhainen A; Van Herzeele I; Bellmunt-Montoya S; Ducasse E; Bérard X. 2023. Management of Inflammatory Aortic Aneurysms - A Scoping Review. Eur J Vasc Endovasc Surg, 65, 493-502.

Xiong A; Luo W; Tang X; Cao Y; Xiang Q; Deng R; Shuai S. 2023. Risk factors for invasive fungal infections in patients with connective tissue disease: Systematic review and meta-analysis. Semin Arthritis Rheum, 63, 152257.

Jones IA; LoBasso MA; Wier J; Gettleman BS; Richardson MK; Ratto CE; Lieberman JR; Heckmann ND. 2024. Perioperative Dexamethasone in Diabetic Patients: a systematic review and meta-analysis of Randomized, Placebo-Controlled Trials. Anesth Analg, 139, 479-489.

Normansell R; Kew KM; Mansour G. 2016. Different oral corticosteroid regimens for acute asthma. Cochrane Database Syst Rev, 2016, CD011801.

Wang JY; Pao JB; Lee CH; Lee MC; Wu TT. 2023. Corticosteroids for COVID-19-induced olfactory dysfunction: a comprehensive systematic review and meta-analysis of randomized controlled trials. PLoS One, 18, e0289172.

Køppen KS; Gasbjerg KS; Andersen JH; Hägi-Pedersen D; Lunn TH; Mathiesen O. 2023. Systemic glucocorticoids as an adjunct to treatment of postoperative pain after total hip and knee arthroplasty: a systematic review with meta-analysis and trial sequential analysis. Eur J Anaesthesiol, 40, 155-170.

Gu YL; Pang J; Sun ZX; Hu J; Sun Y; Wu XW; Guo JJ; Yang GS. 2020. Comparative efficacies of nebulized budesonide and systemic corticosteroids in the treatment of exacerbations of chronic obstructive pulmonary disease: a systematic review and meta-analysis. J Clin Pharm Ther, 45, 419-429.

Reicy R; Jari M. 2024. Comparison of Different Treatment Regimens for Long-term Improvement of Renal Function in Patients with Henoch-Schönlein Purpura: a systematic review. Curr Rheumatol Rev, 20, 57-64.

Crayne CB; Mitchell C; Beukelman T. 2019. Comparison of second-line therapy in IVIg-refractory Kawasaki disease: a systematic review. Pediatr Rheumatol Online J, 17, 77.

García-Pavón S; Yamazaki-Nakashimada MA; Báez M; Borjas-Aguilar KL; Murata C. 2017. Kawasaki Disease Complicated With Macrophage Activation Syndrome: a systematic review. J Pediatr Hematol Oncol, 39, 445-451.

Penn J; Douglas W; Curran J; Chaudhuri D; Dionne JC; Fernando SM; Granton D; Mathew R; Rochwerg B. 2023. Efficacy and safety of corticosteroids in cardiac arrest: a systematic review, meta-analysis and trial sequential analysis of randomized control trials. Crit Care, 27, 12.

Barret M; Beye B; Leblanc S; Beuvon F; Chaussade S; Batteux F; Prat F. 2015. Systematic review: the prevention of oesophageal stricture after endoscopic resection. Aliment Pharmacol Ther, 42, 20-39.

Obeidat A; Silangcruz K; Kozai L; Wien E; Fujiwara Y; Nishimura Y. 2022. Clinical Characteristics and Outcomes of Gastritis Associated With Immune Checkpoint Inhibitors: Scoping Review. J Immunother, 45, 363-369.

Gao R; Li Y; Cao Y; Zheng R; Tang L; Yang J; Lu X. 2020. Glucocorticoid versus traditional therapy for hepatitis B virus-related acute-on-chronic liver failure: a systematic review and meta-analysis. Medicine (Baltimore), 99, e20604.

Zhou J; Wang CP; Li J; Zhang HL; He CX. 2024. Stevens-Johnson syndrome and toxic epidermal necrolysis associated with immune checkpoint inhibitors: a systematic review. Front Immunol, 15, 1414136.

Fairfield C; Penninga L; Powell J; Harrison EM; Wigmore SJ. 2018. Glucocorticosteroid-free versus glucocorticosteroid-containing immunosuppression for liver transplanted patients. Cochrane Database Syst Rev, 4, CD007606.

Lee CM; Wang M; Rajkumar A; Calabrese C; Calabrese L. 2024. A scoping review of vasculitis as an immune-related adverse event from checkpoint inhibitor therapy of cancer: Unraveling the complexities at the intersection of immunology and vascular pathology. Semin Arthritis Rheum, 66, 152440.

Martino EA; Baiardo Redaelli M; Sardo S; Lembo R; Giordano VF; Winterton D; Ruggeri L; Hajjar LA; Zangrillo A; Landoni G. 2018. Steroids and Survival in Critically Ill Adult Patients: A Meta-analysis of 135 Randomized Trials. J Cardiothorac Vasc Anesth, 32, 2252-2260.

Pathak R; Katel A; Massarelli E; Villaflor VM; Sun V; Salgia R. 2021. Immune Checkpoint Inhibitor-Induced Myocarditis with Myositis/Myasthenia Gravis Overlap Syndrome: A Systematic Review of Cases. Oncologist, 26, 1052-1061.

Biyun L; Yahui H; Yuanfang L; Xifeng G; Dao W. 2024. Risk factors for invasive fungal infections after haematopoietic stem cell transplantation: a systematic review and meta-analysis. Clin Microbiol Infect, 30, 601-610.

Oliveras L; Coloma A; Lloberas N; Lino L; Favà A; Manonelles A; Codina S; Couceiro C; Melilli E; Sharif A; Hecking M; Guthoff M; Cruzado JM; Pascual J; Montero N. 2024. Immunosuppressive drug combinations after kidney transplantation and post-transplant diabetes: a systematic review and meta-analysis. Transplant Rev (Orlando), 38, 100856.

Meserve J; Facciorusso A; Holmer AK; Annese V; Sandborn WJ; Singh S. 2021. Systematic review with meta-analysis: safety and tolerability of immune checkpoint inhibitors in patients with pre-existing inflammatory bowel diseases. Aliment Pharmacol Ther, 53, 374-382.

Yang J; Wang X; Li Y; Lu G; Lu X; Guo D; Wang W; Liu C; Xiao Y; Han N; He S. 2019. Efficacy and safety of steroid in the prevention of esophageal stricture after endoscopic submucosal dissection: A network meta-analysis. J Gastroenterol Hepatol, 34, 985-995.

Alharbi A; Khobrani A; Noor A; Alghamdi W; Alotaibi A; Alnuhait M; Haseeb A. 2022. Risk of Lichen Sclerosus and Lichen Planus in Patients Receiving Immune Checkpoint Inhibitors. Int J Environ Res Public Health, 20.

Hill P; Cross NB; Barnett AN; Palmer SC; Webster AC. 2017. Polyclonal and monoclonal antibodies for induction therapy in kidney transplant recipients. Cochrane Database Syst Rev, 1, CD004759.

Liu XH; Li JY; Qu XH; Yan WL; Zhang L; Yang C; Zheng JW. 2016. Treatment of kaposiform hemangioendothelioma and tufted angioma. Int J Cancer, 139, 1658-66.

Mongioì LM; Condorelli RA; Barbagallo F; La Vignera S; Calogero AE. 2020. Dual-release hydrocortisone for treatment of adrenal insufficiency: a systematic review. Endocrine, 67, 507-515.

Lamers OAC; Smits BM; Leavis HL; de Bree GJ; Cunningham-Rundles C; Dalm VASH; Ho HE; Hurst JR; IJspeert H; Prevaes SMPJ; Robinson A; van Stigt AC; Terheggen-Lagro S; van de Ven AAJM; Warnatz K; van de Wijgert JHHM; van Montfrans J. 2021. Treatment Strategies for GLILD in Common Variable Immunodeficiency: A Systematic Review. Front Immunol, 12, 606099.

Kirkland SW; Cross E; Campbell S; Villa-Roel C; Rowe BH. 2018. Intramuscular versus oral corticosteroids to reduce relapses following discharge from the emergency department for acute asthma. Cochrane Database Syst Rev, 6, CD012629.

Ma C; MacDonald JK; Nguyen TM; Vande Casteele N; Linggi B; Lefevre P; Wang Y; Feagan BG; Jairath V. 2022. Pharmacological Interventions for the Prevention and Treatment of Immune Checkpoint Inhibitor-Associated Enterocolitis: A Systematic Review. Dig Dis Sci, 67, 1128-1155.

Costa BA; Costa TA; Saravia SD; Felix N; Tan CR; Korde N; Richter J. 2024. Thromboembolic risk of carfilzomib or bortezomib in combination with lenalidomide and dexamethasone for newly diagnosed multiple myeloma: A comparative systematic review and meta-analysis. Am J Hematol, 99, 1056-1065.

Giri M; Puri A; Wang T; Guo S. 2021. Comparison of clinical manifestations, pre-existing comorbidities, complications and treatment modalities in severe and non-severe COVID-19 patients: A systemic review and meta-analysis. Sci Prog, 104, 368504211000906.

Cheng K; Ashby D; Smyth RL. 2015. Oral steroids for long-term use in cystic fibrosis. Cochrane Database Syst Rev, 2015, CD000407.

Mir H; Alhussein M; Alrashidi S; Alzayer H; Alshatti A; Valettas N; Mukherjee SD; Nair V; Leong DP. 2018. Cardiac Complications Associated With Checkpoint Inhibition: A Systematic Review of the Literature in an Important Emerging Area. Can J Cardiol, 34, 1059-1068.

Sahebnasagh A; Najmeddin F; Najafi A; Saghafi F; Salehi-Abargouei A; Ahmadi A; Amini S; Mojtahedzadeh M; Sharifnia H. 2022. Efficacy of Glucocorticoid Administration in Patients with Cardiac Arrest: A Systematic Review of Clinical Studies. Curr Med Chem, 29, 136-151.

Nagendrababu V; Pulikkotil SJ; Jinatongthai P; Veettil SK; Teerawattanapong N; Gutmann JL. 2019. Efficacy and Safety of Oral Premedication on Pain after Nonsurgical Root Canal Treatment: A Systematic Review and Network Meta-analysis of Randomized Controlled Trials. J Endod, 45, 364-371.

Fong KY; Zhao JJ; Syn NL; Nair P; Chan YH; Lee P. 2023. Comparing bronchial thermoplasty with biologicals for severe asthma: Systematic review and network meta-analysis. Respir Med, 216, 107302.

**Exclusion reason: Wrong route of administration (n=33)**

Lodi G; Manfredi M; Mercadante V; Murphy R; Carrozzo M. 2020. Interventions for treating oral lichen planus: corticosteroid therapies. Cochrane Database Syst Rev, 2, CD001168.

Simforoosh N; Nayebzade A; Dadpour M; Rohani S. 2023. Corticosteroid Dose in Kidney Transplantation and Its Effect on Surgical Complication: A Systematic Review. Exp Clin Transplant, 21, 631-638.

Jessop S; Whitelaw DA; Grainge MJ; Jayasekera P. 2017. Drugs for discoid lupus erythematosus. Cochrane Database Syst Rev, 5, CD002954.

Gordon A; Roe T; Villar-Martínez MD; Moreno-Ajona D; Goadsby PJ; Hoffmann J. 2023. Effectiveness and safety profile of greater occipital nerve blockade in cluster headache: a systematic review. J Neurol Neurosurg Psychiatry, 95, 73-85.

Crossingham I; Turner S; Ramakrishnan S; Fries A; Gowell M; Yasmin F; Richardson R; Webb P; O'Boyle E; Hinks TSC. 2022. Combination fixed-dose ß agonist and steroid inhaler as required for adults or children with mild asthma: a Cochrane systematic review. BMJ Evid Based Med, 27, 178-184.

Zhao X; Zhou C; Ma J; Zhu Y; Sun M; Wang P; Zhang Y; Ma H; Zhang H. 2017. Efficacy and safety of rectal 5-aminosalicylic acid versus corticosteroids in active distal ulcerative colitis: a systematic review and network meta-analysis. Sci Rep, 7, 46693.

Zhao X; Li N; Ren Y; Ma T; Wang C; Wang J; You S. 2016. Efficacy and Safety of Beclomethasone Dipropionate versus 5-Aminosalicylic Acid in the Treatment of Ulcerative Colitis: a systematic review and meta-analysis. PLoS One, 11, e0160500.

Hashizume H; Ishikawa Y; Ajima S. 2022. Is steroid pulse therapy a suitable treatment for drug-induced hypersensitivity syndrome/drug reaction with eosinophilia and systemic symptoms? A systematic review of case reports in patients treated with corticosteroids in Japan. J Dermatol, 49, 303-307.

Bunjo LJ; Bacchi S; Pietris J; Chan WO. 2024. Current management options for the treatment of refractory postoperative cystoid macular edema: A systematic review. Surv Ophthalmol, 69, 606-621.

Kew KM; Quinn M; Quon BS; Ducharme FM. 2016. Increased versus stable doses of inhaled corticosteroids for exacerbations of chronic asthma in adults and children. Cochrane Database Syst Rev, 2016, CD007524.

Feeley AA; Feeley TB; Feeley IH; Sheehan E. 2021. Postoperative Infection Risk in Total Joint Arthroplasty After Perioperative IV Corticosteroid Administration: a systematic review and meta-analysis of Comparative Studies. J Arthroplasty, 36, 3042-3053.

Lee ACH; Riedl M; Frommer L; Diana T; Kahaly GJ. 2020. Systemic safety analysis of mycophenolate in Graves' orbitopathy. J Endocrinol Invest, 43, 767-777.

Shen WC; Lee CH; Loh EW; Hsieh AT; Chen L; Tam KW. 2018. Efficacy and Safety of Rituximab for the Treatment of Graves' Orbitopathy: A Meta-analysis of Randomized Controlled Trials. Pharmacotherapy, 38, 503-510.

Fairley JL; Oon S; Saracino AM; Nikpour M. 2020. Management of cutaneous manifestations of lupus erythematosus: A systematic review. Semin Arthritis Rheum, 50, 95-127.

Jacobsen A; Olabi B; Langley A; Beecker J; Mutter E; Shelley A; Worley B; Ramsay T; Saavedra A; Parker R; Stewart F; Pardo Pardo J. 2022. Systemic interventions for treatment of Stevens-Johnson syndrome (SJS), toxic epidermal necrolysis (TEN), and SJS/TEN overlap syndrome. Cochrane Database Syst Rev, 3, CD013130.

Huo L; Liu G; Deng B; Xu L; Mo Y; Jiang S; Tao J; Bai H; Wang L; Yang X; Yang J; Mu X. 2024. Effect of use of NSAIDs or steroids during the acute phase of pain on the incidence of chronic pain: a systematic review and meta-analysis of randomised trials. Inflammopharmacology, 32, 1039-1058.

Arora CJ; Rafiq M; Shumack S; Gupta M. 2020. The efficacy and safety of tacrolimus as mono- and adjunctive therapy for vitiligo: A systematic review of randomised clinical trials. Australas J Dermatol, 61, e1-e9.

Gläser S; Krüger S; Merkel M; Bramlage P; Herth FJ. 2015. Chronic obstructive pulmonary disease and diabetes mellitus: a systematic review of the literature. Respiration, 89, 253-64.

Fu S; Duan L; Zhong Y; Zeng Y. 2024. Comparison of surgical excision followed by adjuvant radiotherapy and laser combined with steroids for the treatment of keloids: a systematic review and meta-analysis. Int Wound J, 21, e14449.

Sliwka A; Jankowski M; Gross-Sondej I; Storman M; Nowobilski R; Bala MM. 2018. Once-daily long-acting beta2-agonists/inhaled corticosteroids combined inhalers versus inhaled long-acting muscarinic antagonists for people with chronic obstructive pulmonary disease. Cochrane Database Syst Rev, 8, CD012355.

Chauhan BF; Jeyaraman MM; Singh Mann A; Lys J; Abou-Setta AM; Zarychanski R; Ducharme FM. 2017. Addition of anti-leukotriene agents to inhaled corticosteroids for adults and adolescents with persistent asthma. Cochrane Database Syst Rev, 3, CD010347.

Ding Y; Sun L; Wang Y; Zhang J; Chen Y. 2022. Efficacy of ICS versus Non-ICS Combination Therapy in COPD: A Meta-Analysis of Randomised Controlled Trials. Int J Chron Obstruct Pulmon Dis, 17, 1051-1067.

Underwood M. 2015. Gout. BMJ Clin Evid, 2015, .

Mysler E; Burmester GR; Saffore CD; Liu J; Wegrzyn L; Yang C; Betts KA; Wang Y; Irvine AD; Panaccione R. 2024. Safety of Upadacitinib in Immune-Mediated Inflammatory Diseases: Systematic Literature Review of Indirect and Direct Treatment Comparisons of Randomized Controlled Trials. Adv Ther, 41, 567-597.

Bradshaw SE; Gala S; Nanavaty M; Shah A; Mwamburi M; Kefalas P. 2016. Systematic literature review of treatments for management of complications of ischemic central retinal vein occlusion. BMC Ophthalmol, 16, 104.

Edwards Mayhew RG; Li T; McCann P; Leslie L; Strong Caldwell A; Palestine AG. 2022. Non-biologic, steroid-sparing therapies for non-infectious intermediate, posterior, and panuveitis in adults. Cochrane Database Syst Rev, 10, CD014831.

Adas MA; Allen VB; Yates M; Bechman K; Clarke BD; Russell MD; Rutherford AI; Cope AP; Norton S; Galloway JB. 2021. A systematic review and network meta-analysis of the safety of early interventional treatments in rheumatoid arthritis. Rheumatology (Oxford), 60, 4450-4462.

Normansell R; Kew KM; Bridgman AL. 2015. Sublingual immunotherapy for asthma. Cochrane Database Syst Rev, 2015, CD011293.

Chong J; Haran C; Chauhan BF; Asher I. 2015. Intermittent inhaled corticosteroid therapy versus placebo for persistent asthma in children and adults. Cochrane Database Syst Rev, 2015, CD011032.

Fortescue R; Kew KM; Leung MST. 2020. Sublingual immunotherapy for asthma. Cochrane Database Syst Rev, 9, CD011293.

Ni H; Htet A; Moe S. 2017. Umeclidinium bromide versus placebo for people with chronic obstructive pulmonary disease (COPD). Cochrane Database Syst Rev, 6, CD011897.

Hilliquin S; Hugues B; Mitrovic S; Gossec L; Fautrel B. 2018. Ability of disease-modifying antirheumatic drugs to prevent or delay rheumatoid arthritis onset: a systematic literature review and meta-analysis. Ann Rheum Dis, 77, 1099-1106.

Tramacere I; Virgili G; Perduca V; Lucenteforte E; Benedetti MD; Capobussi M; Castellini G; Frau S; Gonzalez-Lorenzo M; Featherstone R; Filippini G. 2023. Adverse effects of immunotherapies for multiple sclerosis: a network meta-analysis. Cochrane Database Syst Rev, 11, CD012186.

**Exclusion reason: Wrong study design (n=35)**

Tan ST; Boyle V; Elston MS. 2023. Systematic Review of Therapeutic Agents and Long-Term Outcomes of Familial Hyperaldosteronism Type 1. Hypertension, 80, 1517-1525.

Dwivedi P; Kumar RR; Dhooria A; Adarsh MB; Malhotra S; Kakkar N; Naidu S; Sharma SK; Sharma A; Jain S; Dhir V. 2019. Corticosteroid-associated lupus pancreatitis: a case series and systematic review of the literature. Lupus, 28, 731-739.

Cheah JTL; Robson JC; Black RJ; Goodman SM; Lester S; Mackie SL; Hill CL. 2020. The patient's perspective of the adverse effects of glucocorticoid use: A systematic review of quantitative and qualitative studies. From an OMERACT working group. Semin Arthritis Rheum, 50, 996-1005.

Guo FQ; Deng M. 2019. Correlation Between Steroid-Induced Osteonecrosis of The Femoral Head and Hepatic CYP3A Activity: a systematic review and meta-analysis. J Invest Surg, 32, 118-126.

Narváez J; Mora-Limiñana M; Ros I; Ibañez M; Valldeperas J; Crémer D; Nolla JM; Juan-Mas A. 2019. Pulmonary arterial hypertension in adult-onset Still's disease: a case series and systematic review of the literature. Semin Arthritis Rheum, 49, 162-170.

Nannini S; Koshenkova L; Baloglu S; Chaussemy D; Noël G; Schott R. 2022. Immune-related aseptic meningitis and strategies to manage immune checkpoint inhibitor therapy: a systematic review. J Neurooncol, 157, 533-550.

Garg RK; Rizvi I; Chakravarty R; Malhotra HS; Kumar N. 2024. Treatment Outcome in Patients with Disseminated Cysticercosis: a systematic review of case reports and case series. Am J Trop Med Hyg, 110, 1080-1088.

Chatzigeorgiou C; Mackie SL. 2018. Comorbidity in polymyalgia rheumatica. Reumatismo, 70, 35-43.

Haber R; Bachour J; El Gemayel M. 2020. Scleromyxedema treatment: a systematic review and update. Int J Dermatol, 59, 1191-1201.

Carver CA; Kalesinskas M; Ahmed AR. 2023. Current biologics in treatment of pemphigus foliaceus: a systematic review. Front Immunol, 14, 1267668.

Xia D; Chen X; Zhou Q; Xiao S; Yu Y; Wang Y; Du G; Huang H; Zhang W; Chen Y. 2017. Efficacy of Purtscher's Retinopathy Treatments: a systematic review. Curr Eye Res, 42, 908-917.

Villa E; Sarquis T; de Grazia J; Núñez R; Alarcón P; Villegas R; Guevara C. 2021. Rheumatoid meningitis: a systematic review and meta-analysis. Eur J Neurol, 28, 3201-3210.

Drago F; Ciccarese G; Agnoletti AF; Sarocchi F; Parodi A. 2017. Neuro sweet syndrome: a systematic review. A rare complication of Sweet syndrome. Acta Neurol Belg, 117, 33-42.

Boyadzhieva Z; Ruffer N; Kötter I; Krusche M. 2023. How to treat VEXAS syndrome: a systematic review on effectiveness and safety of current treatment strategies. Rheumatology (Oxford), 62, 3518-3525.

Karadag SG; Sönmez HE; Tanatar A; Çakan M; Aktay Ayaz N. 2020. Isotretinoin-induced sacroiliitis: Case series of four patients and a systematic review of the literature. Pediatr Dermatol, 37, 171-175.

Chatzidionysiou K; Emamikia S; Nam J; Ramiro S; Smolen J; van der Heijde D; Dougados M; Bijlsma J; Burmester G; Scholte M; van Vollenhoven R; Landewé R. 2017. Efficacy of glucocorticoids, conventional and targeted synthetic disease-modifying antirheumatic drugs: a systematic literature review informing the 2016 update of the EULAR recommendations for the management of rheumatoid arthritis. Ann Rheum Dis, 76, 1102-1107.

Bueno-Molina RC; Hernández-Rodríguez JC; Cabrera-Fuentes R; Cabrera-Pérez R; Conejo-Mir Sánchez J; Pereyra-Rodríguez JJ. 2024. Advances in treatment for lipoid proteinosis (Urbach-Wiethe disease): a case report and systematic review. Clin Exp Dermatol, 49, 547-555.

Muñoz-Ortiz J; Reyes-Guanes J; Zapata-Bravo E; Mora-Muñoz L; Reyes-Hurtado JA; Tierradentro-García LO; Rojas-Carabali W; Gómez-Suarez M; de-la-Torre A. 2021. Ocular adverse events from pharmacological treatment in patients with multiple sclerosis-A systematic review of the literature. Syst Rev, 10, 280.

Nguyen B; Acharya C; Tangpanithandee S; Miao J; Krisanapan P; Thongprayoon C; Amir O; Mao MA; Cheungpasitporn W; Acharya PC. 2023. Efficacy and Safety of Plasma Exchange as an Adjunctive Therapy for Rapidly Progressive IgA Nephropathy and Henoch-Schönlein Purpura Nephritis: A Systematic Review. Int J Mol Sci, 24.

Olabi B; Worboys S; Garland T; Grindlay DJC; Rogers NK; Harman KE. 2020. What's new in atopic eczema? An analysis of systematic reviews published in 2018. Part 2: systemic therapies. Clin Exp Dermatol, 45, 980-985.

Corradini D; Di Matteo A; Emery P; Mankia K. 2021. How should we treat palindromic rheumatism? A systematic literature review. Semin Arthritis Rheum, 51, 266-277.

Akiyama M; Kaneko Y; Takeuchi T. 2020. Tocilizumab for the treatment of TAFRO syndrome: a systematic literature review. Ann Hematol, 99, 2463-2475.

Akiyama M; Kaneko Y; Takeuchi T. 2020. Effectiveness of tocilizumab in Behcet's disease: A systematic literature review. Semin Arthritis Rheum, 50, 797-804.

Damian L; Pamfil C; Buc?a C; Nicula C; Mouthon L; Amoura Z; Cutolo M; Burmester GR; Fonseca JE; Grapini L; Arnaud L; Rednic S. 2022. Rare within rare. Necrotising scleritis and peripheral ulcerative keratitis: eye-threatening complications of relapsing polychondritis. Clin Exp Rheumatol, 40 Suppl 134, 86-92.

Callander J; Robson Y; Ingram J; Piguet V. 2018. Treatment of clinically amyopathic dermatomyositis in adults: a systematic review. Br J Dermatol, 179, 1248-1255.

Zerdes I; Tolia M; Nikolaou M; Tsoukalas N; Velentza L; Hajiioannou J; Mitsis M; Kyrgias G. 2017. How can we effectively address the paraneoplastic dermatomyositis: Diagnosis, risk factors and treatment options. J BUON, 22, 1073-1080.

Yang LY; Wang YL; Zuo YG. 2024. Pemphigoid diseases in patients with end-stage kidney diseases: pathogenesis and treatment. Front Immunol, 15, 1427943.

Lazaros G; Imazio M; Brucato A; Vassilopoulos D; Vasileiou P; Gattorno M; Tousoulis D; Martini A. 2016. Anakinra: an emerging option for refractory idiopathic recurrent pericarditis: a systematic review of published evidence. J Cardiovasc Med (Hagerstown), 17, 256-62.

Decker P; Olivier P; Risse J; Zuily S; Wahl D. 2018. Tocilizumab and refractory Takayasu disease: Four case reports and systematic review. Autoimmun Rev, 17, 353-360.

Goldfarb JM; Rabinowitz MR; Basnyat S; Nyquist GG; Rosen MR. 2016. Head and Neck Manifestations of Eosinophilic Granulomatosis with Polyangiitis: A Systematic Review. Otolaryngol Head Neck Surg, 155, 771-778.

Liu Y; Shi Y; Ren R; Xie J; Wang W; Chen N. 2018. Advanced therapeutics in focal and segmental glomerulosclerosis. Nephrology (Carlton), 23 Suppl 4, 57-61.

Lytvyn Y; Mufti A; Maliyar K; Sachdeva M; Yeung J. 2022. Onset of Pyoderma Gangrenosum in Patients on Biologic Therapies: A Systematic Review. Adv Skin Wound Care, 35, 454-460.

Yajima N; Tsujimoto Y; Fukuma S; Sada KE; Shimizu S; Niihata K; Takahashi R; Asano Y; Azuma T; Kameda H; Kuwana M; Kohsaka H; Sugiura-Ogasawara M; Suzuki K; Takeuchi T; Tanaka Y; Tamura N; Matsui T; Mimori T; Fukuhara S; Atsumi T. 2020. The development of quality indicators for systemic lupus erythematosus using electronic health data: A modified RAND appropriateness method. Mod Rheumatol, 30, 525-531.

Lyne SA; Ruediger C; Lester S; Kaur G; Stamp L; Shanahan EM; Hill CL. 2023. Clinical phenotype and complications of large vessel giant cell arteritis: a systematic review and meta-analysis. Joint Bone Spine, 90, 105558.

Zeng Y; Duan J; Ge G; Zhang M. 2021. Therapeutic Management of Ocular Ischemia in Takayasu's Arteritis: A Case-Based Systematic Review. Front Immunol, 12, 791278.

**Exclusion reason: Relevant data not separable (n= 14)**

Deng HS; Hou YW; Zhang JN; Yang T. 2023. Postauricular versus systemic use of steroids for sudden hearing loss: a systematic review and meta-analysis of randomized controlled trials. Medicine (Baltimore), 102, e34494.

Ge Y; Zhou H; Shi J; Ye B; Peng Q; Lu X; Wang G. 2015. The efficacy of tacrolimus in patients with refractory dermatomyositis/polymyositis: a systematic review. Clin Rheumatol, 34, 2097-103.

Darmawan G; Liman LMS; Wibowo SAK; Hamijoyo L; Apriani L; Atik N; Alisjahbana B; Sahiratmadja E. 2024. Global tuberculosis disease and infection in systemic lupus erythematosus patients: a systematic review and meta-analysis. Lupus, 33, 555-573.

Matthews E; Brassington R; Kuntzer T; Jichi F; Manzur AY. 2016. Corticosteroids for the treatment of Duchenne muscular dystrophy. Cochrane Database Syst Rev, 2016, CD003725.

Abdel Shaheed C; Maher CG; Buchbinder R; Ng B; Enke O; Guzowski R; McLachlan AJ; Day RO; Richards B; Latimer J; Lin CC. 2020. Efficacy and harms of orally, intramuscularly or intravenously administered glucocorticoids for sciatica: a systematic review and meta-analysis. Eur J Pain, 24, 518-535.

Jung F; Sibbald C; Bohdanowicz M; Ingram JR; Piguet V. 2020. Systematic review of the efficacies and adverse effects of treatments for pityriasis lichenoides. Br J Dermatol, 183, 1026-1032.

Tariq S; Niaz F; Waseem S; Shaikh TG; Ahmed SH; Irfan M; Nashwan AJ; Ullah I. 2023. Managing and treating Sydenham chorea: a systematic review. Brain Behav, 13, e3035.

Kessi M; Liu F; Zhan Y; Tang Y; Wu L; Yang L; Zhang CL; Yin F; Peng J. 2020. Efficacy of different treatment modalities for acute and chronic phases of the febrile infection-related epilepsy syndrome: a systematic review. Seizure, 79, 61-68.

Rawla P; Sunkara T; Thandra KC; Gaduputi V. 2018. Efficacy and Safety of Budesonide in the Treatment of Eosinophilic Esophagitis: Updated Systematic Review and Meta-Analysis of Randomized and Non-Randomized Studies. Drugs R D, 18, 259-269.

Godazandeh G; Shojaee L; Alizadeh-Navaei R; Hessami A. 2021. Corticosteroids in idiopathic granulomatous mastitis: a systematic review and meta-analysis. Surg Today, 51, 1897-1905.

Cahyadi M; Mesinovic J; Chim ST; Ebeling P; Zengin A; Grech L. 2023. Medication and bone health in multiple sclerosis: a systematic review and meta-analysis. J Manag Care Spec Pharm, 29, 1331-1353.

Oren-Shabtai M; Kremer N; Lapidoth M; Sharon E; Atzmony L; Nosrati A; Hodak E; Mimouni D; Levi A. 2021. Treatment of Bullous Pemphigoid in People Aged 80 Years and Older: a systematic review of the literature. Drugs Aging, 38, 125-136.

Misra DP; Rathore U; Patro P; Agarwal V; Sharma A. 2021. Corticosteroid monotherapy for the management of Takayasu arteritis-a systematic review and meta-analysis. Rheumatol Int, 41, 1729-1742.

Wang LR; Barber CE; Johnson AS; Barnabe C. 2014. Invasive fungal disease in systemic lupus erythematosus: a systematic review of disease characteristics, risk factors, and prognosis. Semin Arthritis Rheum, 44, 325-30.

# Data S6: References to included studies

ABE, N., OKU, K., AMENGUAL, O., FUJIEDA, Y., KATO, M., BOHGAKI, T., YASUDA, S., MORI, R., MORISHITA, E., SUZUKI-INOUE, K. & ATSUMI, T. 2020. Potential therapeutics for antiphospholipid antibody associated thrombocytopenia: A systematic review and meta-analysis. *Mod Rheumatol,* 30**,** 116-124.

ÁGUEDA, A. F., MONTI, S., LUQMANI, R. A., BUTTGEREIT, F., CID, M., DASGUPTA, B., DEJACO, C., MAHR, A., PONTE, C., SALVARANI, C., SCHMIDT, W. & HELLMICH, B. 2019. Management of Takayasu arteritis: a systematic literature review informing the 2018 update of the EULAR recommendation for the management of large vessel vasculitis. *RMD Open,* 5**,** e001020.

AKIYAMA, M., KANEKO, Y. & TAKEUCHI, T. 2020. Tocilizumab in isolated polymyalgia rheumatica: A systematic literature review. *Semin Arthritis Rheum,* 50**,** 521-525.

AKIYAMA, M., KANEKO, Y. & TAKEUCHI, T. 2021. Rituximab for the treatment of eosinophilic granulomatosis with polyangiitis: A systematic literature review. *Autoimmun Rev,* 20**,** 102737.

AL EFRAIJ, K., JOHNSON, K. M., WIEBE, D., SADATSAFAVI, M. & FITZGERALD, J. M. 2019. A systematic review of the adverse events and economic impact associated with oral corticosteroids in asthma. *J Asthma,* 56**,** 1334-1346.

ALCHI, M. B., LEVER, R., FLOSSMANN, O. & JAYNE, D. 2023. Efficacy and safety of low- versus high-dose glucocorticoid regimens for induction of remission of anti-neutrophil cytoplasm antibody-associated vasculitis: a systematic review and meta-analysis. *Scand J Rheumatol,* 52**,** 564-573.

ALLEN, C. S., YEUNG, J. H., VANDERMEER, B. & HOMIK, J. 2016. Bisphosphonates for steroid-induced osteoporosis. *Cochrane Database Syst Rev,* 10**,** CD001347.

AMICHE, M. A., ALBAUM, J. M., TADROUS, M., PECHLIVANOGLOU, P., LÉVESQUE, L. E., ADACHI, J. D. & CADARETTE, S. M. 2016. Efficacy of osteoporosis pharmacotherapies in preventing fracture among oral glucocorticoid users: a network meta-analysis. *Osteoporos Int,* 27**,** 1989-98.

ANTONIO, A. A., SANTOS, R. N. & ABARIGA, S. A. 2022. Tocilizumab for giant cell arteritis. *Cochrane Database Syst Rev,* 5**,** CD013484.

ARAI, Y., JO, T., MATSUI, H., KONDO, T. & TAKAORI-KONDO, A. 2018. Comparison of up-front treatments for newly diagnosed immune thrombocytopenia -a systematic review and network meta-analysis. *Haematologica,* 103**,** 163-171.

ATZMONY, L., HODAK, E., GDALEVICH, M., ROSENBAUM, O. & MIMOUNI, D. 2014. Treatment of pemphigus vulgaris and pemphigus foliaceus: a systematic review and meta-analysis. *Am J Clin Dermatol,* 15**,** 503-15.

ATZMONY, L., HODAK, E., LESHEM, Y. A., ROSENBAUM, O., GDALEVICH, M., ANHALT, G. J. & MIMOUNI, D. 2015. The role of adjuvant therapy in pemphigus: A systematic review and meta-analysis. *J Am Acad Dermatol,* 73**,** 264-71.

ATZMONY, L., REITER, O., HODAK, E., GDALEVICH, M. & MIMOUNI, D. 2016. Treatments for Cutaneous Lichen Planus: A Systematic Review and Meta-Analysis. *Am J Clin Dermatol,* 17**,** 11-22.

AZUKAITIS, K., PALMER, S. C., STRIPPOLI, G. F. & HODSON, E. M. 2022. Interventions for minimal change disease in adults with nephrotic syndrome. *Cochrane Database Syst Rev,* 3**,** CD001537.

BARBA, T., FORT, R., COTTIN, V., PROVENCHER, S., DURIEU, I., JARDEL, S., HOT, A., REYNAUD, Q. & LEGA, J. C. 2019. Treatment of idiopathic inflammatory myositis associated interstitial lung disease: A systematic review and meta-analysis. *Autoimmun Rev,* 18**,** 113-122.

BERGSTRA, S. A., SEPRIANO, A., KERSCHBAUMER, A., VAN DER HEIJDE, D., CAPORALI, R., EDWARDS, C. J., VERSCHUEREN, P., DE SOUZA, S., POPE, J. E., TAKEUCHI, T., HYRICH, K. L., WINTHROP, K. L., ALETAHA, D., STAMM, T. A., SCHOONES, J. W., SMOLEN, J. S. & LANDEWÉ, R. B. M. 2023. Efficacy, duration of use and safety of glucocorticoids: a systematic literature review informing the 2022 update of the EULAR recommendations for the management of rheumatoid arthritis. *Ann Rheum Dis,* 82**,** 81-94.

BITOSSI, A., MATTIOLI, I., BETTIOL, A., PALERMO, A., MALANDRINO, D., BACHERINI, D., VIRGILI, G., GIANSANTI, F., VANNOZZI, L. & SILVESTRI, E. 2023. Non-anti TNFα biologic agents for noninfectious uveitis associated with systemic inflammatory diseases: a systematic review. *Expert Rev Clin Immunol,* 19**,** 549-560.

BLACK, R. J., HILL, C. L., LESTER, S. & DIXON, W. G. 2016. The Association between Systemic Glucocorticoid Use and the Risk of Cataract and Glaucoma in Patients with Rheumatoid Arthritis: A Systematic Review and Meta-Analysis. *PLoS One,* 11**,** e0166468.

BLAVNSFELDT, A. G., DE THURAH, A., THOMSEN, M. D., TARP, S., LANGDAHL, B. & HAUGE, E. M. 2018. The effect of glucocorticoids on bone mineral density in patients with rheumatoid arthritis: A systematic review and meta-analysis of randomized, controlled trials. *Bone,* 114**,** 172-180.

BLEECKER, E. R., MENZIES-GOW, A. N., PRICE, D. B., BOURDIN, A., SWEET, S., MARTIN, A. L., ALACQUA, M. & TRAN, T. N. 2020. Systematic Literature Review of Systemic Corticosteroid Use for Asthma Management. *Am J Respir Crit Care Med,* 201**,** 276-293.

BONOVAS, S., NIKOLOPOULOS, G. K., LYTRAS, T., FIORINO, G., PEYRIN-BIROULET, L. & DANESE, S. 2018. Comparative safety of systemic and low-bioavailability steroids in inflammatory bowel disease: Systematic review and network meta-analysis. *Br J Clin Pharmacol,* 84**,** 239-251.

BOSE, B., CHUNG, E. Y. M., HONG, R., STRIPPOLI, G. F. M., JOHNSON, D. W., YANG, W. L., BADVE, S. V. & PALMER, S. C. 2022. Immunosuppression therapy for idiopathic membranous nephropathy: systematic review with network meta-analysis. *J Nephrol,* 35**,** 1159-1170.

BREAKEY, S., SHARP, S. J., ADLER, A. I. & CHALLIS, B. G. 2016. Glucocorticoid-induced hyperglycaemia in respiratory disease: a systematic review and meta-analysis. *Diabetes Obes Metab,* 18**,** 1274-1278.

BRITO-ZERÓN, P., KOSTOV, B., BOSCH, X., ACAR-DENIZLI, N., RAMOS-CASALS, M. & STONE, J. H. 2016. Therapeutic approach to IgG4-related disease: A systematic review. *Medicine (Baltimore),* 95**,** e4002.

BROERSEN, L. H., PEREIRA, A. M., JORGENSEN, J. O. & DEKKERS, O. M. 2015. Adrenal Insufficiency in Corticosteroids Use: Systematic Review and Meta-Analysis. *J Clin Endocrinol Metab,* 100**,** 2171-80.

BUTTGEREIT, F., DEJACO, C., MATTESON, E. L. & DASGUPTA, B. 2016. Polymyalgia Rheumatica and Giant Cell Arteritis: A Systematic Review. *JAMA,* 315**,** 2442-58.

CAMPBELL, A. M., MARTIN, J. R. & ERSTAD, B. L. 2020. Corticosteroid Tapering Regimens in Rheumatic Disease: A Systematic Review. *J Clin Rheumatol,* 26**,** 41-47.

CHALITSIOS, C. V., SHAW, D. E. & MCKEEVER, T. M. 2021. Corticosteroids and bone health in people with asthma: A systematic review and meta-analysis. *Respir Med,* 181**,** 106374.

CHANDE, N., PATTON, P. H., TSOULIS, D. J., THOMAS, B. S. & MACDONALD, J. K. 2015. Azathioprine or 6-mercaptopurine for maintenance of remission in Crohn's disease. *Cochrane Database Syst Rev,* 2015**,** CD000067.

CHANDE, N., TOWNSEND, C. M., PARKER, C. E. & MACDONALD, J. K. 2016. Azathioprine or 6-mercaptopurine for induction of remission in Crohn's disease. *Cochrane Database Syst Rev,* 10**,** CD000545.

CHANG, Y. P., LAI, C. H., LIN, C. Y., CHANG, Y. C., LIN, M. C., CHONG, I. W., SHEU, C. C., WEI, Y. F., CHU, K. A., TSAI, J. R., LEE, C. H. & CHEN, Y. C. 2019. Mortality and vertebral fracture risk associated with long-term oral steroid use in patients with chronic obstructive pulmonary disease: A systemic review and meta-analysis. *Chron Respir Dis,* 16**,** 1479973119838280.

CHAVEZ-ALVAREZ, S., HERZ-RUELAS, M., RAYGOZA-CORTEZ, A. K., SURO-SANTOS, Y., OCAMPO-CANDIANI, J., ALVAREZ-VILLALOBOS, N. A. & VILLARREAL-MARTINEZ, A. 2021. Oral mini-pulse therapy in vitiligo: a systematic review. *Int J Dermatol,* 60**,** 868-876.

CHEN, X., ZHI, H., WANG, X., ZHOU, Z., LUO, H., LI, J., SEHMI, R., O'BYRNE, P. M. & CHEN, R. 2024. Efficacy of Biologics in Patients with Allergic Bronchopulmonary Aspergillosis: A Systematic Review and Meta-Analysis. *Lung,* 202**,** 367-383.

CHIANG, H. Y., GUO, Z. A., WU, T. W. & PENG, T. R. 2022. Efficacy and safety of belimumab therapy in systemic lupus erythematosus: A systematic review and meta-analysis. *Lupus,* 31**,** 666-673.

COBO-IBÁÑEZ, T., LOZA-SANTAMARÍA, E., PEGO-REIGOSA, J. M., MARQUÉS, A. O., RÚA-FIGUEROA, I., FERNÁNDEZ-NEBRO, A., CÁLIZ CÁLIZ, R., LÓPEZ LONGO, F. J. & MUÑOZ-FERNÁNDEZ, S. 2014. Efficacy and safety of rituximab in the treatment of non-renal systemic lupus erythematosus: a systematic review. *Semin Arthritis Rheum,* 44**,** 175-85.

DAI, X. M., YIN, M. M., LIU, Y., MA, L. L., YING, J. & JIANG, L. D. 2020. Advancements in medical and surgical treatments of Takayasu arteritis-induced renal arteritis: a systematic review. *Chin Med J (Engl),* 133**,** 975-981.

DE BOCK, M. & SIENAERT, P. 2024. Corticosteroids and mania: A systematic review. *World J Biol Psychiatry,* 25**,** 161-174.

D'HAENS, G. 2016. Systematic review: second-generation vs. conventional corticosteroids for induction of remission in ulcerative colitis. *Aliment Pharmacol Ther,* 44**,** 1018-1029.

DHIR, V., MISHRA, D. & SAMANTA, J. 2021. Glucocorticoids in spondyloarthritis-systematic review and real-world analysis. *Rheumatology (Oxford),* 60**,** 4463-4475.

DURAZZO, M., LUPI, G., SCANDELLA, M., FERRO, A. & GRUDEN, G. 2019. Autoimmune hepatitis treatment in the elderly: A systematic review. *World J Gastroenterol,* 25**,** 2809-2818.

EDEL, Y., AVNI, T., SHEPSHELOVICH, D., REICH, S., ROZEN-ZVI, B., ELBAZ, M., LEIBOVICI, L., MOLAD, Y. & GAFTER-GVILI, A. 2020. The safety of pulse corticosteroid therapy- Systematic review and meta-analysis. *Semin Arthritis Rheum,* 50**,** 534-545.

ELSOURI, K. N., ARBOLEDA, V., BASBOUS, L., HEISER, S., COLLINS, D. P., RAGUSA, P., BAXTER, C., CABRERA, D., AKHAND, T., STERMER, E., SHARMA, K., SEGURO, C., HARDIGAN, P., KESSELMAN, M. & BECKLER, M. D. 2023. Glucocorticoid use in rheumatoid arthritis patients and the onset of pneumonia: a systematic review and meta-analysis. *J Osteopath Med,* 123**,** 179-186.

ETCHEGARAY-MORALES, I., MENDOZA-PINTO, C., MUNGUÍA-REALPOZO, P., SOLIS-POBLANO, J. C., MÉNDEZ-MARTÍNEZ, S., AYÓN-AGUILAR, J., ABUD-MENDOZA, C., GARCÍA-CARRASCO, M. & CERVERA, R. 2024. Risk of diabetes mellitus in systemic lupus erythematosus: systematic review and meta-analysis. *Rheumatology (Oxford),* 63**,** 2047-2055.

FIGUEROA-PARRA, G., CUÉLLAR-GUTIÉRREZ, M. C., GONZÁLEZ-TREVIÑO, M., SANCHEZ-RODRIGUEZ, A., FLORES-GOUYONNET, J., MEADE-AGUILAR, J. A., PROKOP, L. J., MURAD, M. H., DALL'ERA, M., ROVIN, B. H., HOUSSIAU, F., TAMIROU, F., FERVENZA, F. C., CROWSON, C. S., PUTMAN, M. S. & DUARTE-GARCÍA, A. 2024. Impact of Glucocorticoid Dose on Complete Response, Serious Infections, and Mortality During the Initial Therapy of Lupus Nephritis: A Systematic Review and Meta-Analysis of the Control Arms of Randomized Controlled Trials. *Arthritis Rheumatol,* 76**,** 1408-1418.

FLORIS, A., PIGA, M., CHESSA, E., CONGIA, M., ERRE, G. L., ANGIONI, M. M., MATHIEU, A. & CAULI, A. 2022. Long-term glucocorticoid treatment and high relapse rate remain unresolved issues in the real-life management of polymyalgia rheumatica: a systematic literature review and meta-analysis. *Clin Rheumatol,* 41**,** 19-31.

FUJIWARA, T., NAMEKAWA, M., KURIYAMA, A. & TAMAKI, H. 2019. High-dose Corticosteroids for Adult Bell's Palsy: Systematic Review and Meta-analysis. *Otol Neurotol,* 40**,** 1101-1108.

GIACOMELLI, R., SOTA, J., RUSCITTI, P., CAMPOCHIARO, C., COLAFRANCESCO, S., DAGNA, L., IACONO, D., IANNONE, F., LOPALCO, G., SFRISO, P. & CANTARINI, L. 2021. The treatment of adult-onset Still's disease with anakinra, a recombinant human IL-1 receptor antagonist: a systematic review of literature. *Clin Exp Rheumatol,* 39**,** 187-195.

GÓMEZ-GÓMEZ, A., LOZA, E., ROSARIO, M. P., ESPINOSA, G., MORALES, J., HERRERAS, J. M., MUÑOZ-FERNÁNDEZ, S. & CORDERO-COMA, M. 2017. Efficacy and safety of immunomodulatory drugs in patients with anterior uveitis: A systematic literature review. *Medicine (Baltimore),* 96**,** e8045.

GOSWAMI, R. P., HALDAR, S. N., CHATTERJEE, M., VIJ, P., VAN DER KOOI, A. J., LIM, J., RAAPHORST, J., BHADU, D., GELARDI, C., DANIELI, M. G. & KUMAR, U. 2022. Efficacy and safety of intravenous and subcutaneous immunoglobulin therapy in idiopathic inflammatory myopathy: A systematic review and meta-analysis. *Autoimmun Rev,* 21**,** 102997.

GUAN, R., LIN, Y., ZHANG, C., WANG, Z., WU, Z., LIU, X., CHEN, X. & PIAO, Y. 2024. Comparative efficacy and safety of systemic steroids, oral JAK inhibitors and Contact Immunotherapy in the Treatment of severe alopecia areata: a systematic review and network meta-analysis. *Arch Dermatol Res,* 316**,** 483.

GUAN, X., ZHAO, Z., XIN, M., XIA, G., YANG, Q. & FU, M. 2024. Long-term efficacy, safety, and cumulative retention rate of antitumor necrosis factor-alpha treatment for patients with Behcet's uveitis: A systematic review and meta-analysis. *Int J Rheum Dis,* 27**,** e15096.

HEAD, K., CHONG, L. Y., HOPKINS, C., PHILPOTT, C., BURTON, M. J. & SCHILDER, A. G. 2016. Short-course oral steroids alone for chronic rhinosinusitis. *Cochrane Database Syst Rev,* 4**,** CD011991.

HEAD, K., CHONG, L. Y., HOPKINS, C., PHILPOTT, C., SCHILDER, A. G. & BURTON, M. J. 2016. Short-course oral steroids as an adjunct therapy for chronic rhinosinusitis. *Cochrane Database Syst Rev,* 4**,** CD011992.

HEAD, K., CHONG, L. Y., PIROMCHAI, P., HOPKINS, C., PHILPOTT, C., SCHILDER, A. G. & BURTON, M. J. 2016. Systemic and topical antibiotics for chronic rhinosinusitis. *Cochrane Database Syst Rev,* 4**,** CD011994.

HMAMOUCHI, I., PARUK, F., TABRA, S., MAATALLAH, K., BOUZIANE, A., ABOUQAL, R., EL MAIDANY, Y., EL MAGHRAOUI, A. & KALLA, A. A. 2023. Prevalence of glucocorticoid-induced osteoporosis among rheumatology patients in Africa: a systematic review and meta-analysis. *Arch Osteoporos,* 18**,** 59.

HUGHES, R. A., MEHNDIRATTA, M. M. & RAJABALLY, Y. A. 2017. Corticosteroids for chronic inflammatory demyelinating polyradiculoneuropathy. *Cochrane Database Syst Rev,* 11**,** CD002062.

HYSA, E., BOND, M., EHLERS, L., CAMELLINO, D., FALZON, L., DEJACO, C., BUTTGEREIT, F., ALETAHA, D. & KERSCHBAUMER, A. 2024. Evidence on treat to target strategies in polymyalgia rheumatica and giant cell arteritis: a systematic literature review. *Rheumatology (Oxford),* 63**,** 285-297.

IHEOZOR-EJIOFOR, Z., GORDON, M., CLEGG, A., FREEMAN, S. C., GJULADIN-HELLON, T., MACDONALD, J. K. & AKOBENG, A. K. 2019. Interventions for maintenance of surgically induced remission in Crohn's disease: a network meta-analysis. *Cochrane Database Syst Rev,* 9**,** CD013210.

ISRAEL, E., CANONICA, G. W., BRUSSELLE, G., YANG, S., HOWARTH, P. H., MARTIN, A. L., KOUFOPOULOU, M., SMITH, S. G. & ALFONSO-CRISTANCHO, R. 2022. Real-life effectiveness of mepolizumab in severe asthma: a systematic literature review. *J Asthma,* 59**,** 2201-2217.

JAFARZADEH, A., POUR MOHAMMAD, A., KHOSRAVI, M., AMIRI, S., RASOULI, A., KERAMATI, H. & GOODARZI, A. 2024. A systematic review of case series and clinical trials investigating systemic oral or injectable therapies for the treatment of vitiligo. *Skin Res Technol,* 30**,** e13642.

JI, L., XIE, W., FASANO, S. & ZHANG, Z. 2022. Risk factors of flare in patients with systemic lupus erythematosus after glucocorticoids withdrawal. A systematic review and meta-analysis. *Lupus Sci Med,* 9.

JI, L., XIE, W. & ZHANG, Z. 2020. Efficacy and safety of sirolimus in patients with systemic lupus erythematosus: A systematic review and meta-analysis. *Semin Arthritis Rheum,* 50**,** 1073-1080.

JI, L., XIE, W. & ZHANG, Z. 2021. Low-dose glucocorticoids should be withdrawn or continued in systemic lupus erythematosus? A systematic review and meta-analysis on risk of flare and damage accrual. *Rheumatology (Oxford),* 60**,** 5517-5526.

JOSEPH, R. M., HUNTER, A. L., RAY, D. W. & DIXON, W. G. 2016. Systemic glucocorticoid therapy and adrenal insufficiency in adults: A systematic review. *Semin Arthritis Rheum,* 46**,** 133-41.

KAFIL, T. S., NGUYEN, T. M., PATTON, P. H., MACDONALD, J. K., CHANDE, N. & MCDONALD, J. W. 2017. Interventions for treating collagenous colitis. *Cochrane Database Syst Rev,* 11**,** CD003575.

KANG, L., LIU, Y., LUO, Z., ZHOU, Y., CHEN, B., YIN, G. & XIE, Q. 2023. Systematic review and meta-analysis of the current literature on tocilizumab in patients with refractory Takayasu arteritis. *Front Immunol,* 14**,** 1084558.

KOLKHIR, P., GRAKHOVA, M., BONNEKOH, H., KRAUSE, K. & MAURER, M. 2019. Treatment of urticarial vasculitis: A systematic review. *J Allergy Clin Immunol,* 143**,** 458-466.

KONING, A., VAN DER MEULEN, M., SCHAAP, D., SATOER, D. D., VINKERS, C. H., VAN ROSSUM, E. F. C., VAN FURTH, W. R., PEREIRA, A. M., MEIJER, O. C. & DEKKERS, O. M. 2024. Neuropsychiatric Adverse Effects of Synthetic Glucocorticoids: A Systematic Review and Meta-Analysis. *J Clin Endocrinol Metab,* 109**,** e1442-e1451.

KULKARNI, S., DURHAM, H., GLOVER, L., ATHER, O., PHILLIPS, V., NEMES, S., COUSENS, L., BLOMGRAN, P. & AMBERY, P. 2022. Metabolic adverse events associated with systemic corticosteroid therapy-a systematic review and meta-analysis. *BMJ Open,* 12**,** e061476.

KWAK, S. G., CHOO, Y. J. & CHANG, M. C. 2022. Effectiveness of prednisolone in complex regional pain syndrome treatment: A systematic narrative review. *Pain Pract,* 22**,** 381-390.

LATTANZI, S., CAGNETTI, C., DANNI, M., PROVINCIALI, L. & SILVESTRINI, M. 2017. Oral and intravenous steroids for multiple sclerosis relapse: a systematic review and meta-analysis. *J Neurol,* 264**,** 1697-1704.

LI, L., BENSING, S. & FALHAMMAR, H. 2021. Rate of fracture in patients with glucocorticoid replacement therapy: a systematic review and meta-analysis. *Endocrine,* 74**,** 29-37.

LIN, Y., JIA, J., GUO, Y., HE, D., ZHANG, Y., WANG, F., YAN, T., LIU, Y. & LIN, S. 2018. Corticosteroid for IgA Nephropathy: Are They Really Therapeutic? *Am J Nephrol,* 47**,** 385-394.

LIN, Z., ZHAO, Y., LI, F., LI, J. & HUANG, X. 2023. Efficacy and safety of biological agents for pemphigoid: a systematic review and meta-analysis. *Int J Dermatol,* 62**,** 1000-1008.

LIU, A. P. & CHEUK, D. K. 2021. Disease-modifying treatments for primary autoimmune haemolytic anaemia. *Cochrane Database Syst Rev,* 3**,** CD012493.

LIU, X., WANG, H., BRIDLE, K., CRAWFORD, D. & LIANG, X. 2022. Efficacy and safety of immune-modulating therapy for primary sclerosing cholangitis: A systematic review and meta-analysis. *Pharmacol Ther,* 237**,** 108163.

MADHOK, V. B., GAGYOR, I., DALY, F., SOMASUNDARA, D., SULLIVAN, M., GAMMIE, F. & SULLIVAN, F. 2016. Corticosteroids for Bell's palsy (idiopathic facial paralysis). *Cochrane Database Syst Rev,* 7**,** CD001942.

MANGUSO, F., BENNATO, R., LOMBARDI, G., RICCIO, E., COSTANTINO, G. & FRIES, W. 2016. Efficacy and Safety of Oral Beclomethasone Dipropionate in Ulcerative Colitis: A Systematic Review and Meta-Analysis. *PLoS One,* 11**,** e0166455.

MATEOS-HARO, M., NOVOA-CANDIA, M., SÁNCHEZ VANEGAS, G., CORREA-PÉREZ, A., GAETANO GIL, A., FERNÁNDEZ-GARCÍA, S., ORTEGA-QUIJANO, D., URUEÑA RODRIGUEZ, M. G., SACEDA-CORRALO, D., BENNOUNA-DALERO, T., GIRALDO, L., TOMLINSON, J., VAÑO-GALVÁN, S. & ZAMORA, J. 2023. Treatments for alopecia areata: a network meta-analysis. *Cochrane Database Syst Rev,* 10**,** CD013719.

MCMILLAN, R., TAYLOR, J., SHEPHARD, M., AHMED, R., CARROZZO, M., SETTERFIELD, J., GRANDO, S., MIGNOGNA, M., KUTEN-SHORRER, M., MUSBAH, T., ELIA, A., MCGOWAN, R., KERR, A. R., GREENBERG, M. S., HODGSON, T. & SIROIS, D. 2015. World Workshop on Oral Medicine VI: a systematic review of the treatment of mucocutaneous pemphigus vulgaris. *Oral Surg Oral Med Oral Pathol Oral Radiol,* 120**,** 132-42.e61.

MING, S., XIE, K., HE, H., LI, Y. & LEI, B. 2018. Efficacy and safety of adalimumab in the treatment of non-infectious uveitis: a meta-analysis and systematic review. *Drug Des Devel Ther,* 12**,** 2005-2016.

MISRA, D. P., RATHORE, U., PATRO, P., AGARWAL, V. & SHARMA, A. 2021. Disease-modifying anti-rheumatic drugs for the management of Takayasu arteritis-a systematic review and meta-analysis. *Clin Rheumatol,* 40**,** 4391-4416.

MITHOOWANI, S., GREGORY-MILLER, K., GOY, J., MILLER, M. C., WANG, G., NOROOZI, N., KELTON, J. G. & ARNOLD, D. M. 2016. High-dose dexamethasone compared with prednisone for previously untreated primary immune thrombocytopenia: a systematic review and meta-analysis. *Lancet Haematol,* 3**,** e489-e496.

MOJA, L., DANESE, S., FIORINO, G., DEL GIOVANE, C. & BONOVAS, S. 2015. Systematic review with network meta-analysis: comparative efficacy and safety of budesonide and mesalazine (mesalamine) for Crohn's disease. *Aliment Pharmacol Ther,* 41**,** 1055-65.

MOLOOGHI, K., SHEYBANI, F., NADERI, H., MIRFEIZI, Z., MOROVATDAR, N. & BARADARAN, A. 2022. Central nervous system infections in patients with systemic lupus erythematosus: a systematic review and meta-analysis. *Lupus Sci Med,* 9.

NARULA, N., DHILLON, A., ZHANG, D., SHERLOCK, M. E., TONDEUR, M. & ZACHOS, M. 2018. Enteral nutritional therapy for induction of remission in Crohn's disease. *Cochrane Database Syst Rev,* 4**,** CD000542.

NATALE, P., PALMER, S. C., RUOSPO, M., SAGLIMBENE, V. M., CRAIG, J. C., VECCHIO, M., SAMUELS, J. A., MOLONY, D. A., SCHENA, F. P. & STRIPPOLI, G. F. 2020. Immunosuppressive agents for treating IgA nephropathy. *Cochrane Database Syst Rev,* 3**,** CD003965.

OAKLANDER, A. L., LUNN, M. P., HUGHES, R. A., VAN SCHAIK, I. N., FROST, C. & CHALK, C. H. 2017. Treatments for chronic inflammatory demyelinating polyradiculoneuropathy (CIDP): an overview of systematic reviews. *Cochrane Database Syst Rev,* 1**,** CD010369.

OMAR, D., CHEN, Y., CONG, Y. & DONG, L. 2020. Glucocorticoids and steroid sparing medications monotherapies or in combination for IgG4-RD: a systematic review and network meta-analysis. *Rheumatology (Oxford),* 59**,** 718-726.

OON, S., HUQ, M., GODFREY, T. & NIKPOUR, M. 2018. Systematic review, and meta-analysis of steroid-sparing effect, of biologic agents in randomized, placebo-controlled phase 3 trials for systemic lupus erythematosus. *Semin Arthritis Rheum,* 48**,** 221-239.

OSMAN, M., PAGNOUX, C., DRYDEN, D. M., STORIE, D. & YACYSHYN, E. 2014. The role of biological agents in the management of large vessel vasculitis (LVV): a systematic review and meta-analysis. *PLoS One,* 9**,** e115026.

OZGULER, Y., LECCESE, P., CHRISTENSEN, R., ESATOGLU, S. N., BANG, D., BODAGHI, B., ÇELIK, A. F., FORTUNE, F., GAUDRIC, J., GUL, A., KÖTTER, I., MAHR, A., MOOTS, R. J., RICHTER, J., SAADOUN, D., SALVARANI, C., SCUDERI, F., SFIKAKIS, P. P., SIVA, A., STANFORD, M., TUGAL-TUTKUN, I., WEST, R., YURDAKUL, S., OLIVIERI, I., YAZICI, H. & HATEMI, G. 2018. Management of major organ involvement of Behçet's syndrome: a systematic review for update of the EULAR recommendations. *Rheumatology (Oxford),* 57**,** 2200-2212.

PAGLIA, M. D. G., SILVA, M. T., LOPES, L. C., BARBERATO-FILHO, S., MAZZEI, L. G., ABE, F. C. & DE CÁSSIA BERGAMASCHI, C. 2021. Use of corticoids and non-steroidal anti-inflammatories in the treatment of rheumatoid arthritis: Systematic review and network meta-analysis. *PLoS One,* 16**,** e0248866.

PALMOWSKI, A., NIELSEN, S. M., BOYADZHIEVA, Z., SCHNEIDER, A., PANKOW, A., HARTMAN, L., DA SILVA, J. A. P., KIRWAN, J., WASSENBERG, S., DEJACO, C., CHRISTENSEN, R., BOERS, M. & BUTTGEREIT, F. 2023. Safety and efficacy associated with long-term low-dose glucocorticoids in rheumatoid arthritis: a systematic review and meta-analysis. *Rheumatology (Oxford),* 62**,** 2652-2660.

PALMOWSKI, A., PANKOW, A., TERZIYSKA, K., NIELSEN, S. M., CHRISTENSEN, R., BLIDDAL, H., BOYADZHIEVA, Z. & BUTTGEREIT, F. 2024. Continuing versus tapering low-dose glucocorticoids in patients with rheumatoid arthritis and systemic lupus erythematosus in states of low disease activity or remission: A systematic review and meta-analysis of randomised trials. *Semin Arthritis Rheum,* 64**,** 152349.

PARTRIDGE, A. C. R., BAI, J. W., ROSEN, C. F., WALSH, S. R., GULLIVER, W. P. & FLEMING, P. 2018. Effectiveness of systemic treatments for pyoderma gangrenosum: a systematic review of observational studies and clinical trials. *Br J Dermatol,* 179**,** 290-295.

PHINYO, P., KRIKEERATI, T., VICHARA-ANONT, I. & THONGNGARM, T. 2024. Efficacy and Safety of Biologics for Oral Corticosteroid-Dependent Asthma: A Systematic Review and Network Meta-Analysis. *J Allergy Clin Immunol Pract,* 12**,** 409-420.

PITRE, T., KAWANO-DOURADO, L., KACHKOVSKI, G. V., LEUNG, D., LEUNG, G., DESAI, K., ZHAI, C., ADAMS, W., FUNKE-CHAMBOUR, M., KREUTER, M., STEWART, I., RYERSON, C. J., JENKINS, G. & ZERAATKAR, D. 2023. Systemic corticosteroids in fibrotic lung disease: a systematic review and meta-analysis. *BMJ Open Respir Res,* 10.

PRADO, M. B., JR. & ADIAO, K. J. B. 2023. Methotrexate in generalized myasthenia gravis: a systematic review. *Acta Neurol Belg,* 123**,** 1679-1691.

RAPOSO, A., TANI, C., COSTA, J. & MOSCA, M. 2016. Human papillomavirus infection and cervical lesions in rheumatic diseases: a systematic review. *Acta Reumatol Port,* 41**,** 184-190.

REZAIE, A., KUENZIG, M. E., BENCHIMOL, E. I., GRIFFITHS, A. M., OTLEY, A. R., STEINHART, A. H., KAPLAN, G. G. & SEOW, C. H. 2015. Budesonide for induction of remission in Crohn's disease. *Cochrane Database Syst Rev,* 2015**,** CD000296.

SANMARTÍ, R., TORNERO, J., NARVÁEZ, J., MUÑOZ, A., GARMENDIA, E., ORTIZ, A. M., ABAD, M. A., MOYA, P., MATEO, M. L., REINA, D., SALVATIERRA-OSSORIO, J., RODRIGUEZ, S., PALMOU-FONTANA, N., RUIBAL-ESCRIBANO, A. & CALVO-ALÉN, J. 2020. Efficacy and safety of glucocorticoids in rheumatoid arthritis: Systematic literature review. *Reumatol Clin (Engl Ed),* 16**,** 222-228.

SCHREIBER, S., DIGNASS, A., PEYRIN-BIROULET, L., HATHER, G., DEMUTH, D., MOSLI, M., CURTIS, R., KHALID, J. M. & LOFTUS, E. V., JR. 2018. Systematic review with meta-analysis: real-world effectiveness and safety of vedolizumab in patients with inflammatory bowel disease. *J Gastroenterol,* 53**,** 1048-1064.

SCIASCIA, S., FODDAI, S. G., ARBRILE, M., RADIN, M., CECCHI, I., BARINOTTI, A., FENOGLIO, R. & ROCCATELLO, D. 2024. Assessing the steroid-sparing effect of biological agents in randomized controlled trials for lupus: a scoping review. *Immunol Res,* 72**,** 538-553.

SCIASCIA, S., MOMPEAN, E., RADIN, M., ROCCATELLO, D. & CUADRADO, M. J. 2017. Rate of Adverse Effects of Medium- to High-Dose Glucocorticoid Therapy in Systemic Lupus Erythematosus: A Systematic Review of Randomized Control Trials. *Clin Drug Investig,* 37**,** 519-524.

SEBASTIAN, S., WILHELM, A., JESSICA, L., MYERS, S. & VEYSEY, M. 2019. Budesonide treatment for microscopic colitis: systematic review and meta-analysis. *Eur J Gastroenterol Hepatol,* 31**,** 919-927.

SHERLOCK, M. E., MACDONALD, J. K., GRIFFITHS, A. M., STEINHART, A. H. & SEOW, C. H. 2015. Oral budesonide for induction of remission in ulcerative colitis. *Cochrane Database Syst Rev,* 2015**,** CD007698.

SHIBER, S., STIEBEL-KALISH, H., SHIMON, I., GROSSMAN, A. & ROBENSHTOK, E. 2014. Glucocorticoid regimens for prevention of Graves' ophthalmopathy progression following radioiodine treatment: systematic review and meta-analysis. *Thyroid,* 24**,** 1515-23.

SHUAI, Z. Q., ZHANG, C. X., SHUAI, Z. W. & GE, S. L. 2021. Efficacy and safety of biological agents in the treatment of patients with Takayasu arteritis: a systematic review and meta-analysis. *Eur Rev Med Pharmacol Sci,* 25**,** 250-262.

SIEGELS, D., HERATIZADEH, A., ABRAHAM, S., BINNMYR, J., BROCKOW, K., IRVINE, AD., HALKEN, S., MORTZ, CG., FLOHR, C., SCHMID-GRENDELMEIER, P., VAN DER POEL, LA., MURARO, A., WEIDINGER, S., WERFEL, T., SCHMITT, J., 2021. Systemic treatments in the management of atopic dermatitis: A systematic review and meta-analysis. *Allergy,* 76(4):1053-1076

SINGH, J. A., HOSSAIN, A., KOTB, A. & WELLS, G. 2016. Risk of serious infections with immunosuppressive drugs and glucocorticoids for lupus nephritis: a systematic review and network meta-analysis. *BMC Med,* 14**,** 137.

SINGH, S., FACCIORUSSO, A., DULAI, P. S., JAIRATH, V. & SANDBORN, W. J. 2020. Comparative Risk of Serious Infections With Biologic and/or Immunosuppressive Therapy in Patients With Inflammatory Bowel Diseases: A Systematic Review and Meta-Analysis. *Clin Gastroenterol Hepatol,* 18**,** 69-81.e3.

SINGH, S., KIRTSCHIG, G., ANCHAN, V. N., CHI, C. C., TAGHIPOUR, K., BOYLE, R. J. & MURRELL, D. F. 2023. Interventions for bullous pemphigoid. *Cochrane Database Syst Rev,* 8**,** CD002292.

SIU, S., HARAOUI, B., BISSONNETTE, R., BESSETTE, L., ROUBILLE, C., RICHER, V., STARNINO, T., MCCOURT, C., MCFARLANE, A., FLEMING, P., KRAFT, J., LYNDE, C., GULLIVER, W., KEELING, S., DUTZ, J. & POPE, J. E. 2015. Meta-analysis of tumor necrosis factor inhibitors and glucocorticoids on bone density in rheumatoid arthritis and ankylosing spondylitis trials. *Arthritis Care Res (Hoboken),* 67**,** 754-64.

SUGIHARA, T., KAWAHITO, Y., KANEKO, Y., TANAKA, E., YANAI, R., YAJIMA, N., KOJIMA, M. & HARIGAI, M. 2024. Systematic review for the treatment of older rheumatoid arthritis patients informing the 2024 update of the Japan College of Rheumatology clinical practice guidelines for the management of rheumatoid arthritis. *Mod Rheumatol,* 34**,** 867-880.

TERZIROLI BERETTA-PICCOLI, B., MIELI-VERGANI, G. & VERGANI, D. 2017. Autoimmune hepatitis: Standard treatment and systematic review of alternative treatments. *World J Gastroenterol,* 23**,** 6030-6048.

TOME, J., TARIQ, R., HASSETT, L. C., KHANNA, S. & PARDI, D. S. 2024. Effectiveness and Safety Profile of Budesonide Maintenance in Microscopic Colitis: A Systematic Review and Meta-Analysis. *Inflamm Bowel Dis,* 30**,** 1178-1188.

TUNNICLIFFE, D. J., PALMER, S. C., HENDERSON, L., MASSON, P., CRAIG, J. C., TONG, A., SINGH-GREWAL, D., FLANC, R. S., ROBERTS, M. A., WEBSTER, A. C. & STRIPPOLI, G. F. 2018. Immunosuppressive treatment for proliferative lupus nephritis. *Cochrane Database Syst Rev,* 6**,** CD002922.

UGARTE-GIL, M. F., MAK, A., LEONG, J., DHARMADHIKARI, B., KOW, N. Y., REÁTEGUI-SOKOLOVA, C., ELERA-FITZCARRALD, C., ARANOW, C., ARNAUD, L., ASKANASE, A. D., BAE, S. C., BERNATSKY, S., BRUCE, I. N., BUYON, J., COSTEDOAT-CHALUMEAU, N., DOOLEY, M. A., FORTIN, P. R., GINZLER, E. M., GLADMAN, D. D., HANLY, J., INANC, M., ISENBERG, D., JACOBSEN, S., JAMES, J. A., JÖNSEN, A., KALUNIAN, K., KAMEN, D. L., LIM, S. S., MORAND, E., MOSCA, M., PESCHKEN, C., PONS-ESTEL, B. A., RAHMAN, A., RAMSEY-GOLDMAN, R., REYNOLDS, J., ROMERO-DIAZ, J., RUIZ-IRASTORZA, G., SÁNCHEZ-GUERRERO, J., SVENUNGSSON, E., UROWITZ, M., VINET, E., VAN VOLLENHOVEN, R. F., VOSKUYL, A., WALLACE, D. J., PETRI, M. A., MANZI, S., CLARKE, A. E., CHEUNG, M., FAREWELL, V. & ALARCON, G. S. 2021. Impact of glucocorticoids on the incidence of lupus-related major organ damage: a systematic literature review and meta-regression analysis of longitudinal observational studies. *Lupus Sci Med,* 8.

UNDELA, K., GOLDSMITH, L., KEW, K. M. & FERRARA, G. 2021. Macrolides versus placebo for chronic asthma. *Cochrane Database Syst Rev,* 11**,** CD002997.

URRUTICOECHEA-ARANA, A., COBO-IBÁÑEZ, T., VILLAVERDE-GARCÍA, V., SANTOS GÓMEZ, M., LOZA, E., VARGAS-OSORIO, K., FARIÑAS PADRÓN, L., DIAZ-GONZALEZ, F., CALVO-RÍO, V. & BLANCO, R. 2019. Efficacy and safety of biological therapy compared to synthetic immunomodulatory drugs or placebo in the treatment of Behçet's disease associated uveitis: a systematic review. *Rheumatol Int,* 39**,** 47-58.

VINCKEN, N. L. A., BALAK, D. M. W., KNULST, A. C., WELSING, P. M. J. & VAN LAAR, J. M. 2022. Systemic glucocorticoid use and the occurrence of flares in psoriatic arthritis and psoriasis: a systematic review. *Rheumatology (Oxford),* 61**,** 4232-4244.

VON GROOTE, T. C., WILLIAMS, G., AU, E. H., CHEN, Y., MATHEW, A. T., HODSON, E. M. & TUNNICLIFFE, D. J. 2021. Immunosuppressive treatment for primary membranous nephropathy in adults with nephrotic syndrome. *Cochrane Database Syst Rev,* 11**,** CD004293.

WANG, L., XI, J., ZHANG, S., WU, H., ZHOU, L., LU, J., ZHANG, T. & ZHAO, C. 2019. Effectiveness and safety of tacrolimus therapy for myasthenia gravis: A single arm meta-analysis. *J Clin Neurosci,* 63**,** 160-167.

WANG, Y., ZHAO, R., GU, Z., DONG, C., GUO, G. & LI, L. 2020. Effects of glucocorticoids on osteoporosis in rheumatoid arthritis: a systematic review and meta-analysis. *Osteoporos Int,* 31**,** 1401-1409.

WEBSTER, K. E., GALBRAITH, K., HARRINGTON-BENTON, N. A., JUDD, O., KASKI, D., MAARSINGH, O. R., MACKEITH, S., RAY, J., VAN VUGT, V. A. & BURTON, M. J. 2023. Systemic pharmacological interventions for Ménière's disease. *Cochrane Database Syst Rev,* 2**,** CD015171.

WILLIAMS, A. K., DOU, C. & CHEN, L. Y. C. 2021. Treatment of lymphocyte-variant hypereosinophilic syndrome (L-HES): what to consider after confirming the elusive diagnosis. *Br J Haematol,* 195**,** 669-680.

WINBLAD, L., LARSEN, C. G., HÅKANSSON, K., ABRAHAMSEN, B. & VON BUCHWALD, C. 2017. The risk of osteoporosis in oral steroid treatment for nasal polyposis: a systematic review. *Rhinology,* 55**,** 195-201.

WU, K., MICHALSKI, A., CORTES, D., ROZENBERG, D. & MATHUR, S. 2022. Glucocorticoid-induced myopathy in people with asthma: a systematic review. *J Asthma,* 59**,** 1396-1409.

XIE, W., YANG, X., JI, L. & ZHANG, Z. 2020. Incident diabetes associated with hydroxychloroquine, methotrexate, biologics and glucocorticoids in rheumatoid arthritis: A systematic review and meta-analysis. *Semin Arthritis Rheum,* 50**,** 598-607.

YUAN, A., WU, J. & HUANG, H. 2024. Comparison of treatment outcome between glucocorticoids and non-steroidal anti-inflammatory drugs in subacute thyroiditis patients-a systematic review and meta-analysis. *Front Endocrinol (Lausanne),* 15**,** 1384365.

ZHANG, C., WU, S. S., DONG, X. Q., WU, Z., ZHAO, H. & WANG, G. Q. 2019. The efficacy and safety of different doses of glucocorticoid for autoimmune hepatitis: A systematic review and meta-analysis. *Medicine (Baltimore),* 98**,** e18313.

ZHANG, D., XIA, B., ZHANG, X., LIANG, P. & HU, X. 2024. Efficacy and safety of low-dose corticosteroids combined with leflunomide for progressive IgA nephropathy: a systematic review and meta-analysis. *BMC Urol,* 24**,** 56.

ZHANG, J., DING, G., LI, J., LI, X., DING, L., YANG, S. & TANG, F. 2021. Risk Factors for Subacute Thyroiditis Recurrence: A Systematic Review and Meta-Analysis of Cohort Studies. *Front Endocrinol (Lausanne),* 12**,** 783439.

ZHANG, Q., MA, C., DONG, R., XIANG, W., LI, M., MA, Z. & YANG, Q. 2022. Efficacy and Safety of Anti-Tumor Necrosis Factor-Alpha Agents for Patients with Intestinal Behcet's Disease: A Systematic Review and Meta-Analysis. *Yonsei Med J,* 63**,** 148-157.

ZHANG, Q., ZHANG, W., LV, J., ZHANG, Z. & ZHAO, Y. 2024. The Effect of Local Steroid Administration on Idiopathic Granulomatous Mastitis: A Systematic Review andMeta-Analysis. *J Surg Res,* 295**,** 511-521.

ZHAO, C. Y. & MURRELL, D. F. 2015. Pemphigus vulgaris: an evidence-based treatment update. *Drugs,* 75**,** 271-84.

# Table S1: Differences between protocol and review

| **No.** | **Description** |
| --- | --- |
| 1 | In the protocol, the title indicated this was an ‘umbrella review’, as we restricted the included studies to evidence syntheses only, but the methodology proposed was a scoping review. As quality assessment is a feature of an umbrella review, but not a scoping review, and quality assessment was not conducted, we have referred to the design as a scoping review of reviews. |
| 2 | In the protocol, we aimed to include all chronic inflammatory conditions but as the review process unfolded, we rationalized that the following should be excluded: acute management of an infection or a flare, peri-operative use, uncomplicated urinary tract infections, dental implants and cancer. It was decided that these clinical situations did not meet our criteria for ‘chronic conditions’ and might confound our search to identify patterns of long term and/or high doses of oral steroid use. |
| 3 | In reviews where studies tested steroid versus steroid plus another agent, it was agreed that this may test the wrong comparator and therefore we excluded these designs unless steroid sparing was an outcome. |
| 4 | Data extractions forms were checked by an experienced systematic reviewer in discussion with the clinical lead rather than being resolved through group discussion, as it was decided that it would improve consistency throughout the process and throughput in a rapid review. |
| 5 | We had initially aimed to extract the number of included studies, however as the review was being conducted, we realized that most reviews only had a few studies within the scope of our review. The number in scope was very difficult, error-prone, and time-consuming to find in the context of a rapid review and therefore we reduced this to a binary ‘yes’ or ‘no’ for each of the study designs in our reporting. |

# Table S2: Details of included studies

| **Study ID** | **Geographical location** | **Care setting** | **Ethnicity reported?** | **Indication for glucocorticoids** | **Evidence on long-term use?** | **Evidence on use of high dose?** | **Evidence on steroid-sparing approaches?** | **Evidence on tapering regimens?** | **Adverse effects looked for/reported** |
| --- | --- | --- | --- | --- | --- | --- | --- | --- | --- |
| Agueda 2019 | Unspecified | Unspecified | No | giant cell arteritis and Takayasu arteritis | Yes (Takayasu arteritis only) | Yes | Yes | Yes |  |
| Akiyama 2021 | High income | Unspecified | No | eosinophilic granulomatosis with polyangiitis | Yes | Yes | Yes | Yes |  |
| Head 2016b | High income; Middle income | Secondary; Unspecified | No | chronic rhinosinusitis with nasal polyps | No | Yes | Yes | Yes | mood disturbances, gastrointestinal disturbances, insomnia |
| Gómez-Gómez 2017 | Unspecified | Unspecified | No | non-infectious anterior uveitis | Yes | No | Yes | Yes |  |
| Undela 2021 | High income | Unspecified | No | chronic asthma | Yes | Yes | Yes | Yes | none reported |
| von Groote 2021 | High income; Middle income | Primary | No | primary membranous nephropathy in nephrotic syndrome | Yes | Yes | Yes | Yes | adverse events |
| Natale 2020 | High income; Middle income | Secondary | No | IgA nephropathy | Yes | Yes | Yes | Yes | death, serious adverse events, infections, pulmonary TB, overt diabetes, bone marrow toxicity, gastrointestinal toxicity, hypotension, pneumonia, acute kidney injuries, ophthalmos-neuritis, osteonecrosis of the femoral head, ESKD, gastric perforation, newly diagnosed diabetes, impaired glucose tolerance, infections, hepatic dysfunction, gastrointestinal symptoms, cushing syndrome, acne, cramps, insomnia, alopecia, tremors, palpitations, gastritis, hypertension, recurrent UTI, cough, hyperkalaemia, arthralgia, striae, glucidic intolerance, herpes zoster, severe drug allergy, hepatotoxicity, upper respiratory infection, pulmonary infection, diarrhoea, herpes-zoster virus infection, pruritus, insomnia, alopecia, abnormal glucose metabolism, nasopharyngitis, acne, joint swelling, cushingoid, insomnia, diarrhoea, dyspepsia, headache, alopecia, back pain, mood swings, oedema peripheral, blood creatine phosphokinase increased, hirsutism, hypertension, muscle spasms, abdominal pain, nausea, upper respiratory tract infection, diverticulitis or appendicitis, pneumonia or respiratory tract infection, viral exanthema, knee empyema, death, malignant neoplasm, impaired glucose tolerance or DM, gastrointestinal bleeding, fracture, osteonecrosis, weight gain, respiratory infection, pneumocystis pneumonia, cryptococcal meningitis, nocardia infection of skin and knee joint, perianal abscess, urinary tract infection, fever, duodenal ulcer, gastrointestinal bleeding, gastric perforation, vascular necrosis, osteochondroma, pulmonary embolism, deep vein thrombosis, hepatotoxicity, haemoptysis, acute right upper quadrant pain, arthralgia, symptomatic incarcerated paraumbilical hernia, uremia, soft tissue injury, new-onset DM, vascular necrosis |
| Chande 2015 | High income; Unspecified | Secondary | No | Crohn's disease | Yes | Yes | Yes | Yes | whilst a number of adverse events were reported, none were clearly attributed to glucocorticoids |
| Iheozor-Ejiofor 2019 | High income | Secondary | No | Crohn's disease | Yes | No | Yes | Yes | Withdrawal due to adverse events, pancreatitis, leukopenia, nausea, infection, allergic reactions. |
| Antonio 2022 | High income | Secondary | Yes | giant cell arteritis | Yes | Yes | Yes | Yes | psychosis, immobilizing steroid-induced myopathy and hyperglycemia, lumbar fractures and vertebroplasty, and infection |
| Webster 2023 | Middle income | Other | No | Ménière's disease | Yes | Yes | Yes | Yes | steroid-related side effects |
| Williams 2021 | High income; Unspecified | Unspecified | No | lymphocyte-variant hypereosinophilic syndrome | Yes | Yes | Yes | Yes |  |
| Narula 2018 | High income | Unspecified | No | Crohn's disease | No | Yes | Yes | No | adverse events, withdrawals due to adverse events |
| Israel 2022 | High income; Middle income | Primary; Secondary; Other | No | severe asthma | Yes | Yes | Yes | Yes | none attributed to glucocorticoids |
| Guan 2024a | High income; Middle income | Unspecified | No | Behcet's uveitis | Yes | Yes | Yes | Yes |  |
| Zhang 2022 | High income; Middle income | Unspecified | No | intestinal Behçet’s disease | Yes | Yes | Yes | Yes | Systemic and local adverse effects, gastrointestinal rebleeding (page 149) |
| Giacomelli 2021 | Unspecified | Unspecified | No | active adult-onset Still’s Disease | Yes | Yes | Yes | Yes | non specifically attributed to steroids |
| Oon 2018 | Unspecified | Unspecified | Yes | SLE | Yes | Yes | Yes | Yes | unable to attribute to steroids |
| Wang 2019 | Unspecified | Unspecified | No | myasthenia gravis | Yes | No | Yes | Yes | Cushingoid syndrome |
| Singh 2020 | High income | Unspecified | No | inflammatory bowel disease | Yes | No | No | No | serious infections |
| Bleecker 2020 | High income; Unspecified | Primary; Secondary; Unspecified | No | asthma | Yes | Yes | No | No | gastroesophageal reflux, GI ulcers/bleeds, obesity, osteoporosis, fractures, infections, diabetes, cardiovascular complications, coronary heart disease, heart failure, hypertension, hypercholesterolemia, psychiatric conditions, anxiety, depression, cataracts, asthma-related bronchiectasis, chronic kidney disease, sleep disorders |
| Barba 2019 | High income; Unspecified | Unspecified | No | Idiopathic Inflammatory Myositis (IIM) and associated interstitial lung disease | Yes | Yes | Yes | No | adverse events related to combination therapies |
| Urruticoechea-Arana 2019 | Unspecified | Unspecified | No | Behçet’s disease-associated uveitis | Yes | Yes | Yes | Yes | not attributable to glucocorticoids |
| Head 2016c | High income | Secondary | No | chronic rhinosinusitis | No | Yes | Yes | Yes | gastrointestinal disturbances, skin irritation |
| Shuai 2021 | High income | Unspecified | No | Takayasu arteritis | Yes | Yes | Yes | Yes | none attributed to steroids |
| Osman 2014 | Unspecified | Unspecified | No | giant cell arteritis | Yes | No | Yes | Yes |  |
| Siu 2015 | Unspecified | Unspecified | No | rheumatoid arthritis | Yes | No | Yes | No | decreased bone mineral density (lumbar spine) |
| Cobo-Ibáñez 2014 | Unspecified | Unspecified | Yes | non-renal systemic lupus erythematosus | Yes | Yes | Yes | Yes | urinary tract infections, bacteraemia, infections in white matter, early infusion reaction and late infusion-related reaction |
| Liu 2022 | High income | Unspecified | No | primary sclerosing cholangitis | Yes | Yes | Yes | Yes | none that could be attributed to the relevant trials |
| Akiyama 2020 | High income | Secondary | No | Polymyalgia Rheumatica: Low-dose glucocorticoid therapy is standard but often associated with relapses and long-term complications. Tocilizumab (an anti-interleukin-6 receptor antibody) was studied as a steroid-sparing adjunct or alternative therapy. | Yes | No | Yes | Yes | Safety profile of Tocilizumab listed adverse effects including: leukopenia, neutropenia, dyslipidaemia, infections, skin and subcutaneous tissue disorders, infusion reactions, anaemia, oral herpes, upper respiratory infection, thrombocytopenia, phlegmone, light-headedness, epistaxis, knee osteoarthritis |
| Ming 2018 | High income; Unspecified | Unspecified | No | non-infectious uveitis | Yes | Yes | Yes | Yes | none attributable to steroids |
| Kwak 2022 | Unspecified | Unspecified | No | complex regional pain syndrome | No | Yes | Yes | Yes | elevated blood glucose, elevated blood pressure, sleep disturbance, acne, headache, weight gain, nausea, vomiting, osteopenia,  anxiety, indigestion |
| McMillan 2015 | High income; Middle income | Secondary; Unspecified | No | pemphigus vulgaris | Yes | Yes | Yes | Yes | gastric bleed, weight gain, gastrointestinal symptoms, cushingoid symptoms, DVT, depression, psychosis, osteoporosis, leukopenia, steroid glaucoma, infections |
| Chang 2019 | Unspecified | Unspecified | No | stable COPD | Yes | No | No | No | mortality, vertebral fracture risk |
| Schreiber 2018 | High income; Unspecified | Unspecified | No | inflammatory bowel disease | Yes | No | Yes | Yes | none attributed to steroids |
| Ji 2020 | High income | Unspecified | No | SLE | Yes | Yes | Yes | Yes |  |
| Chiang 2022 | Unspecified | Unspecified | No | systemic lupus erythematosus | Yes | No | Yes | No | withdrawals due to adverse events |
| Bitossi 2023 | Unspecified | Unspecified | No | non-infectious uveitis associated with systemic inflammatory diseases | Yes | Yes | Yes | Yes | not attributed to glucocorticoids |
| Raposo 2016 | High income; Middle income; Unspecified | Unspecified | No | systemic lupus erythematosus | Yes | Yes | No | No | HPV frequency |
| Hysa 2024 | Unspecified | Unspecified | No | polymyalgia rheumatica and giant cell arteritis | Yes | Yes | Yes | Yes | ocular complications, changes in bone mineral density |
| Bose 2022 | Unspecified | Unspecified | No | idiopathic membranous nephropathy (remission induction) | Yes | Yes | Yes | Yes | serious adverse events, discontinuation, serious infection |
| Chande 2016 | Unspecified | Secondary | No | Crohn's disease | Yes | Yes | Yes | Yes |  |
| Brito-Zerón 2016 | High income | Unspecified | No | IgG4-related disease | Yes | Yes | Yes | Yes | diabetes, amongst other side effects, non of which are attributed to glucocorticoids |
| Azukaitis 2022 | High income; Middle income; Unspecified | Other; Unspecified | No | minimal change disease in nephrotic syndrome | Yes | Yes | Yes | Yes | avascular necrosis, renal failure, doubling of serum creatinine, thromboses, gastrointestinal bleeding, diabetes/hyperglycaemia, obesity, Cushing's syndrome, hypertension, acute kidney injury, acne, mortality, new onset glucose intolerance, infections |
| Tunnicliffe 2018 | High income; Middle income | Unspecified | No | lupus nephritis | Yes | Yes | Yes | Yes | major infection, herpes zoster infection, GI disturbance |
| Arai 2018 | High income; Middle income; Unspecified | Unspecified | No | Primary newly diagnosed immune thrombocytopenia | No | Yes | Yes | No | hyperglycemia, vomiting, hypertension, gastrointestinal distress, petechia, pneumonia, haemorrhage, supraventricular tachycardia, myalgia, diabetes, fracture, hypokalaemia, atrial fibrilliation, fever, pain, dizziness, chest pain, cataract, vasculitis, neutropenia, anaphylaxis, myocardial infarction, pulmonary embolism, serum sickness, accidental fall |
| Jafarzadeh 2024 | Unspecified | Unspecified | No | vitiligo | Yes | Yes | Yes | No | acne, weight gain, dyspepsia, hyperglycemia, palpitations, striae, headache, insomnia, menstrual irregularity, hypertension, hirsutism, dyspepsia, peptic ulcer disease, pyoderma, gastrointestinal trouble,  increased appetite, flushing, lethargy |
| Amiche 2016 | Unspecified | Unspecified | No | miscellaneous chronic inflammatory and autoimmune conditions | Yes | Yes | No | No | vertebral fractures, non-vertebral fractures, decreased bone mineral density |
| Lin 2023 | High income | Unspecified | No | pemphigoid | Yes | Yes | Yes | Yes | total number of adverse events |
| Ugarte-Gil 2021 | High income; Middle income; Unspecified | Unspecified | No | SLE | Yes | Yes | No | No | overall organ damage, cataract, cerebrovascular accidents, myocardial infarction, overall cardiovascular events, avascular necrosis, osteonecrosis, osteoporotic fractures |
| Chen 2024 | High income; Middle income | Unspecified | No | allergic bronchopulmonary aspergillosis | Yes | Yes | Yes | Yes | none attributed to glucocorticoids |
| Guan 2024b | Middle income | Unspecified | Yes | severe alopecia areata | Yes | No | Yes | No | overall adverse events, facial mooning, weight gain, increased appetite and gastritis |
| Allen 2016 | High income | Unspecified | No | rheumatological, respiratory, nephrological, gastrointestinal, haematological, dermatological and neurological systemic inflammatory diseases | Yes | Yes | No | No | bone mineral density, vertebral fractures, non vertebral factures, musculoskeletal effects, serious adverse events, deaths, withdrawals due to adverse events |
| Zhang 2024a | High income | Secondary | No | progressive IgA nephropathy | Yes | Yes | Yes | No | respiratory infection, abnormal liver function, diarrhoea, alopecia, UTI, herpes zoster, serious adverse event, pruritis, insomnia, pneumonia, diabetes |
| Phinyo 2024 | Unspecified | Unspecified | No | oral glucocorticoid-dependent asthma | Yes | Yes | Yes | Yes | not attributed to steroids |
| Sugihara 2024 | High income; Unspecified | Primary; Unspecified | No | RA | Yes | Yes | Yes | No | serious adverse events, serious infectious events, fractures, hypertension, diabetes, cardiovascular events, cataracts, glaucoma |
| Zhao 2015 | Unspecified | Unspecified | No | pemphigus vulgaris | Yes | Yes | Yes | Yes | adverse events |
| Pitre 2023 | High income; Middle income | Primary; Secondary | No | fibrotic interstitial lung disease | Yes | Yes | No | No | none reported |
| Goswami 2022 | Unspecified | Unspecified | No | idiopathic inflammatory myopathy | Yes | Yes | Yes | Yes |  |
| Molooghi 2022 | High income; Middle income | Unspecified | No | SLE | No | Yes | Yes | No | serious CNS infections |
| Dai 2020 | Unspecified | Unspecified | No | Takayasu arteritis-induced renal arteritis | Yes | Yes | Yes | Yes | none reported specifically for glucocorticoids |
| Paglia 2021 | Unspecified | Unspecified | No | RA | Yes | Yes | Yes | Yes | not clearly attributed to the high dose/long-term use of steroids |
| Ozguler 2018 | Unspecified | Unspecified | No | Behçet’s syndrome with major organ involvement | Yes | Yes | Yes | Yes | none clearly attributed to steroids |
| Yuan 2024 | High income; Middle income | Unspecified | No | subacute thyroiditis | No | Yes | Yes | No | permanent hypothyroidism |
| Partridge 2018 | High income; Middle income | Secondary; Unspecified | No | pyoderma gangrenosum | Yes | Yes | Yes | No | From RCT with clear high-dose glucocorticoid use: bowel perforation, serious infections, other infections. From observational studies: hyperglycaemia, hypertension, overweight, acne, intracranial haemorrhage. Other AE were not clearly associated with glucocorticoid treatment. |
| Winblad 2017 | High income | Unspecified | Yes | chronic rhinosinusitis with nasal polyps | Yes | Yes | No | No | osteoporosis |
| Blavnsfeldt 2018 | High income | Unspecified | No | rheumatoid arthritis | Yes | No | Yes | Yes | bone mineral density at lumbar spine and hip, risk of vertebral fractures, bone turnover |
| Durazzo 2019 | High income | Unspecified | Yes | autoimmune hepatitis | No | Yes | Yes | Yes | osteoporosis, diabetes, myopathy, Cushingoid facies, psychotic episodes |
| Etchegaray-Morales 2024 | High income; Middle income | Primary; Secondary; Unspecified | Yes | systemic lupus erythematosus | No | Yes | No | No | diabetes |
| Zhang 2019 | High income; Unspecified | Unspecified | No | autoimmune hepatitis | No | Yes | No | No | glucocorticoid-related adverse effects |
| Xie 2020 | High income; Middle income | Unspecified | Yes | RA | No | Yes | No | No | diabetes |
| Ji 2022 | High income; Middle income | Unspecified | No | SLE | Yes | No | Yes | Yes |  |
| Bonovas 2018 | Unspecified | Unspecified | No | Crohn’s disease and ulcerative colitis | Yes | Yes | Yes | Yes | total number of treatment discontinuations/withdrawals, any serious AEs, and corticosteroid-related AEs (sum of number of reported instances of moon face, buffalo hump, acne, hirsutism, purple skin striae, easy bruising, ankle swelling, hair loss, mood swings, depression, sleep changes, insomnia) |
| Sherlock 2015 | High income | Unspecified | No | ulcerative colitis | No | Yes | Yes | Yes | adrenal suppression, Cushing's-like syndrome, gastrointestinal adverse events, weight gain |
| Lin 2018 | Unspecified | Secondary | No | IgA nephropathy | Yes | Yes | Yes | Yes | overall adverse events, infections, death, hypertension, cough, insomnia, gastrointestinal bleeding, increased appetite, heartburn, perspiration, palpitations, headache, diabetes mellitus, and cushingoid features |
| Head 2016a | Middle income | Secondary | No | Chronic rhinosinusitis | No | Yes | Yes | Yes | none reported |
| Liu 2021 | High income; Unspecified | Other | No | primary autoimmune haemolytic anaemia | Yes | Yes | Yes | Yes | serious adverse events and cumulative adverse events (including fever, nausea, dyspnea, dyspepsia, restless legs, vertigo, increased appetite, insomnia, arthralgia, headache, fatigue, hypotension, palpitation, hyperglycaemia, pneumonia, pulmonary abscess, diverticulitis, prostatitis, neutropenia, pulmonary embolism, spontaneous vertebral fractures, intracranial tumour, septic shock post-colonic perforation, septic and cardiogenic shock post-amputation) |
| Palmowski 2024 | High income; Unspecified | Unspecified | No | RA and SLE with low disease activity | Yes | No | Yes | Yes | adverse events, serious adverse events, withdrawals due to adverse events, adrenal insufficiency |
| Rezaie 2015 | High income; Unspecified | Unspecified | No | active Crohn's disease | Yes | Yes | Yes | Yes | withdrawal due to adverse events, glucocorticoid-associated adverse events, adverse events, adrenal insufficiency, moon facies, acne |
| Omar 2020 | High income; Middle income | Unspecified | No | IgG4-related disease | Yes | Yes | Yes | Yes | adverse effects |
| Abe 2019 | High income | Secondary | No | Glucocorticoids were assessed for improving platelet count and remission in APAT (antiphospholipid antibody associated thrombocytopenia) patients. | Yes | Yes | Yes | No | thrombosis, hemorrhage, infections, mortality, and relapse rates. |
| Hmamouchi 2023 | Middle income | Unspecified | No | rheumatoid arthritis and systemic lupus erythematosus | Yes | Yes (scleroderma only) | No | No | fracture risk, osteopenia, osteoporosis |
| Fujiwara 2019 | High income | Secondary | No | Bell’s palsy | No | Yes | Yes | Yes | sleep disturbances, constipation, hiccups, elevated liver enzymes, faecal occult blood, and steroid withdrawal syndrome |
| Singh 2016 | High income; Middle income; Unspecified | Secondary; Unspecified | No | lupus nephritis | Yes | Yes | Yes | Yes | serious infection |
| Chavez-Alvarez 2021 | Middle income | Unspecified | No | vitiligo | Yes | No | Yes | Yes | weight gain |
| Zhang 2021 | High income; Middle income | Unspecified | No | subacute thyroiditis | No | Yes | Yes | Yes |  |
| Sciascia 2017 | High income | Unspecified | No | systemic lupus erythematosus | Yes | Yes | Yes | Yes | infections, hyperglycemia, aseptic necrosis, hypertension, Cushingoid habitus, hypokalemia |
| Black 2016 | High income; Unspecified | Unspecified | No | rheumatoid arthritis | Yes | Yes | No | No | cataracts, glaucoma |
| Vincken 2022 | Unspecified | Primary; Secondary; Other | No | psoriatic arthritis and psoriasis | Yes | Yes | Yes | Yes |  |
| Shiber 2014 | Unspecified | Unspecified | No | Graves' ophthalmopathy | Yes | Yes | Yes | Yes | Cushingoid features, weight gain, anxiety, hyperglycemia, insomnia |
| Figueroa-Parra 2024 | High income; Middle income; Unspecified | Unspecified | Yes | lupus nephritis | Yes | Yes | Yes | Yes | serious infections, mortality |
| Al Efraij 2019 | High income; Middle income | Primary; Other; Unspecified | No | asthma | Yes | Yes | No | No | any complications, infections, gastrointestinal complications, psychiatric complications, cardiovascular complications, metabolic complications, bone and muscle complications, ocular complications, peptic ulcer, hypertension, diabetes, cataracts, infections, fractures, osteoporosis, glaucoma |
| Wang 2020 | High income; Middle income | Unspecified | No | RA | Yes | No | No | No |  |
| Sciascia 2024 | Unspecified | Unspecified | Yes | systemic lupus erythematosus | Yes | Yes | Yes | Yes | none reported |
| Li 2021 | Unspecified | Unspecified | No | adrenal insufficiency | No | Yes | No | No | overall fractures, osteoporotic fractures |
| Joseph 2016 | Unspecified | Unspecified | No | miscellaneous | Yes | Yes | Yes | Yes | adrenal insufficiency |
| Madhok 2016 | Unspecified | Primary; Secondary | No | Bell's Palsy | No | Yes | Yes | Yes | adverse effects, sleep disturbances |
| D'Haens 2016 | High income | Unspecified | No | ulcerative colitis | Yes | Yes | Yes | Yes | weight gain, pyogenic complications, eye complications, moon face, acne, facial flushing, blood pressure effects/palpitations, dyspepsia, confusion, hair growth, hyperglycemia, suppression of cortisol, oedema (p3), gastrointestinal disturbances, sleep changes (p4), menorrhagia (p6), headache, infections (p7), nausea (p9). The following were also tested/looked for: haemoglobin, heart rate, platelet count, ESR, C-reactive protein, orosomucoids, serum albumin, glycosuria, bone formation, bone mineral density (p4/5/6) |
| Kang 2023 | High income; Middle income | Secondary; Other; Unspecified | No | Takayasu arteritis | Yes | Yes | Yes | Yes | not attributed to steroids in in scope studies |
| Elsouri 2023 | High income | Secondary; Unspecified | No | rheumatoid arthitis | Yes | Yes | Yes | No | pneumonia |
| Alchi 2023 | High income; Unspecified | Unspecified | No | Antineutrophil cytoplasmic antibody-associated vasculitis (AAV) | Yes | Yes | Yes | Yes | infections, diabetes, death or end stage kidney disease |
| Moja 2015 | High income; Middle income | Unspecified | No | active mild to moderate Crohn’s disease (induction and maintenance of remission) | Yes | Yes | Yes | No | withdrawals due to adverse events reported |
| Tome 2024 | High income; Unspecified | Unspecified | No | microscopic colitis | Yes | Yes | No | No | metabolic bone disease, hypertension, hyperglycaemia, cataracts, glaucoma, nausea, headache, abdominal discomfort, dyspepsia, oedema |
| Chalitsios 2021 | High income; Middle income | Unspecified | No | asthma (adults) | Yes | Yes | No | No | osteoporosis, fracture |
| Kafil 2017 | High income; Unspecified | Secondary; Other; Unspecified | No | collagenous colitis | Yes | Yes | Yes | Yes | adverse events, withdrawals due to adverse events, serious adverse events. Those specifically noted related to glucocorticoids were nausea, headache, increase in body weight, disturbed sleep, upper abdominal discomfort, leg cramps |
| Manguso 2016 | High income; Unspecified | Unspecified | No | mild to moderate ulcerative colitis | No | Yes | Yes | No | adverse events |
| Hughes 2017 | High income; Unspecified | Secondary | No | chronic inflammatory demyelinating polyradiculoneuropathy | Yes | Yes | Yes | Yes | sleeplessness, cushingoid facies, weight gain, diabetes mellitus, hypertension, osteopenia, glaucoma, hyperglycaemia |
| Campbell 2020 | High income | Unspecified | No | giant cell arteritis and polymyalgia rheumatica | Yes (giant cell arteritis only) | Yes | Yes | Yes | steroid-induced diabetes, urinary tract infection, lower respiratory tract infection, vertebral fractures, sleep disturbances, hyperglycemia, moderate hyperlipidemia, bronchitis, influenza |
| Zhang 2024b | Middle income | Unspecified | Yes | idiopathic granulomatous mastitis | Yes | Yes | Yes | Yes | side effects |
| Atzmony 2015 | High income; Middle income | Secondary; Unspecified | No | pemphigus vulgaris and pemphigus foliaceus | Yes | Yes | Yes | Yes | withdrawal due to adverse events, all-cause mortality |
| Atzmony et al., 2014 | High income; Middle income | Unspecified | No | pemphigus vulgaris and pemphigus foliaceus | Yes | Yes | Yes | Yes | withdrawals due to adverse events, mortality, infections, sepsis, hepatitis |
| Edel 2020 | High income; Middle income | Unspecified | No | various | Yes | Yes | Yes | Yes | mortality, adverse events, serious adverse events, cardiac related events, infections, gastrointestinal, psychiatric, hyperglycaemia, hypertension, oedema |
| Lattanzi 2017 | High income | Unspecified | No | relapses of multiple sclerosis | No | Yes | Yes | Yes | Insomnia |
| Sebastian 2019 | Unspecified | Unspecified | No | microscopic colitis | Yes | Yes | Yes | Yes | headache |
| Kulkarni 2022 | Unspecified | Unspecified | No | miscellaneous | Other: Yes (>1 month threshold) | Yes | Yes | Yes | hyperglycaemia, hypertension, weight gain, hyperlipidaemia |
| Atzmony 2016 | High income; Middle income | Unspecified | No | cutaneous lichen planus | No | Yes | Yes | No | anaemia, oedema, dyspepsia, acne, moon face, striae, and menstrual abnormalities |
| Sanmartí 2020 | Unspecified | Unspecified | No | RA | Yes | Yes | Yes | Yes |  |
| Palmowski 2023 | High income; Unspecified | Unspecified | No | RA | Yes | No | No | No | any adverse events, infections |
| Prado 2023 | Unspecified | Unspecified | No | myasthenia gravis | Yes | No | Yes | Yes | unable to attribute to steroids |
| Koning 2024 | High income; Unspecified | Unspecified | No | miscellaneous | Yes | Yes | Yes | Yes | neuropsychiatric adverse effects, such as depression and mania |
| De Bock 2024 | Unspecified | Unspecified | No | various (SLE, neoplastic disorders, ITP, asthma, ocular disease, nephrotic syndrome, RA, 'rheumatic illness', multiple myeloma, inflammatory bowel disease) | No | Yes | Yes | Yes | mania, hypomania, psychotic disorder, paranoid psychosis |
| Dhir 2021 | High income; Middle income | Unspecified | No | spondyloarthritis | Yes | Yes | Yes | Yes | fluid retention, dyspepsia, facial puffiness, acne, weight gain |
| Singh 2023 | High income | Secondary | No | bullous pemphigoid | Yes | Yes | Yes | Yes | infection, low white cell count, organ impairment, cardiovascular, total AEs, severe AEs, mortality (sepsis), diabetes, hyperglycaemia |
| Oaklander 2017 | Unspecified | Unspecified | No | chronic inflammatory demyelinating polyradiculoneuropathy | Yes | Yes | Yes | Yes | sleep disturbance, moon facies, diabetes mellitus, hypertension, weight gain, osteopoenia, glaucoma, cardiac death, arrhythmia, hyperglycaemia |
| Mithoowani 2016 | Middle income | Unspecified | No | immune thrombocytopenia | Yes | No | Yes | Yes | weight gain, cushingoid appearance, gastrointestinal symptoms (e.g., peptic ulcer, nausea, diarrhoea), hyperglycaemia, insomnia, infection, pneumonia, vomiting, hypertension, fatigue, oedema, anxiety, mood disorders, dizziness, acne, myalgia, arthralgia, palpitations, fever, elevated liver enzymes, total adverse events |
| Bergstra 2023 | High income; Unspecified | Primary; Secondary; Other; Unspecified | No | rheumatoid arthritis | Yes | Yes | Yes | Yes | osteoporosis, osteoporotic fractures, serious infections, diabetes, mortality, adverse pregnancy, cardiovascular outcomes, glaucoma |
| Kolkhir 2019 | High income | Unspecified | No | urticarial vasculitis | Yes | Yes | Yes | Yes | not attributed to steroids in observational studies |
| Mateos-Haro 2023 | High income; Middle income | Secondary | No | alopecia | Yes | Yes | Yes | No | adverse events |
| Buttgereit 2016 | Unspecified | Unspecified | No | polymyalgia rheumatica and giant cell arteritis | Yes | Yes | Yes | Yes | weight gain, hypertension, diabetes, cushingoid habitus, fractures, serious infection, oral ulceration, hair loss |
| Siegels 2021 | High income; Middle | Unspecified | No | Atopic dermatitis | No | Yes | Yes | Yes | adverse events, serious adverse events, withdrawals due to adverse events |
| Terziroli Beretta-Piccoli 2017 | High income; Unspecified | Unspecified | No | autoimmune hepatitis | No | Yes | Yes | Yes | none in systematic review section of paper |

# Table S3: Studies by indication

| **Indication** | **Speciality** | **n** | **Studies** |
| --- | --- | --- | --- |
| Behcet's disease | rheumatology (other) | 4 | Ozguler 2018; Urruticoechea-Arana 2019; Zhang 2022; Guan 2024a |
| IgG4-related disease | rheumatology (other) | 2 | Omar 2020; Brito-Zerón 2016 |
| Still's disease | rheumatology (other) | 1 | Giacomelli 2021 |
| idiopathic inflammatory myopathy | rheumatology (other) | 1 | Goswami 2022 |
| scleroderma | rheumatology (other) | \|  \| 1 \| \| --- \| --- \| | Hmamouchi 2023 |
| giant cell arteritis | rheumatology (GCA/PMR) | 6 | Buttgereit 2016; Campbell 2020; Hysa 2024; Osman 2014; Antonio 2022; Agueda 2019 |
| polymyalgia rheumatica | rheumatology (GCA/PMR) | 5 | Buttgereit 2016; Campbell 2020; Hysa 2024; Akiyama 2020; Floris 2022 |
| rheumatoid arthritis | rheumatology (other) | 13 | Bergstra 2023; Palmowski 2023; SanmartÃ­ 2020; Elsouri 2023; Wang 2020; Black 2016; Hmamouchi 2023; Palmowski 2024; Xie 2020; Blavnsfeldt 2018; Paglia 2021; Sugihara 2024; Siu 2015 |
| spondyloarthritis | rheumatology (other) | 1 | Dhir 2021 |
| systemic lupus erythematosus | rheumatology (other) | 15 | Sciascia 2024; Figueroa-Parra 2024; Sciascia 2017; Singh 2016; Ji 2022; Etchegaray-Morales 2024; Molooghi 2022; Ugarte-Gil 2021; Tunnicliffe 2018; Raposo 2016; Chiang 2022; Ji 2020; Cobo-IbÃ¡Ã±ez 2014; Oon 2018; Ji 2021 |
| small vessel vasculitis | rheumatology (other) | 3 | Kolkhir 2019; Alchi 2023; Akiyama 2021 |
| Takayasu arteritis | rheumatology (other) | 5 | Kang 2023; Dai 2020; Shuai 2021; Agueda 2019; Misra 2021 |
| allergic bronchopulmonary aspergillosis | respiratory | 1 | Chen 2024 |
| asthma | respiratory | 7 | Chalitsios 2021; Al Efraij 2019; Phinyo 2024; Bleecker 2020; Israel 2022; Undela 2021; Wu 2022 |
| chronic obstructive pulmonary disease | respiratory | 1 | Chang 2019 |
| chronic rhinosinusitis with nasal polyposis | respiratory | 4 | Head 2016a; Winblad 2017; Head 2016c; Head 2016b |
| fibrotic lung disease | respiratory | 1 | Pitre 2023 |
| miscellaneous respiratory | respiratory | 1 | Breakey 2016 |
| myositis associated interstitial lung disease | respiratory | 1 | Barba 2019 |
| IgA nephropathy | renal | 3 | Lin 2018; Zhang 2024a; Natale 2020 |
| idiopathic membranous nephropathy | renal | 1 | Bose 2022 |
| nephrotic syndrome | renal | 1 | Azukaitis 2022 |
| primary membranous nephropathy | renal | 1 | von Groote 2021 |
| mastitis | other | 1 | Zhang 2024b |
| non-infectious uveitis | ophthalmology | 3 | Bitossi 2023; Ming 2018; Gómez-Gómez 2017 |
| Bell's palsy | neurology | 2 | Madhok 2016; Fujiwara 2019 |
| chronic inflammatory demyelinating polyradiculoneuropathy | neurology | 2 | Oaklander 2017; Hughes 2017 |
| multiple sclerosis | neurology | 1 | Lattanzi 2017 |
| myasthenia gravis | neurology | 2 | Prado 2023; Wang 2019 |
| pain syndrome | neurology | 1 | Kwak 2022 |
| miscellaneous | miscellaneous | 9 | De Bock 2024; Koning 2024; Kulkarni 2022; Edel 2020; Joseph 2016; Vincken 2022; Allen 2016; Amiche 2016; Broersen 2015 |
| lymphocyte-variant hypereosinophilic syndrome | immunology | 1 | Williams 2021 |
| haemolytic anaemia | haematology | 1 | Liu 2021 |
| thrombocytopenia | haematology | 3 | Mithoowani 2016; Abe 2019; Arai 2018 |
| Graves' ophthalmopathy | endocrinology | 1 | Shiber 2014 |
| adrenal insufficiency | endocrinology | 1 | Li 2021 |
| subacute thyroiditis | endocrinology | 2 | Zhang 2021; Yuan 2024 |
| alopecia | dermatology | 2 | Mateos-Haro 2023; Guan 2024b |
| blistering conditions | dermatology | 6 | Singh 2023; Atzmony 2014; Atzmony 2015; Zhao 2015; Lin 2023; McMillan 2015 |
| cutaneous lichen planus | dermatology | 1 | Atzmony 2016 |
| pyoderma gangrenosum | dermatology | 1 | Partridge 2018 |
| vitiligo | dermatology | 2 | Chavez-Alvarez 2021; Jafarzadeh 2024 |
| eczema | dermatology | 1 | Siegels 2021 |
| autoimmune hepatitis | GI | 3 | Terziroli Beretta-Piccoli 2017; Zhang 2019; Durazzo 2019 |
| inflammatory bowel disease | GI | 15 | Sebastian 2019; Manguso 2016; Kafil 2017; Tome 2024; Moja 2015; D'Haens 2016; Rezaie 2015; Sherlock 2015; Bonovas 2018; Chande 2016; Schreiber 2018; Singh 2020; Narula 2018; Iheozor-Ejiofor 2019; Chande 2015 |
| primary sclerosing cholangitis | GI | 1 | Liu 2022 |
| Meniere's disease | ENT | 1 | Webster 2023 |

# Table S4: Summary of adverse events

| **Simplified_AE** | **AE type** | **n** | **Extracted descriptions** | **Studies** |
| --- | --- | --- | --- | --- |
| cancer | cancer | 2 | malignant neoplasm; intracranial tumour | Natale 2020; Liu 2021 |
| osteochondroma | cancer | 1 | osteochondroma | Natale 2020 |
| change in blood pressure | cardiovascular | 20 | hypotension; hypertension; blood pressure effects; elevated blood pressure | Liu 2021; Natale 2020; Al Efraij 2019; Arai 2018; Azukaitis 2022; Bleecker 2020; Buttgereit 2016; Edel 2020; Jafarzadeh 2024; Kulkarni 2022; Lin 2018; Mithoowani 2016; Oaklander 2017; Partridge 2018; Sciascia 2017; Tome 2024; D'Haens 2016; Hughes 2017; Sugihara 2024; Kwak 2022 |
| cardiovascular event | cardiovascular | 8 | overall cardiovascular events; cardiovascular outcomes; cardiovascular events; cardiovascular complications; cardiovascular; cardiac related events; cardiac death | Ugarte-Gil 2021; Bergstra 2023; Sugihara 2024; Al Efraij 2019; Bleecker 2020; Singh 2023; Edel 2020; Oaklander 2017 |
| oedema | cardiovascular | 6 | oedema peripheral; oedema (p3); oedema; edema | Natale 2020; D'Haens 2016; Atzmony 2016; Mithoowani 2016; Tome 2024; Edel 2020 |
| palpitations | cardiovascular | 6 | palpitations; palpitation | D'Haens 2016; Jafarzadeh 2024; Lin 2018; Mithoowani 2016; Natale 2020; Liu 2021 |
| thrombosis | cardiovascular | 6 | thromboses; pulmonary embolism; deep vein thrombosis; Thrombosis; DVT | Azukaitis 2022; Arai 2018; Liu 2021; Natale 2020; Abe 2019; McMillan 2015 |
| dizziness | cardiovascular | 3 | light-headedness; dizziness | Akiyama 2020; Arai 2018; Mithoowani 2016 |
| arrhythmia | cardiovascular | 2 | atrial fibrilliation; arrhythmia | Arai 2018; Oaklander 2017 |
| haemorrhage | cardiovascular | 2 | hemorrhage; haemorrhage | Abe 2019; Arai 2018 |
| myocardial infarction | cardiovascular | 2 | myocardial infarction | Arai 2018; Ugarte-Gil 2021 |
| abnormal heart rate | cardiovascular | 1 | heart rate | D'Haens 2016 |
| cerebrovascular accident | cardiovascular | 1 | cerebrovascular accidents | Ugarte-Gil 2021 |
| chest pain | cardiovascular | 1 | chest pain | Arai 2018 |
| coronary heart disease | cardiovascular | 1 | coronary heart disease | Bleecker 2020 |
| heart failure | cardiovascular | 1 | heart failure | Bleecker 2020 |
| intracranial haemorrhage | cardiovascular | 1 | intracranial haemorrhage. Other AE were not clearly associated with glucocorticoid treatment. | Partridge 2018 |
| supraventricular tachycardia | cardiovascular | 1 | supraventricular tachycardia | Arai 2018 |
| acne | dermatological | 11 | acne | Atzmony 2016; Azukaitis 2022; Bonovas 2018; D'Haens 2016; Dhir 2021; Jafarzadeh 2024; Kwak 2022; Mithoowani 2016; Natale 2020; Partridge 2018; Rezaie 2015 |
| alopecia | dermatological | 4 | hair loss; alopecia | Bonovas 2018; Buttgereit 2016; Natale 2020; Zhang 2024a |
| striae | dermatological | 4 | striae; purple skin striae | Atzmony 2016; Jafarzadeh 2024; Natale 2020; Bonovas 2018 |
| pruritus | dermatological | 2 | pruritus; pruritis | Natale 2020; Zhang 2024a |
| easy bruising | dermatological | 1 | easy bruising | Bonovas 2018 |
| hair growth | dermatological | 1 | hair growth | D'Haens 2016 |
| perspiration | dermatological | 1 | perspiration | Lin 2018 |
| petechiae | dermatological | 1 | petechia | Arai 2018 |
| skin and subcutaneous tissue disorder | dermatological | 1 | subcutaneous tissue disorders | Akiyama 2020 |
| skin irritation | dermatological | 1 | skin irritation | Head 2016c |
| diabetes | endocrine | 20 | steroid-induced diabetes; overt diabetes; new-onset DM; newly diagnosed diabetes; diabetes mellitus; diabetes; DM | Campbell 2020; Natale 2020; Hughes 2017; Lin 2018; Oaklander 2017; Al Efraij 2019; Alchi 2023; Arai 2018; Azukaitis 2022; Bergstra 2023; Bleecker 2020; Brito-Zerón 2016; Buttgereit 2016; Durazzo 2019; Etchegaray-Morales 2024; Ji 2021; Singh 2023; Sugihara 2024; Xie 2020; Zhang 2024a |
| hyperglycaemia | endocrine | 19 | hyperglycemia; hyperglycaemia; elevated blood glucose | Antonio 2022; Arai 2018; Campbell 2020; D'Haens 2016; Jafarzadeh 2024; Sciascia 2017; Azukaitis 2022; Breakey 2016; Edel 2020; Kulkarni 2022; Liu 2021; Mithoowani 2016; Oaklander 2017; Partridge 2018; Singh 2023; Tome 2024; Kwak 2022; Shiber 2014; Hughes 2017 |
| weight gain | endocrine | 19 | weight gain; overweight; obesity; increase in body weight | Buttgereit 2016; Chavez-Alvarez 2021; D'Haens 2016; Dhir 2021; Guan 2024b; Hughes 2017; Jafarzadeh 2024; Kulkarni 2022; McMillan 2015; Mithoowani 2016; Natale 2020; Oaklander 2017; Sherlock 2015; Partridge 2018; Azukaitis 2022; Bleecker 2020; Kafil 2017; Kwak 2022; Shiber 2014 |
| Cushing's syndrome | endocrine | 12 | cushingoid symptoms; cushingoid habitus; cushingoid facies; cushingoid appearance; cushingoid; cushing syndrome; and cushingoid features; Cushingoid syndrome; Cushingoid habitus; Cushingoid features; Cushingoid facies; Cushing's-like syndrome; Cushing's syndrome | McMillan 2015; Buttgereit 2016; Hughes 2017; Mithoowani 2016; Natale 2020; Lin 2018; Wang 2019; Sciascia 2017; Shiber 2014; Durazzo 2019; Sherlock 2015; Azukaitis 2022 |
| adrenal insufficiency | endocrine | 6 | suppression of cortisol; adrenal suppression; adrenal insufficiency | D'Haens 2016; Sherlock 2015; Broersen 2015; Joseph 2016; Palmowski 2024; Rezaie 2015 |
| moon face | endocrine | 6 | moon facies; moon face; facial puffiness; facial mooning | Oaklander 2017; Rezaie 2015; Atzmony 2016; D'Haens 2016; Dhir 2021; Guan 2024b |
| increased appetite | endocrine | 4 | increased appetite | Guan 2024b; Lin 2018; Liu 2021; Jafarzadeh 2024 |
| hirsutism | endocrine | 3 | hirsutism | Bonovas 2018; Jafarzadeh 2024; Natale 2020 |
| hypokalaemia | endocrine | 3 | hypokalemia; hypokalaemia; hyperkalaemia | Sciascia 2017; Arai 2018; Natale 2020 |
| glucose intolerance | endocrine | 2 | new onset glucose intolerance; impaired glucose tolerance; glucidic intolerance | Azukaitis 2022; Natale 2020 |
| hyperlipidaemia | endocrine | 2 | moderate hyperlipidemia; hyperlipidaemia | Campbell 2020; Kulkarni 2022 |
| abnormal glucose metabolism | endocrine | 1 | abnormal glucose metabolism | Natale 2020 |
| buffalo hump | endocrine | 1 | buffalo hump | Bonovas 2018 |
| dyslipidaemia | endocrine | 1 | dyslipidaemia | Akiyama 2020 |
| fluid retention | endocrine | 1 | fluid retention | Dhir 2021 |
| glycosuria | endocrine | 1 | glycosuria | D'Haens 2016 |
| hypercholesterolemia | endocrine | 1 | hypercholesterolemia | Bleecker 2020 |
| hypothyroidism | endocrine | 1 | permanent hypothyroidism | Yuan 2024 |
| metabolic event | endocrine | 1 | metabolic complications | Al Efraij 2019 |
| steroid withdrawal syndrome | endocrine | 1 | and steroid withdrawal syndrome | Fujiwara 2019 |
| dyspepsia | gastrointestinal | 8 | indigestion; dyspepsia | Kwak 2022; Atzmony 2016; D'Haens 2016; Dhir 2021; Jafarzadeh 2024; Liu 2021; Natale 2020; Tome 2024 |
| gastrointestinal adverse event | gastrointestinal | 7 | gastrointestinal trouble; gastrointestinal toxicity; gastrointestinal symptoms (e.g.; gastrointestinal symptoms; gastrointestinal complications; gastrointestinal adverse events; gastrointestinal | Jafarzadeh 2024; Natale 2020; Mithoowani 2016; McMillan 2015; Al Efraij 2019; Sherlock 2015; Edel 2020 |
| nausea | gastrointestinal | 7 | nausea (p9). The following were also tested; nausea; nausea | D'Haens 2016; Liu 2021; Mithoowani 2016; Natale 2020; Tome 2024; Iheozor-Ejiofor 2019; Kwak 2022 |
| gastrointestinal bleeding | gastrointestinal | 6 | gastrointestinal rebleeding (page 149); gastrointestinal bleeding; gastric bleed; bleeds | Zhang 2022; Azukaitis 2022; Lin 2018; Natale 2020; McMillan 2015; Bleecker 2020 |
| gastrointestinal disturbance | gastrointestinal | 5 | gastrointestinal disturbances; gastrointestinal distress; GI disturbance | D'Haens 2016; Head 2016b; Head 2016c; Arai 2018; Tunnicliffe 2018 |
| diarrhoea | gastrointestinal | 3 | diarrhoea); diarrhoea | Mithoowani 2016; Natale 2020; Zhang 2024a |
| peptic ulcer | gastrointestinal | 3 | peptic ulcer; peptic ulcer disease | Al Efraij 2019; Mithoowani 2016; Jafarzadeh 2024 |
| vomiting | gastrointestinal | 3 | vomiting | Arai 2018; Mithoowani 2016; Kwak 2022 |
| abnormal liver function | gastrointestinal | 2 | hepatic dysfunction; abnormal liver function | Natale 2020; Zhang 2024a |
| diverticulitis | gastrointestinal | 2 | diverticulitis | Liu 2021; Natale 2020 |
| elevated liver enzymes | gastrointestinal | 2 | elevated liver enzymes | Fujiwara 2019; Mithoowani 2016 |
| gastritis | gastrointestinal | 2 | gastritis | Guan 2024b; Natale 2020 |
| gastrointestinal discomfort | gastrointestinal | 2 | abdominal pain; abdominal discomfort | Natale 2020; Tome 2024 |
| ulcer | gastrointestinal | 2 | duodenal ulcer; GI ulcers | Natale 2020; Bleecker 2020 |
| abdominal discomfort | gastrointestinal | 1 | acute right upper quadrant pain | Natale 2020 |
| abnormal serum albumin | gastrointestinal | 1 | serum albumin | D'Haens 2016 |
| appendicitis | gastrointestinal | 1 | appendicitis | Natale 2020 |
| bowel perforation | gastrointestinal | 1 | bowel perforation | Partridge 2018 |
| constipation | gastrointestinal | 1 | constipation | Fujiwara 2019 |
| faecal occult blood | gastrointestinal | 1 | faecal occult blood | Fujiwara 2019 |
| gastric perforation | gastrointestinal | 1 | gastric perforation | Natale 2020 |
| gastroesophageal reflux | gastrointestinal | 1 | gastroesophageal reflux | Bleecker 2020 |
| heartburn | gastrointestinal | 1 | heartburn | Lin 2018 |
| hepatitis | gastrointestinal | 1 | hepatitis | Atzmony 2014 |
| hepatotoxicity | gastrointestinal | 1 | hepatotoxicity | Natale 2020 |
| hernia | gastrointestinal | 1 | symptomatic incarcerated paraumbilical hernia | Natale 2020 |
| pancreatitis | gastrointestinal | 1 | pancreatitis | Iheozor-Ejiofor 2019 |
| perianal abscess | gastrointestinal | 1 | perianal abscess | Natale 2020 |
| prostatitis | gastrointestinal | 1 | prostatitis | Liu 2021 |
| upper abdominal discomfort | gastrointestinal | 1 | upper abdominal discomfort | Kafil 2017 |
| fatigue | general | 2 | fatigue | Liu 2021; Mithoowani 2016 |
| flushing | general | 2 | facial ?ushing; flushing | D'Haens 2016; Jafarzadeh 2024 |
| organ damage | general | 2 | overall organ damage; organ impairment | Ugarte-Gil 2021; Singh 2023 |
| epistaxis | general | 1 | epistaxis | Akiyama 2020 |
| lethargy | general | 1 | lethargy | Jafarzadeh 2024 |
| serum sickness | general | 1 | serum sickness | Arai 2018 |
| soft tissue injury | general | 1 | soft tissue injury | Natale 2020 |
| anaemia | haematological | 3 | haemoglobin; anaemia | D'Haens 2016; Akiyama 2020; Atzmony 2016 |
| abnormal platelet count | haematological | 1 | platelet count | D'Haens 2016 |
| thrombocytopenia | haematological | 1 | thrombocytopenia | Akiyama 2020 |
| infection | immunological | 26 | phlegmone; other infections; oral ulceration; oral herpes; nocardia infection of skin; nasopharyngitis; knee empyema; influenza; infections (p7); infections; infection; herpes-zoster virus infection; herpes zoster infection; herpes zoster; bacteraemia; and infection; HPV frequency; pyoderma | Akiyama 2020; Partridge 2018; Buttgereit 2016; Natale 2020; Campbell 2020; D'Haens 2016; Abe 2019; Al Efraij 2019; Alchi 2023; Atzmony 2014; Azukaitis 2022; Bleecker 2020; Edel 2020; Lin 2018; Palmowski 2023; Sciascia 2017; Iheozor-Ejiofor 2019; Mithoowani 2016; Singh 2023; Tunnicliffe 2018; Zhang 2024a; Cobo-Ibáñez 2014; Antonio 2022; Raposo 2016; Jafarzadeh 2024; McMillan 2015 |
| serious infection | immunological | 15 | serious infectious events; serious infections; serious infection; serious CNS infections; septic shock post-colonic perforation; sepsis; mortality (sepsis); major infection; infections in white matter; cryptococcal meningitis; cardiogenic shock post-amputation) | Sugihara 2024; Bergstra 2023; Figueroa-Parra 2024; Partridge 2018; Singh 2020; Bose 2022; Buttgereit 2016; Singh 2016; Molooghi 2022; Liu 2021; Atzmony 2014; Singh 2023; Tunnicliffe 2018; Cobo-Ibáñez 2014; Natale 2020 |
| allergic reaction | immunological | 5 | severe drug allergy; late infusion-related reaction; infusion reactions; early infusion reaction; anaphylaxis; allergic reactions. | Natale 2020; Cobo-Ibáñez 2014; Akiyama 2020; Arai 2018; Iheozor-Ejiofor 2019 |
| fever | immunological | 4 | fever | Arai 2018; Liu 2021; Mithoowani 2016; Natale 2020 |
| leukopenia | immunological | 4 | low white cell count; leukopenia; Safety profile of Tocilizumab listed adverse effects including:  Leukopenia | Singh 2023; Iheozor-Ejiofor 2019; McMillan 2015; Akiyama 2020 |
| neutropenia | immunological | 3 | neutropenia | Akiyama 2020; Arai 2018; Liu 2021 |
| adverse event | immunological | 1 | local adverse effects | Zhang 2022 |
| bone marrow toxicity | immunological | 1 | bone marrow toxicity | Natale 2020 |
| change to CRP | immunological | 1 | C-reactive protein | D'Haens 2016 |
| change to erythrocyte sedimentation rate | immunological | 1 | ESR | D'Haens 2016 |
| orosomucoid | immunological | 1 | orosomucoids | D'Haens 2016 |
| pyogenic complication | immunological | 1 | pyogenic complications | D'Haens 2016 |
| viral rash | immunological | 1 | viral exanthema | Natale 2020 |
| fracture | musculoskeletal | 13 | overall fractures; osteoporotic fractures; non-vertebral fractures; non vertebral factures; fractures; fracture risk; fracture | Li 2021; Bergstra 2023; Ugarte-Gil 2021; Amiche 2016; Allen 2016; Al Efraij 2019; Bleecker 2020; Buttgereit 2016; Sugihara 2024; Hmamouchi 2023; Arai 2018; Chalitsios 2021; Natale 2020 |
| osteoporosis | musculoskeletal | 9 | osteoporosis; Osteoporosis | Al Efraij 2019; Bleecker 2020; Chalitsios 2021; Durazzo 2019; Hmamouchi 2023; Ji 2021; McMillan 2015; Winblad 2017; Bergstra 2023 |
| vertebral fracture | musculoskeletal | 7 | vertebroplasty; vertebral fractures; vertebral fracture risk; spontaneous vertebral fractures; risk of vertebral fractures; lumbar fractures | Antonio 2022; Allen 2016; Amiche 2016; Campbell 2020; Chang 2019; Liu 2021; Blavnsfeldt 2018 |
| change in bone mineral density | musculoskeletal | 6 | decreased bone mineral density (lumbar spine); decreased bone mineral density; changes in bone mineral density; bone mineral density at lumbar spine; bone mineral density (p4; bone mineral density | Siu 2015; Amiche 2016; Hysa 2024; Blavnsfeldt 2018; D'Haens 2016; Allen 2016 |
| osteopoenia | musculoskeletal | 4 | osteopoenia; osteopenia | Oaklander 2017; Hmamouchi 2023; Hughes 2017; Kwak 2022 |
| arthralgia | musculoskeletal | 3 | arthralgia | Liu 2021; Mithoowani 2016; Natale 2020 |
| avascular necrosis | musculoskeletal | 3 | avascular necrosis | Azukaitis 2022; Ji 2021; Ugarte-Gil 2021 |
| myopathy | musculoskeletal | 3 | myopathy; immobilizing steroid-induced myopathy | Durazzo 2019; Wu 2022; Antonio 2022 |
| joint swelling | musculoskeletal | 2 | joint swelling; ankle swelling | Natale 2020; Bonovas 2018 |
| musculoskeletal event | musculoskeletal | 2 | musculoskeletal effects; muscle complications | Allen 2016; Al Efraij 2019 |
| myalgia | musculoskeletal | 2 | myalgia | Arai 2018; Mithoowani 2016 |
| osteonecrosis | musculoskeletal | 2 | osteonecrosis of the femoral head; osteonecrosis | Natale 2020; Ugarte-Gil 2021 |
| aseptic necrosis | musculoskeletal | 1 | aseptic necrosis | Sciascia 2017 |
| back pain | musculoskeletal | 1 | back pain | Natale 2020 |
| bone formation | musculoskeletal | 1 | bone formation | D'Haens 2016 |
| change in bone turnover | musculoskeletal | 1 | bone turnover | Blavnsfeldt 2018 |
| elevated CPK | musculoskeletal | 1 | blood creatine phosphokinase increased | Natale 2020 |
| metabolic bone disease | musculoskeletal | 1 | metabolic bone disease | Tome 2024 |
| muscle spasms | musculoskeletal | 1 | muscle spasms | Natale 2020 |
| osteoarthritis | musculoskeletal | 1 | knee osteoarthritis | Akiyama 2020 |
| vascular necrosis | musculoskeletal | 1 | vascular necrosis | Natale 2020 |
| sleep disturbance | neurological | 19 | sleeplessness; sleep disturbances; sleep disturbance; sleep changes (p4); sleep changes; sleep disorders; insomnia); insomnia; disturbed sleep; Insomnia | Hughes 2017; Campbell 2020; Fujiwara 2019; Madhok 2016; Kwak 2022; Oaklander 2017; D'Haens 2016; Bonovas 2018; Bleecker 2020; Head 2016b; Jafarzadeh 2024; Lin 2018; Liu 2021; Mithoowani 2016; Natale 2020; Shiber 2014; Zhang 2024a; Kafil 2017; Lattanzi 2017 |
| headache | neurological | 9 | headache | D'Haens 2016; Jafarzadeh 2024; Kafil 2017; Kwak 2022; Lin 2018; Liu 2021; Natale 2020; Sebastian 2019; Tome 2024 |
| cramps | neurological | 2 | leg cramps; cramps | Kafil 2017; Natale 2020 |
| confusion | neurological | 1 | confusion | D'Haens 2016 |
| fall | neurological | 1 | accidental fall | Arai 2018 |
| hiccups | neurological | 1 | hiccups | Fujiwara 2019 |
| pain | neurological | 1 | pain | Arai 2018 |
| restless legs | neurological | 1 | restless legs | Liu 2021 |
| tremor | neurological | 1 | tremors | Natale 2020 |
| vertigo | neurological | 1 | vertigo | Liu 2021 |
| glaucoma | ophthalmological | 8 | steroid glaucoma; glaucoma | McMillan 2015; Bergstra 2023; Black 2016; Hughes 2017; Oaklander 2017; Sugihara 2024; Tome 2024; Al Efraij 2019 |
| cataract | ophthalmological | 7 | cataracts; cataract | Al Efraij 2019; Black 2016; Bleecker 2020; Sugihara 2024; Tome 2024; Arai 2018; Ugarte-Gil 2021 |
| eye complication | ophthalmological | 3 | ocular complications; eye complications | Al Efraij 2019; Hysa 2024; D'Haens 2016 |
| optic neuritis | ophthalmological | 1 | ophthalmos-neuritis | Natale 2020 |
| anxiety | psychiatric | 4 | anxiety; anxiety | Bleecker 2020; Mithoowani 2016; Kwak 2022; Shiber 2014 |
| depression | psychiatric | 4 | such as depression; depression | Koning 2024; Bleecker 2020; Bonovas 2018; McMillan 2015 |
| mood disturbance | psychiatric | 4 | mood swings; mood disturbances; mood disorders | Bonovas 2018; Natale 2020; Head 2016b; Mithoowani 2016 |
| psychosis | psychiatric | 4 | psychotic episodes; psychotic disorder; psychosis; paranoid psychosis | Durazzo 2019; De Bock 2024; Antonio 2022; McMillan 2015 |
| psychiatric condition | psychiatric | 3 | psychiatric conditions; psychiatric complications; psychiatric | Bleecker 2020; Al Efraij 2019; Edel 2020 |
| mania | psychiatric | 2 | mania; hypomania | De Bock 2024; Koning 2024 |
| neuropsychiatric adverse event | psychiatric | 1 | neuropsychiatric adverse effects | Koning 2024 |
| acute kidney injury | renal | 2 | acute kidney injury; acute kidney injuries | Azukaitis 2022; Natale 2020 |
| end stage kidney disease | renal | 2 | end stage kidney disease; ESKD | Alchi 2023; Natale 2020 |
| chronic kidney disease | renal | 1 | chronic kidney disease | Bleecker 2020 |
| elevated serum creatinine | renal | 1 | doubling of serum creatinine | Azukaitis 2022 |
| renal failure | renal | 1 | renal failure | Azukaitis 2022 |
| uremia | renal | 1 | uremia | Natale 2020 |
| complication of pregnancy | reproductive | 1 | adverse pregnancy | Bergstra 2023 |
| menorrhagia | reproductive | 1 | menorrhagia (p6) | D'Haens 2016 |
| menstrual change | reproductive | 1 | and menstrualvabnormalities | Atzmony 2016 |
| menstrual irregularity | reproductive | 1 | menstrual irregularity | Jafarzadeh 2024 |
| lower respiratory tract infection | respiratory | 7 | pulmonary infection; pulmonary abscess; pulmonary TB; pneumonia; pneumocystis pneumonia; lower respiratory tract infection | Natale 2020; Liu 2021; Arai 2018; Elsouri 2023; Mithoowani 2016; Zhang 2024a; Campbell 2020 |
| cough | respiratory | 2 | cough | Lin 2018; Natale 2020 |
| respiratory infection | respiratory | 2 | respiratory tract infection; respiratory infection | Natale 2020; Zhang 2024a |
| upper respiratory tract infection | respiratory | 2 | upper respiratory tract infection; upper respiratory infection | Natale 2020; Akiyama 2020 |
| bronchiectasis | respiratory | 1 | asthma-related bronchiectasis | Bleecker 2020 |
| bronchitis | respiratory | 1 | bronchitis | Campbell 2020 |
| dyspnoea | respiratory | 1 | dyspnea | Liu 2021 |
| haemoptysis | respiratory | 1 | haemoptysis | Natale 2020 |
| vasculitis | rheumatological | 1 | vasculitis | Arai 2018 |
| adverse event | unspecified | 23 | whilst a number of adverse events were reported; total number of adverse events; total adverse events; total AEs; side effects; overall adverse events; cumulative adverse events; any complications; any adverse events; amongst other side effects; adverse events; adverse effects | Chande 2015; Lin 2023; Mithoowani 2016; Singh 2023; Zhang 2024b; Guan 2024b; Lin 2018; Liu 2021; Al Efraij 2019; Palmowski 2023; Brito-Zerón 2016; Edel 2020; Kafil 2017; Manguso 2016; Mateos-Haro 2023; Narula 2018; Palmowski 2024; Rezaie 2015; Siegels 2021; von Groote 2021; Zhao 2015; Madhok 2016; Omar 2020 |
| withdrawal due to adverse event | unspecified | 13 | withdrawals due to adverse events reported; withdrawals due to adverse events; withdrawals; withdrawal due to adverse events; total number of treatment discontinuations; discontinuation; Withdrawal due to adverse events | Moja 2015; Allen 2016; Atzmony 2014; Chiang 2022; Kafil 2017; Narula 2018; Palmowski 2024; Siegels 2021; Bonovas 2018; Atzmony 2015; Rezaie 2015; Bose 2022; Iheozor-Ejiofor 2019 |
| mortality | unspecified | 12 | mortality; deaths; death; all-cause mortality | Abe 2019; Atzmony 2014; Azukaitis 2022; Bergstra 2023; Chang 2019; Edel 2020; Figueroa-Parra 2024; Allen 2016; Alchi 2023; Lin 2018; Natale 2020; Atzmony 2015 |
| serious adverse event | unspecified | 12 | severe AEs; serious adverse events. Those specifically noted related to glucocorticoids were nausea; serious adverse events; serious adverse event; any serious AEs | Singh 2023; Kafil 2017; Allen 2016; Bose 2022; Edel 2020; Liu 2021; Natale 2020; Palmowski 2024; Siegels 2021; Sugihara 2024; Zhang 2024a; Bonovas 2018 |
| glucocorticoid-related adverse event | unspecified | 2 | glucocorticoid-related adverse effects; glucocorticoid-associated adverse events | Zhang 2019; Rezaie 2015 |
| steroid-related adverse event | unspecified | 2 | steroid-related side effects; and corticosteroid-related AEs (sum of number of reported instances of moon face | Webster 2023; Bonovas 2018 |
| urinary tract infection | urological | 4 | urinary tract infections; urinary tract infection; recurrent UTI; UTI | Cobo-Ibáñez 2014; Campbell 2020; Natale 2020; Zhang 2024a |
